# A Self‐Assembling LYTAC Mediates CTGF Degradation and Remodels Inflammatory Tumor Microenvironment for Triple‐Negative Breast Cancer Therapy

**DOI:** 10.1002/advs.202500311

**Published:** 2025-05-11

**Authors:** Jia‐Yi Lin, Ye Wu, Xiao‐Hui Liang, Min Tang, Xin Sun, Sheng‐Xin Lu, Jin‐Mei Jin, Xin Guo, Bei Wang, Hong‐Zhuan Chen, Wei‐Dong Zhang, Xin Luan

**Affiliations:** ^1^ State Key Laboratory of Discovery and Utilization of Functional Components in Traditional Chinese Medicine Shanghai Frontiers Science Center of TCM Chemical Biology Institute of Interdisciplinary Integrative Medicine Research and Shuguang Hospital Shanghai University of Traditional Chinese Medicine Shanghai 201203 China; ^2^ State Key Laboratory for Quality Ensurance and Sustainable Use of Dao‐di Herbs Institute of Medicinal Plant Development Chinese Academy of Medical Sciences & Peking Union Medical College Beijing 100700 China; ^3^ School of Pharmacy Second Military Medical University Shanghai 200433 China

**Keywords:** connective tissue growth factor, lysosome‐targeting chimeras, self‐assembling nanoparticle, triple‐negative breast cancer, tumor microenvironment

## Abstract

As a multifunctional extracellular protein, connective tissue growth factor (CTGF/CCN2) is significantly associated with the progression and prognosis of triple‐negative breast cancer (TNBC). However, current blockade therapies targeting CTGF's multiple domains are limited, creating substantial challenges in treatment. Lysosome‐targeting chimeras (LYTACs) have emerged as a promising approach for achieving complete protein degradation and inhibiting CTGF's various bioactivities. In this study, a self‐assembling LYTAC nanoplatform, NanoCLY, designed to tumor microenvironment (TME)‐responsively degrade CTGF is presented. The complete degradation of CTGF downregulates the TGF‐β signaling pathway and disrupts the CTGF‐IL‐6 cell crosstalk within the TME, which further inhibits the activation of inflammatory cancer‐associated fibroblasts (CAFs) and alleviates the inflammatory TME. Notably, the anti‐TNBC effect of LYTAC‐based CTGF degradation therapy surpasses that of antibody‐based blockade therapy in both in vitro and in vivo models. The findings provide a proof of concept for CTGF degradation in TNBC and introduce the first CTGF‐LYTAC nanoplatform aimed at TME‐directed therapy.

## Graphical abstract

A CTGF‐LYTAC nanoplatform is developed to selectively degrade connective tissue growth factor (CTGF) in triple‐negative breast cancer (TNBC), inhibiting TGF‐β signaling and cell interactions in the tumor microenvironment (TME). This strategy effectively suppresses tumor growth and metastasis, outperforming antibody‐based therapy. The study provides proof of concept for CTGF degradation and introduces the first CTGF‐LYTAC nanoplatform for TME‐targeted TNBC treatment.

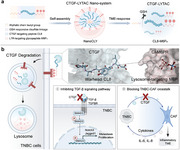

## 1 Introduction

Triple‐negative breast cancer (TNBC), which constitutes about 15% of breast cancer cases, is characterized by high tumor heterogeneity and a lack of effective treatments, resulting in the poorest outcomes among breast cancer subtypes.^[^ ^1^ ^]^ The tumor microenvironment (TME), a complex network of diverse cell types and extracellular matrix (ECM) components, plays a crucial role in TNBC pathogenesis, influencing cancer growth, metastasis, and therapeutic response.^[^ ^2^ ^]^ Recognizing this, TME‐targeted strategies such as antiangiogenic therapy and immunotherapy have shown potential, with some approaches already advancing to clinical trials or approval.^[^ ^2^, ^3^, ^4^, ^5^, ^6^, ^7^ ^]^

The ECM in solid tumors undergoes spatial and temporal alterations, which are now considered hallmarks of cancer.^[^ ^8^ ^]^ In TNBC, connective tissue growth factor (CTGF/CCN2) is significantly overexpressed and has been linked to tumor progression.^[^ ^9^ ^]^ CTGF, a member of the CCN protein family, has a unique structure with four conserved domains: the insulin‐like growth factor‐binding protein (IGFBP), von Willebrand factor type C repeat (VWC), thrombospondin type 1 repeat (TSP), and the cysteine knot‐containing carboxyl domain (CT) (Figure S1a, Supporting Information).^[^ ^10^ ^]^ This structure enables CTGF to support TNBC cell proliferation, migration, angiogenesis, and cellular crosstalk within the TME.^[^ ^9^, ^11^, ^12^, ^13^ ^]^ Given the multiple roles of CTGF in TNBC, targeted CTGF antagonism could be a promising strategy for therapy. The FG‐3019 (pamrevlumab), an anti‐CTGF monoclonal antibody, has shown potential in phase I/II clinical trials without added toxicity when combined with chemotherapy for pancreatic carcinoma.^[^ ^14^ ^]^ However, the flexible hinge region in CTGF is susceptible to proteolytic cleavage, which separates CTGF into N‐terminal and C‐terminal domains, each exhibiting distinct pro‐tumor effects.^[^ ^15^ ^]^ The FG‐3019 primarily targets the VWC domain of CTGF, partially inhibiting downstream signaling from the N‐terminal domain.^[^ ^16^ ^]^ This limitation may lead to the unsatisfactory efficacy of antibodies in CTGF antagonism. Therefore, the lack of effective targeted therapies capable of comprehensively inhibiting multiple CTGF domains presents a significant challenge in TNBC treatment.

Targeted protein degradation (TPD) is an emerging therapeutic modality that enables the targeting of previously undruggable proteins by harnessing the inherent degradation system.^[^ ^17^ ^]^ With proteolysis targeting chimeras (PROTACs) now in clinical trials, the TPD field is experiencing significant growth.^[^ ^18^ ^]^ Unlike PROTACs, which primarily target cytoplasmic proteins, lysosome‐targeting chimeras (LYTACs) can degrade extracellular and membrane‐associated proteins of interest (POI).^[^ ^19^ ^]^ LYTACs are heterobifunctional conjugates composed of a POIs‐binding ligand and a lysosome‐targeting receptor (LTR)‐binding moiety. This design facilitates the attachment of POI to cell‐surface LTR, leading to protein internalization and subsequent degradation via the endo‐lysosomal pathway.^[^ ^20^ ^]^ In contrast to traditional inhibitors that function on an “occupancy‐driven” basis, LYTACs utilize “event‐driven” pharmacology to achieve complete degradation of proteins like CTGF and inhibit their multiple functions.^[^ ^21^ ^]^ Thus, this innovative approach holds significant promise for enhancing therapeutic strategies against TNBC by effectively targeting CTGF and potentially improving treatment outcomes.

**Scheme 1:**
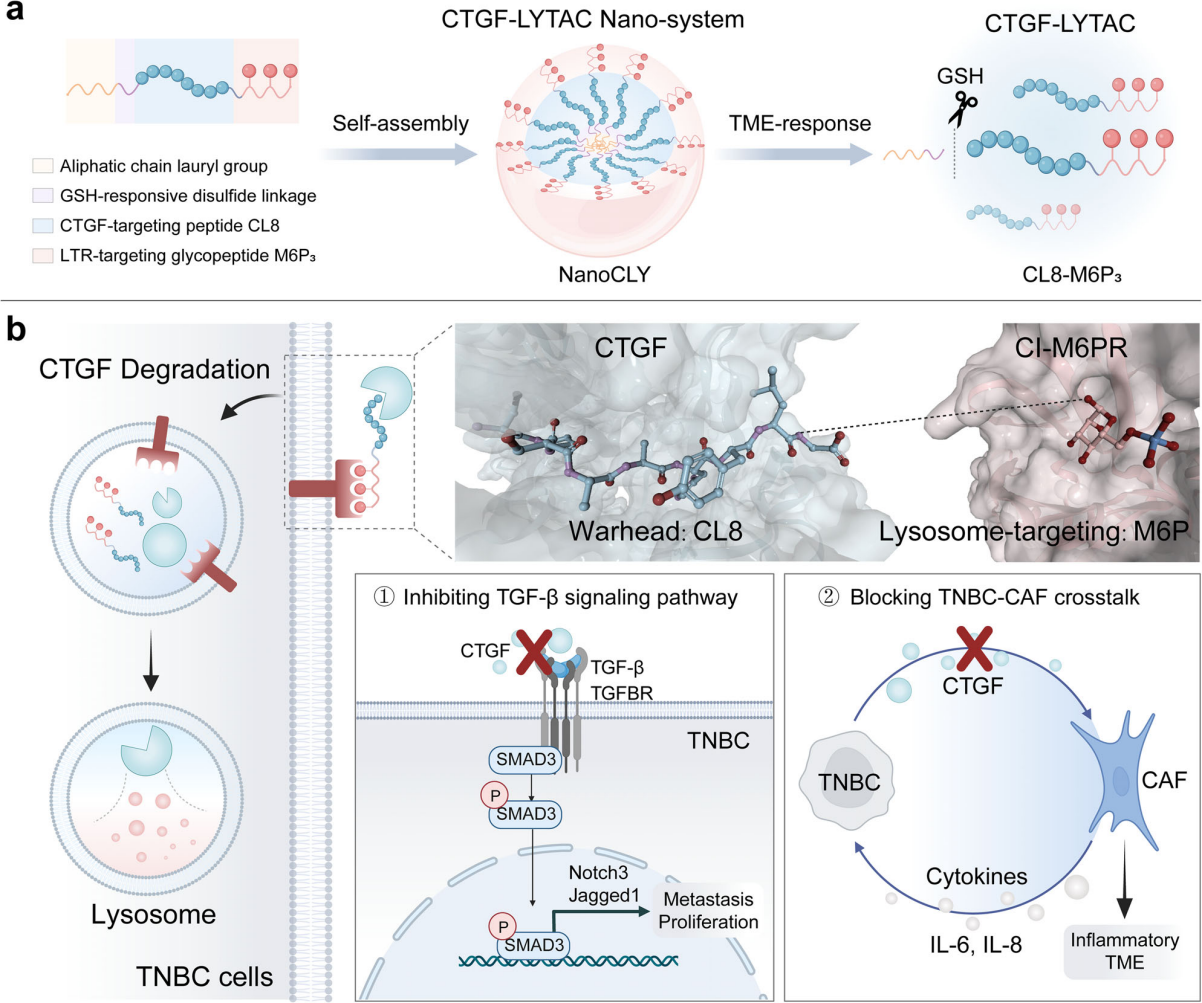
Schematic illustration of Nano‐LYTAC‐induced CTGF degradation for TNBC therapy. a) Design, synthesis, and TME‐responsive LYTAC release of Nano‐LYTAC. b) The CTGF‐LYTAC, CL8‐M6P_3_, facilitates the attachment of CTGF to CI‐M6PR, leading to CTGF internalization and degradation in TNBC cells via the endo‐lysosomal pathway. The depletion of CTGF downregulates the downstream TGF‐β signaling pathway to inhibit TNBC growth and metastasis, and disrupts the TNBC‐CAF cell crosstalk to alleviate the inflammatory TME.

The design of CTGF‐LYTAC involves a moiety for the recognition of LTRs, such as the cation‐independent mannose‐6 phosphate receptor (CI‐M6PR/IGF2R), alongside a warhead specifically targeting CTGF (**Scheme** **1**). To bind CI‐M6PR, we designed a novel glycopeptide scaffold M6P_3_, which contains three mannose‐6‐phosphates (M6P) and has a moderate molecular weight compared to the poly‐M6P used in the first‐generation LYTAC^[^ ^19^ ^]^ or the fusion protein in EndoTags.^[^ ^22^ ^]^ For targeting CTGF, an octapeptide CL8^[^ ^23^ ^]^ was screened, based on its high affinity with CTGF. Peptides like CL8 offer advantages over antibodies and fusion proteins, including lower molecular weight and greater modifiability. By conjugating M6P_3_ with CL8 using a PEG_3_ linker, we developed a peptide‐based CTGF‐LYTAC termed CL8‐M6P_3_. To enhance selective CTGF degradation at the tumor sites while minimizing side effects, we further developed a CTGF‐LYTAC nanoplatform based on CL8‐M6P_3_. This involved connecting the hydrophilic CL8‐M6P_3_ with a hydrophobic aliphatic chain via a disulfide linkage, resulting in a self‐assembling glutathione (GSH)‐responsive LYTAC nanoparticle, termed NanoCLY. Encouragingly, this CTGF‐LYTAC nanoplatform demonstrated several key capabilities: (i) it enabled tumor‐targeted accumulation and TME‐responsive lysosomal degradation of CTGF; (ii) the depletion of CTGF downregulated the downstream signaling pathways in TNBC cells, disrupted the TNBC/cancer‐associated fibroblasts (CAFs) interactions, and inhibited TNBC growth and metastasis both in vitro and in vivo. Our findings offer crucial insights into the multifaceted roles of CTGF in TNBC progression and highlight the potential of the novel CTGF‐LYTAC strategy for TME‐directed therapies.

## 2 Results and Discussion

### 2.1 Expression of CTGF in TNBC and Its Clinical Significance

**Figure 1:**
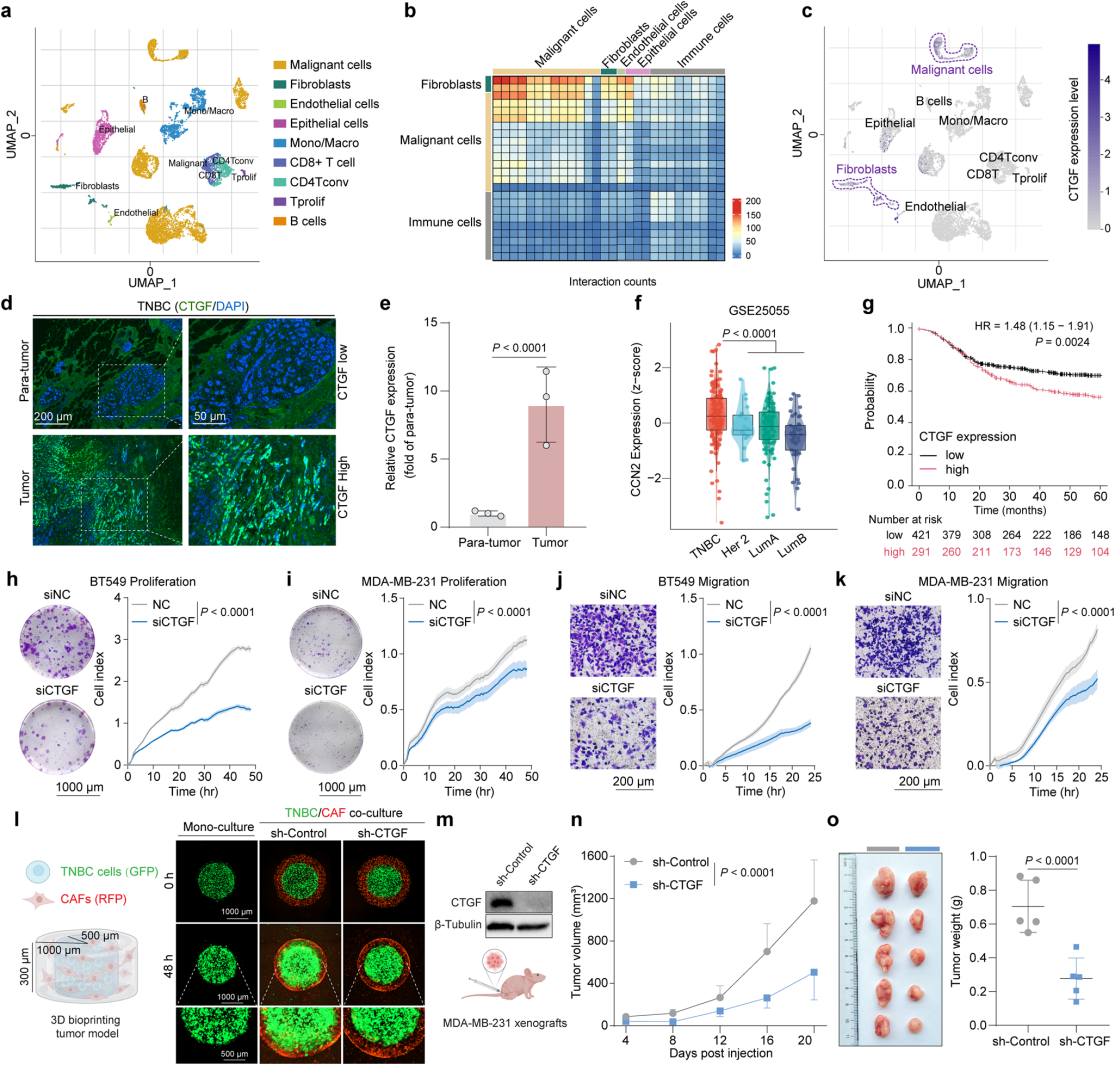
The clinical significance of CTGF in TNBC. a) Uniform Manifold Approximation and Projection (UMAP) plots illustrating subgroups of TNBC (http://tisch.comp‐genomics.org/). b) Cell‐cell interactions between different cell clusters within the TNBC TME. c) UMAP plots of CTGF. d, e) Immunofluorescence images and related statistics showing CTGF expression levels in tumor tissues and para‐tumorous TNBC tissues. f) CTGF mRNA expression values of breast cancer subtypes by a biomarker exploration tool (https://rookieutopia.com/). g) Survival analysis of TNBC patients based on CTGF expression levels of a public database by Kaplan‐Meier plotter (https://kmplot.com/analysis/). h–k) Images and kinetic curves of the proliferation and migration of BT549 and MDA‐MB‐231 cells after siRNA transfection, as assessed by xCELLigence RTCA‐DP (*n* = 4). The P values were calculated by two‐way ANOVA. l) Immunofluorescence images of the monoculture TNBC models and TNBC/CAF co‐culture 3D bioprinting models. TNBC cells were labeled with a green fluorescent protein (GFP), while CAFs were labeled with a red fluorescent protein (RFP). m) Western blotting analysis of CTGF level in MDA‐MB‐231 cells (sh‐Control and sh‐CTGF). n, o) Tumor growth, photographs of excised tumors, and tumor weight (n = 5). The P values were calculated by one‐way and two‐way ANOVA. For (e, f, h‐k, n, o), the quantified data from different experiments were presented as the mean ± SD.

TNBC is known for its aggressive nature and significant intratumoral heterogeneity.^[^ ^24^ ^]^ To investigate the complex microenvironment of TNBC, we analyzed publicly available single‐cell RNA sequencing data. Using the Uniform Manifold Approximation and Projection (UMAP) algorithm, we identified 9 distinct cell clusters (**Figure** **1**a). Cell–cell interactions (CCI), which facilitate communication among neighboring cells within the TME, play a vital role in TNBC progression. Our analysis of the CCI network associated with protumor activities revealed that malignant TNBC cells exhibited the closest and most frequent interactions with CAFs (Figure 1b). Notably, both TNBC cells and CAFs highly expressed the multifunctional ECM protein CTGF among the cell clusters (Figure 1c). Immunofluorescence (IF) analysis of biopsy specimens from TNBC patients demonstrated significantly elevated levels of CTGF in tumor tissues compared to para‐carcinoma tissues (Figure 1d,e). Furthermore, CTGF expression was found to be higher in TNBC compared to other breast cancer subtypes (Figure 1f). Kaplan–Meier survival analysis indicated that elevated CTGF expression correlates with poor survival outcomes in TNBC patients (Figure 1g). These analyses suggested that the increased CTGF expression is highly associated with TNBC progression and may serve as both a prognostic biomarker and a potential therapeutic target.

To further dissect the role of CTGF in TNBC, we knocked down CTGF in human TNBC cell lines, BT549 and MDA‐MB‐231. Western blotting analysis confirmed a significant depletion of CTGF in both cell lines (Figure S1d,e, Supporting Information). We employed three approaches to assess the relevance of CTGF to TNBC progression. Firstly, using a Real‐Time Cell Analysis (RTCA) system and colony formation assay, we observed that the knockdown of CTGF reduced both cell proliferation and colony formation ability in BT549 and MDA‐MB‐231 cells (Figure 1h,i). Similarly, the knockdown of CTGF resulted in decreased cell migration in both cell lines (Figure 1j,k). Secondly, 3D tumor models maintain essential in vivo characteristics of the TME and mimic the molecular behavior of cancer cells. Recognizing the significant role of CAFs in tumor progression through interactions with TNBC cells and ECM remodeling, we generated a 3D TNBC/CAF co‐culture model using a digital light processing‐based rapid 3D bioprinting system. Our observations showed that TNBC/CAF co‐culture led to increased invasion of BT549 cells compared to mono‐culture, while CTGF knockdown further inhibited TNBC cell invasion (Figure 1l). Thirdly, MDA‐MB‐231 cells (sh‐Control and sh‐CTGF) were subcutaneously injected into nude mice (Figure 1m). CTGF knockdown significantly inhibited tumor growth in vivo, reinforcing its pro‐tumor effects in TNBC (Figure 1n,o). These results confirmed that increased CTGF expression is closely associated with TNBC proliferation and metastasis, indicating that a CTGF‐targeting strategy could be valuable for TNBC treatment. However, consistent with previous reports,^[^ ^15^ ^]^ we observed that matrix metalloproteinases in the TNBC TME led to the dissociation of CTGF (Figure S1b, Supporting Information). The released CTGF‐cleaved fragments remained individual activities^[^ ^10^ ^]^ and could not all be recognized by the antibody (Figure S1c, Supporting Information), posing a significant challenge for CTGF‐targeting therapy.

### 2.2 Design and Synthesis of CL8‐M6P_3_ as a Potent Degrader of CTGF

**Figure 2:**
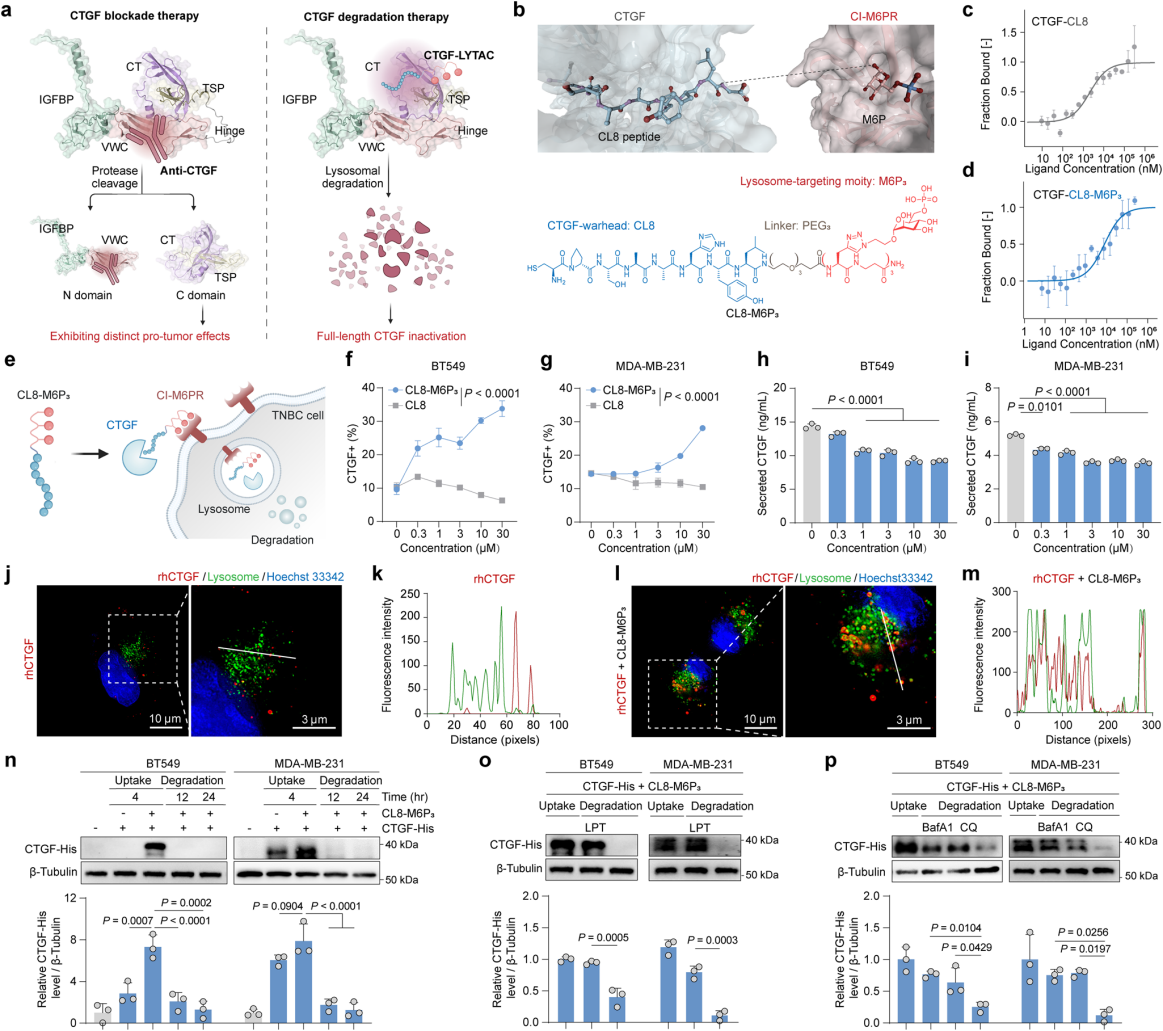
M6P_3_‐based LYTAC mediates CTGF to lysosomes for degradation. a) The comparison of CTGF blockade therapy and CTGF degradation therapy. b) Computer simulation of appropriate site for tethering CL8 to M6P for the design of CTGF‐LYTAC using CABS‐dock server (https://biocomp.chem.uw.edu.pl/CABSdock/, CTGF AlphaFoldDB ID: AF‐P29279‐F1; N‐terminal 3 domains of CI‐M6PR PDB ID: 1SYO), and structure of CL8‐M6P_3_. c,d) MST analysis showing binding interactions between CTGF and CL8, and interactions between CTGF and CL8‐M6P_3_. e) Internalization of CTGF mediated by CL8‐M6P_3_. f,g) FCM analysis of BT549 and MDA‐MB‐231 cells incubated with CTGF and CL8‐M6P_3_ or CL8 for 6 h (*n* = 3). The *P* values were calculated by two‐way ANOVA. h,i) ELISA of CTGF protein levels in culture media of BT549 and MDA‐MB‐231 cells incubated with CL8‐M6P_3_ for 12 h. The *P* values were calculated by one‐way ANOVA. j–m) Live‐cell confocal microscopy images of BT549 cells treated with CTGF and CL8‐M6P_3_ for 4 h, followed by labeling with LysoTracker Green for 30 min, and colocalization analysis of CTGF and lysosomes. *n*) The effects of CL8‐M6P_3_ on CTGF protein uptake and decrease in BT549 and MDA‐MB‐231 cells (*n* = 3). o,p) Western blot analysis of CTGF levels in BT549 and MDA‐MB‐231 cells treated with 1 × 10^−5^ m CL8‐M6P_3_ for 12 h along with 0.1 mg mL^−1^ LPT, 5 × 10^−8^ m bafA1 or 1 × 10^−5^ m CQ (*n* = 3). The *P* values were calculated by one‐way ANOVA. For (f–i, n–p), the quantified data from different experiments were presented as the mean ± SD.

In response to the need for effective CTGF targeting blockade therapies, we proposed a novel CTGF degradation strategy utilizing a bifunctional CTGF‐LYTAC (**Figure** **2**a,b). This construct consists of three components: a CI‐M6PR‐binding motif, a polyethylene glycol (PEG) linker, and a CTGF‐binding motif. Inspired by poly‐mannose‐6‐phosphate (M6P_n_), a lysosome‐targeted ligand of the 1^st^ LYTAC, we designed a novel peptide scaffold bearing M6P, characterized by its accurate and low molecular weight, as well as reduced synthesis steps. Glycopeptides, Biotin‐M6P*_n_* (*n* = 1, 2, and 3) (Figure S2a–l, Supporting Information), were synthesized using solid‐phase peptide synthesis and click chemistry, and subsequently analyzed by high‐performance liquid chromatography (HPLC) and mass spectrometry (MS) (Figure S2b,c,f,g,j,k, Supporting Information).

To identify the optimal number of M6P residues for LYTAC internalization, we performed a proof‐of‐concept study using NeutrAvidin Protein Dylight 650 (NA650) as an extracellular POI. BT549 cells were incubated with 1 × 10^−6^ m Biotin‐M6P*_n_* (*n* = 1, 2, 3) and 1 × 10^−7^ m NA650 for 6 h, followed by fluorescence microscopy and flow cytometry (FCM) analysis (Figure S3a–c, Supporting Information). The results indicated that Biotin‐M6P_3_ facilitated the highest accumulation of NA650 in BT549 cells compared to Biotin‐M6P_1_ and Biotin‐M6P_2_. Thus, we chose Biotin‐M6P_3_ for the subsequent experiments. BT549 cells were treated with NA650 and Biotin‐M6P_3_ at a range of concentrations for 6 h, or with a constant 1 × 10^−6^ m concentration over different time intervals, followed by FCM analysis (Figure S3d–g, Supporting Information). The FCM results indicated that Biotin‐M6P_3_ mediated NA650 uptake in a time‐ and dose‐dependent manner. CI‐M6PR knockdown and coincubation with exogenous ligands (M6P and Biotin) inhibited NA650 uptake, demonstrating the dependence on the formation of the ternary complex (NA650‐Biotin‐M6P_3_‐CI‐M6PR) (Figure S3h–l, Supporting Information). In addition, a hook effect was observed at high concentrations of Biotin‐M6P_3_, further supporting the formation of the ternary complex (Figure S3m, n, Supporting Information). Meanwhile, treatment with chloroquine (CQ) and bafilomycin A1 (BafA1) reduced cell uptake of NA650, indicating reliance on lysosomal acidification (Figure S3k,l, Supporting Information). To investigate the subcellular localization of endocytosed NA650, we labeled lysosomes with Lysotracker Green and observed strong colocalization of NA650 with lysosomes (Figure S3o, p, Supporting Information). To evaluate the degradation of NA650, BT549 cells were co‐cultured with NA650 and Biotin‐M6P_3_ for 4 h, followed by fresh media replacement to allow further degradation. Gel fluorescence analysis showed a decrease in total NA650 levels after 8 and 24 h compared to levels after 4 h of co‐incubation (Figure S3q, Supporting Information). In contrast to the proteasome inhibitor MG132, the addition of lysosome protease inhibitor leupeptin (LPT) reversed NA650 degradation at 12 h (Figure S3r, Supporting Information). Taken together, these results demonstrated that the M6P_3_‐based LYTAC platform induced extracellular protein internalization and degradation based on the lysosomal pathway. Finally, the FCM results showed that this platform was effective in various TNBC cell lines (Figure S3s, t, Supporting Information), demonstrating its universality.

We then sought to apply the M6P_3_‐based LYTAC strategy to degrade the secreted protein CTGF. Following extensive literature research and CTGF affinity‐based peptide screening via MicroScale Thermophoresis (MST) (Figure S4, Supporting Information), we selected the CL8 peptide from the bacteriophage display library^[^ ^23^ ^]^ as the CTGF target warhead, demonstrating a Kd value of (1.23 ± 1.52) × 10^−6^ m (Figure 2c). Subsequently, based on molecular docking studies, the C‐terminus of CL8 was chosen to link with the M6P_3_ scaffold via a PEG_3_, creating CL8‐M6P_3_ as a CTGF degrader (Figure 2b, Figure S5a–d, Supporting Information). In addition, global docking analysis revealed that CL8 binds CTGF at the interface between its N‐terminal and C‐terminal regions (Figure S6a, Supporting Information). MST analysis confirmed that CL8‐M6P_3_ interacts with the IGFBP domain at the N‐terminus and the TSP domain at the C‐terminus of CTGF (Figure S6b,c, Supporting Information). This unique binding mechanism suggests that CL8‐M6P_3_ may achieve more effective protein degradation compared to antibodies.

### 2.3 CTGF‐LYTAC Induces Lysosome‐Dependent Degradation of CTGF Protein

With CL8‐M6P_3_ in hand, we firstly measured the interactions between CTGF and CL8‐M6P_3_ using the MST assay, which confirmed that CL8‐M6P_3_ retained a similar affinity of CL8 with CTGF, with a Kd value of (1.34 ± 1.26) × 10^−5^ m (Figure 2d). We next investigated whether CL8‐M6P_3_ mediates the endocytosis of fluorescently labeled recombinant human CTGF‐His tag. BT549 and MDA‐MB‐231 cells were incubated with 2 × 10^−8^ m CTGF and CL8‐M6P_3_ or CL8 for 6 h, followed by FCM analysis. This demonstrated that CL8‐M6P_3_ induced dose‐dependent cell uptake of extracellular CTGF in both cell lines (Figure 2e‐g). We sought to verify the degradation of extracellular CTGF mediated by CL8‐M6P_3_ using enzyme‐linked immunosorbent assay (ELISA). Treatment with CL8‐M6P_3_ led to a significant reduction of extracellular CTGF in both BT549 and MDA‐MB‐231 cells (Figure 2h,i). Notably, CL8‐M6P_3_ selectively targeted CTGF without affecting other CCN family proteins, confirming its on‐target specificity (Figure S7a,b, Supporting Information). As expected, we observed strong colocalization of CTGF with lysosomes in BT549 cells (Figure 2j–m). These data demonstrated that CL8‐M6P_3_ could promote the internalization of CTGF via the endo‐lysosomal pathway. We also observed the CTGF uptake mediated by CL8‐M6P_3_ within 4 h, and the following effective degradation effects at both 12 and 24 h (Figure 2n). In contrast, minimal CTGF degradation was observed without CL8‐M6P_3_ (Figure S8, Supporting Information). To further investigate the degradation mechanism, we treated BT549 and MDA‐MB‐231 cells with CTGF and CL8‐M6P_3_ in the presence or absence of lysosome inhibitors (LPT, BafA1, or CQ). After 12 h, the degradation of CTGF was significantly inhibited by these inhibitors (Figure 2o,p), confirming that CTGF degradation occurred through the lysosomal pathway.

Compared to conventional LYTACs, which consist of a target‐binding antibody and a TLR‐targeting poly‐glycopeptide, short peptide‐based LYTACs have a precisely defined structure with relatively low molecular weight, making them more versatile for chemical synthesis and modifications. However, we observed the hook effect in the NA650 uptake experiment of Biotin‐M6P_3_ in BT549 cells, suggesting the formation of a ternary complex NA‐650/Biotin‐M6P_3_/CI‐M6PR. Similar findings have been reported in previous LYTAC studies.^[^ ^25^, ^26^ ^]^ The hook effect is related to the concentrations of the three components and the equilibrium dissociation of two binary complexes.^[^ ^27^ ^]^ This explains why we did not observe a similar effect of Biotin‐M6P_3_ across different TNBC cell lines (Figure S3s,t, Supporting Information) and of CL8‐M6P_3_ in TNBC cell lines (Figure 2f–i). The variations in CI‐M6PR expression and the differing binding affinities of NA650/Biotin and CTGF/CL8 contributed to these differences. Further characterization and understanding of the hook effect mechanisms are needed. The recent reports can shed some light. For example, to avoid the inefficacy of degraders induced by the hook effect, Wang's group developed a novel nano‐TPD platform utilizing an in situ self‐assembly strategy designed to counteract these effects.^[^ ^28^ ^]^

### 2.4 CTGF‐LYTAC Inhibits TNBC Proliferation and Migration by Inducing CTGF Degradation

**Figure 3:**
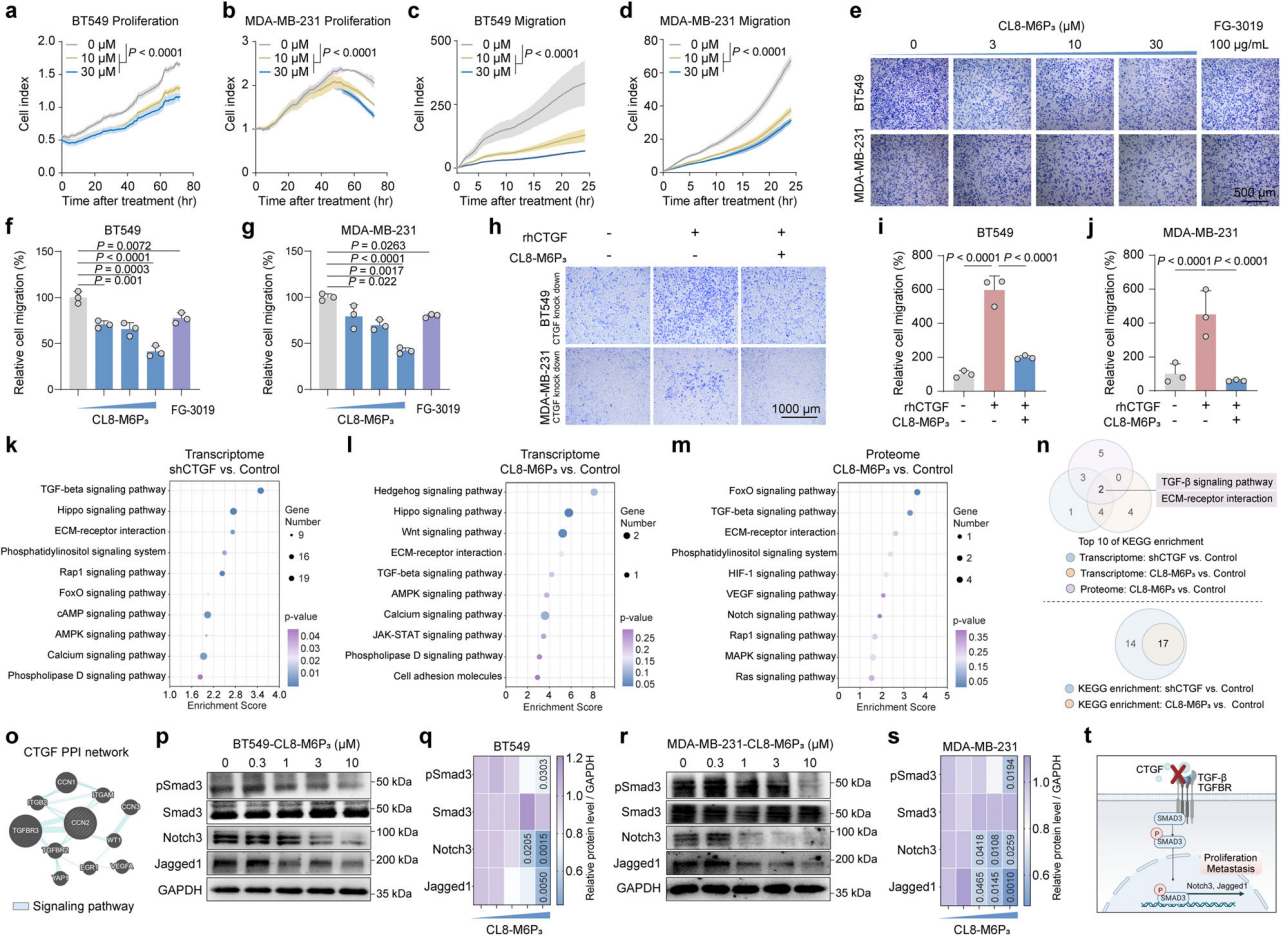
CTGF‐LYTAC inhibits TNBC proliferation and migration in vitro. a–d) Kinetic curves of the proliferation and migration of BT549 and MDA‐MB‐231 cells after CL8‐M6P_3_ treatment, as assessed by xCELLigence RTCA‐DP (*n* = 3). The *P* values were calculated by two‐way ANOVA. Representative images and statistics of transwell migration assays of e–g) BT549 and MDA‐MB‐231 cells following CL8‐M6P_3_ treatment and of h–j) BT549 and MDA‐MB‐231 cells (CTGF‐knockdown) treated with CTGF in the absence and presence of CL8‐M6P_3_ (*n* = 3). The P values were calculated by one‐way ANOVA. Top 10 KEGG signaling pathways of transcriptomics in BT549 cells after k) CTGF knockdown and l) CL8‐M6P_3_ treatment, and that of proteomics in BT549 after m) CL8‐M6P_3_ treatment. n) Venn plot of KEGG signaling pathways in (k‐m). o) Protein–protein interactions (PPI) of CTGF by GeneMANIA. p–t) CL8‐M6P_3_ treatment reduced levels of p‐Smad3, Notch3, Jagged1 of TGF‐β signaling pathway in BT549 and MDA‐MB‐231 cells after 12 h (*n* = 3). The *P* values were calculated by one‐way ANOVA. For (a–d,f,g,i,j), the quantified data from different experiments were presented as the mean ± SD.

With the confirmation of CTGF overexpression in TNBC and its role in tumor progression, we investigated the effects of CL8‐M6P_3_ on TNBC cells based on promising results from the CTGF degradation study. The RTCA results showed that CL8‐M6P_3_ significantly inhibited cell proliferation and migration in BT549 and MDA‐MB‐231 cells (**Figure** **3**a–d). Notably, CL8‐M6P_3_ treatment exhibited better anti‐migration effects compared to FG‐3019 in the transwell assay (Figure 3e–g). To evaluate the on‐target effects of CL8‐M6P_3_, we cultured CTGF‐knockdown TNBC cells with 5 × 10^−8^ m CTGF in the presence and absence of 5 × 10^−6^ m CL8‐M6P_3_, and performed a transwell assay. The results indicated that CL8‐M6P_3_ inhibited the pro‐migration effects of CTGF in TNBC cells (Figure 3h–j). These results supported the concept that the anti‐TNBC activity of CL8‐M6P_3_ arises from CTGF‐targeting degradation. Based on the observed difference between degradation efficiency and migration inhibition, CL8‐M6P_3_ may exert its effect through both 1) targeted degradation of CTGF protein via the LYTAC mechanism and 2) blockade of CTGF activity by the CL8 warhead itself. To explore alterations in signaling pathways after CTGF degradation, we performed global transcriptomic and proteomic profiling of CL8‐M6P3‐treated BT549 cells, focusing on CTGF degradation‐related signaling pathways. As CTGF is a multifunctional protein, both transcriptomic and proteomic analyses revealed that CTGF knockdown by shRNA and degradation by CL8‐M6P_3_ led to the downregulation of multiple signaling pathways, regulating cell proliferation, invasion, receptor interaction, and angiogenesis. The top 10 enriched downregulated KEGG pathways on environmental information processing from these analyses (CL8‐M6P_3_ versus Control) (Figure 3l,m) intersected with pathways from transcriptomic analysis of CTGF knockdown (Figure 3k), identifying two key pathways: the transforming growth factor (TGF)‐β signaling pathway and ECM receptor interaction (Figure 3n). Additionally, the Venn diagram of downregulated KEGG pathways enriched by CTGF knockdown and CL8‐M6P_3_ further revealed the on‐target effects of CL8‐M6P_3_ (Figure 3n). Figure 3o showed strong protein‐protein interactions (PPIs) between CTGF and TGF‐β signaling pathway‐related proteins. CTGF interacts with latent TGF‐β, and engages with TGFβR3 on the cell surface, activating downstream signaling pathways.^[^ ^29^ ^]^ To assess the regulation of the TGF‐β signaling pathway by CL8‐M6P_3_, BT549, and MDA‐MB‐231 cells were treated with CL8‐M6P_3_, and the cell lysates were analyzed by immunoblotting. CL8‐M6P_3_ treatment effectively reduced levels of proteins involved in the TGF‐β signaling pathway, including p‐Smad3, Notch3, and Jagged1 (Figure 3p–t). Taken together, the overlap between CL8‐M6P_3_‐ and CTGF knockdown‐associated pathways underscores the specificity and efficacy of targeted degradation strategies in modulating oncogenic signaling networks. Furthermore, the broad downregulation of multiple pathways following CL8‐M6P_3_ treatment suggests that extracellular degradation of CTGF may reprogram both intracellular signaling pathways and extracellular interactions, thereby inhibiting cell migration and proliferation and contributing to the remodeling of the TME.

### 2.5 CTGF‐LYTAC Blocks Cell–Cell Crosstalk between TNBC Cells and CAFs

**Figure 4:**
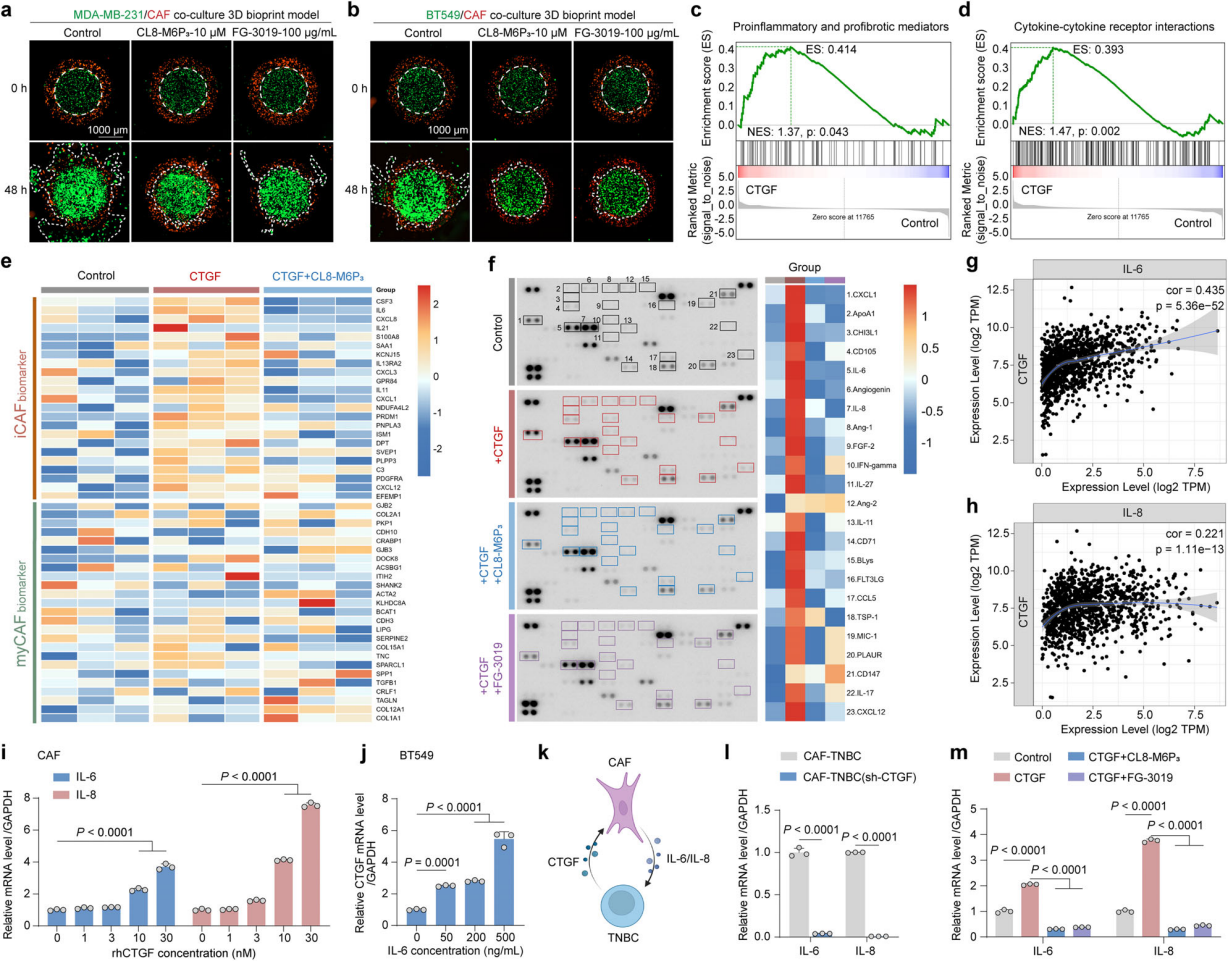
CL8‐M6P_3_ blocks communication between malignant cells and CAFs. a,b) Immunofluorescence images of BT549/CAF and MDA‐MB‐231/CAF coculture 3D bioprinting models after CL8‐M6P_3_ or FG‐3019 treatment. c,d) GSEA showed enriched pathways in breast CAFs after CTGF treatment. e) The heatmap of DEGs related to iCAF and myCAF biomarkers in breast CAFs after CTGF treatment, with or without CL8‐M6P_3_ (*n* = 3). f) Cytokines assays of CAFs after treatment as (e). g, h) Correlation analysis of IL‐6 and CTGF, as well as IL‐8 and CTGF, in breast cancers using a public data (http://timer.comp‐genomics.org/timer/). i) IL‐6 and IL‐8 expression levels in breast CAFs after CTGF treatment (*n* = 3). j) CTGF expression in BT549 after IL‐6 treatment (*n* = 3). k) A schematic diagram showing CTGF‐mediated cellular crosstalk between TNBC cells and CAFs. l) CTGF knockdown decreased IL‐6 and IL‐8 expression in breast CAFs within co‐culture models (*n* = 3). m) CL8‐M6P_3_, and FG‐3019 treatment decreased IL‐6 and IL‐8 expression in CAFs within mono‐culture models (*n* = 3). The P values were calculated by one‐way ANOVA. For (i,j,l,m), the quantified data from different experiments were presented as the mean ± SD.

Based on the enriched KEGG pathway of ECM‐receptor interaction and the frequent communication between TNBC cells and CAFs, we then investigated the effect of CL8‐M6P_3_ in the TME using 3D bioprinting TNBC/CAF coculture model. Following treatment with CL8‐M6P_3_ or FG‐3019, we observed that CL8‐M6P_3_ inhibited TNBC tumor invasion comparable to FG‐3019 in both BT549/CAF and MDA‐MB‐231/CAF co‐culture models (**Figure** **4**a,b). To explore the relationship among CTGF, CAFs, and TNBC cells, we firstly investigated the role of CTGF in CAFs. We performed a gene set enrichment analysis (GSEA) using a transcriptomic profiling of CAFs (CTGF treatment vs Control), which revealed a positive correlation between CTGF and the activation of proinflammatory and profibrotic pathways, as well as cytokine–cytokine receptors interactions (Figure 4c,d). CAFs exhibit heterogeneity and phenotypic plasticity, involving transitions between different states or phenotypes, such as quiescent fibroblasts, inflammatory CAFs (iCAFs), and myofibroblasts (myCAFs).^[^ ^30^ ^]^ The iCAFs and myCAFs are characterized by distinct inflammatory secretomes or matrix‐producing contractile phenotypes, both of which contribute to cancer progression in different ways. To further elucidate the polarization of CAFs mediated by CTGF and CL8‐M6P_3_, we performed global transcriptomic profiling of CAFs treated with CTGF in the absence or presence of CL8‐M6P_3_. The heatmap showed that CTGF significantly promoted iCAF activation, while CL8‐M6P_3_ reversed this effect, reducing the expression of protumorigenic cytokines such as interleukin (IL)‐6, C‐X‐C motif chemokine ligand (CXCL)8/IL‐8, CXCL1, and CXCL3, etc. (Figure 4e).^[^ ^31^ ^]^ Additionally, cytokine profiling of conditioned media from CAFs revealed increased release of several proinflammatory cytokines due to CTGF stimulation. Consistent with our transcriptomic findings, both CL8‐M6P_3_ and FG‐3019 effectively inhibited this cytokine release (Figure 4f). These findings indicated that CTGF is a key regulator of inflammatory CAF polarization and contributes to the establishment of a pro‐tumorigenic TME. Targeted degradation of CTGF by CL8‐M6P_3_ may therefore serve as a promising strategy to suppress CAF‐derived cytokine networks and mitigate tumor‐promoting stromal signaling. Public data on breast cancer patients showed that CTGF levels is positively correlated with IL‐6 and IL‐8 (Figure 4g,h). Moreover, recombinant CTGF increased IL‐6 and IL‐8 expression in CAFs in a dose‐dependent manner (Figure 4i), while recombinant IL‐6 elevated CTGF expression in BT549 cells (Figure 4j). Based on these observations, we hypothesized the existence of a CTGF‐IL‐6/IL‐8 positive feedback loop between TNBC cells and CAFs within the TNBC microenvironment (Figure 4k). As expected, CAFs co‐cultured with CTGF^knockdown^ BT549 cells exhibited significantly lower IL‐6 and IL‐8 expression compared to those co‐cultured with CTGF^full‐length^ BT549 cells (Figure 4l). Moreover, CL8‐M6P_3_ treatment effectively reduced IL‐6 and IL‐8 expression in CAFs, indicating that CL8‐M6P_3_ could block the cell crosstalk between TNBC and CAFs through CTGF degradation (Figure 4m).

### 2.6 Construction, Characterization, and Tumor Targeting of NanoCLY

**Figure 5:**
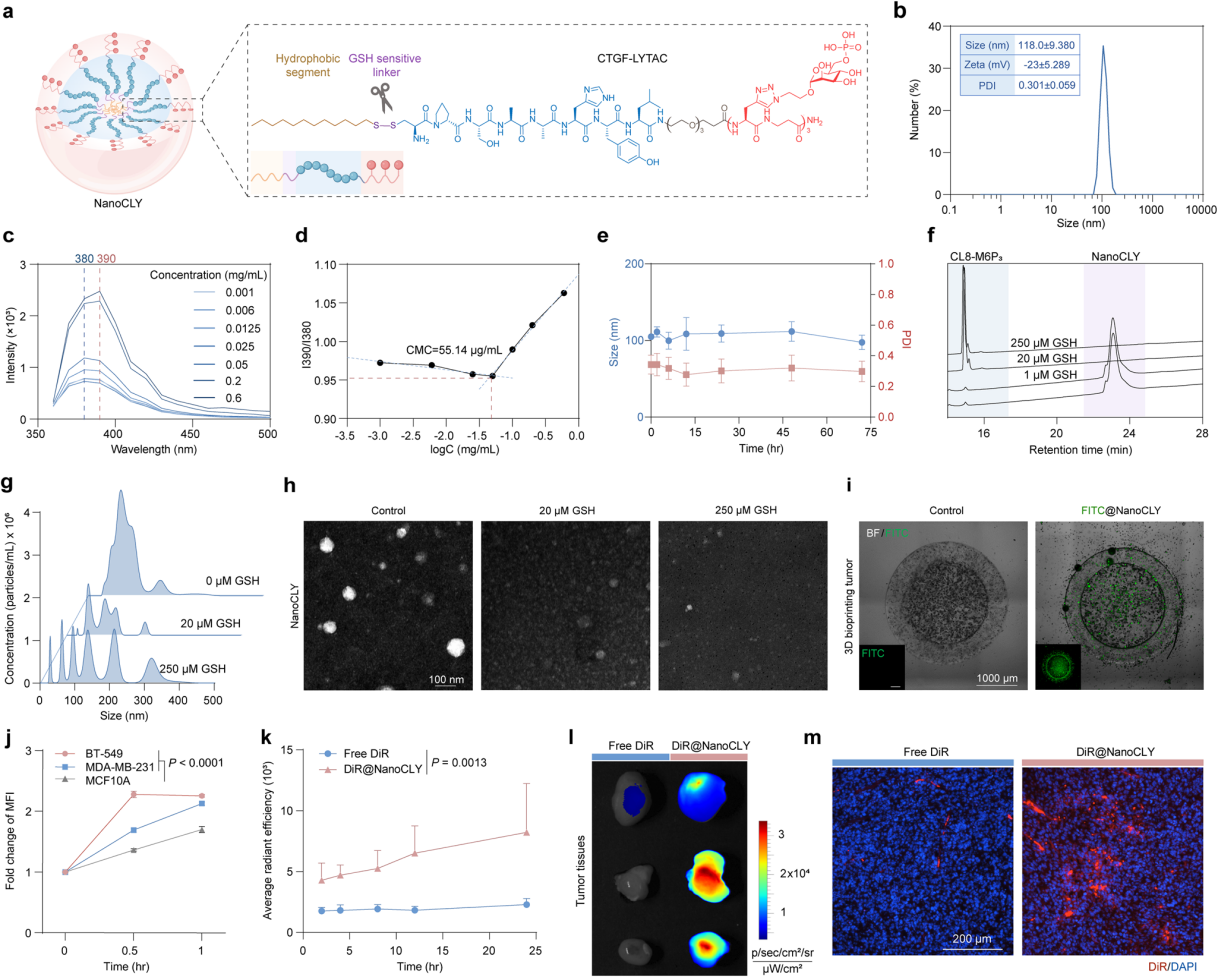
Design, construction, and characterization of NanoCLY. a) Schematic illustration of the NanoCLY construction. b) Size, polymer dispersity index (PDI), and zeta potential of NanoCLY. c,d) Critical micelle concentration (CMC) analysis of NanoCLY. e) Size and PDI of NanoCLY in PBS, 37 °C for 72 h (*n* = 3). f) HPLC traces of NanoCLY treated with 0, 1 × 10^−6^, 2 × 10^−5^, 2.5 × 10^−4 ^_M_ GSH. g,h) Particle sizes, and typical transmission electron microscope images of NanoCLY treated with 0, 2 × 10^−5^, 2.5 × 10^−4 ^_M_ GSH. i) Representative images of FITC@NanoCLY penetration in BT549/CAF 3D bioprinting tumor models. j) Quantification of cellular uptake of FITC@NanoCLY in BT549, MDA‐MB‐231, and MCF10A cells, measured by FCM (*n* = 3). The P values were calculated by two‐way ANOVA. k–m) Time course analysis of tumor fluorescence intensity, tumor tissues, and immunofluorescence analysis at tumor tissues after post‐injection of DiR@NanoCLY (DiR at 0.5 mg kg^−1^) for 24 h (*n* = 3). For (e, j, k), the quantified data from different experiments were presented as the mean ± SD.

To address the potential drawbacks of CTGF‐LYTAC based on peptide ligands, such as short plasma half‐life, instability, and unfavorable systemic profiles,^[^ ^32^, ^33^, ^34^ ^]^ we developed a CTGF‐LYTAC nano‐system aimed at enhancing tumor‐selective CTGF degradation in vivo. This system involves the conjugation of a hydrophilic lauryl group with hydrophilic CL8‐M6P_3_ using a GSH‐responsive disulfide bond linker to create an amphiphilic chimera (Figure S9, Supporting Information). This amphiphilic nature enables the chimera to self‐assemble into nanoparticles in aqueous media, termed NanoCLY (**Figure** **5**a). Circular dichroism (CD) spectroscopy showed that neither CL8‐M6P_3_ nor NanoCLY altered the secondary structure of CL8 (Figure S10, Supporting Information). Furthermore, all components, CL8, CL8‐M6P_3_, and NanoCLY, exhibited good hemolysis safety (Figure S11, Supporting Information). Dynamic light scattering (DLS) analysis revealed that NanoCLY had a hydrodynamic diameter of 118.0  ±  9.38 nm (polymer dispersity index, PDI, 0.301  ±  0.059), and a zeta potential of −23.0 ±  5.289 mV (Figure 5b). To assess micelle formation, we performed critical micelle concentration (CMC) analysis using pyrene as a fluorescence dye, identifying a CMC of 55.14 × 10^−6^ g mL^−1^ (Figure 5c,d). NanoCLY demonstrated relative stability in PBS (pH 7.4) at 37 °C for at least 72 h (Figure 5e). Given that GSH concentrations in tumor cells (0.2 × 10⁻^2^ to 1 × 10⁻^2^ m) are markedly higher than those in plasma (≈2 × 10⁻⁶ m),^[^ ^35^, ^36^ ^]^ we quantified GSH levels in our TNBC models to evaluate this disparity. GSH concentrations were (18.13±0.12) × 10^−6^ m in TNBC cell supernatant, (250.24±10.65) × 10^−6^ m in the tumor extracellular matrix, and (1.51±0.02) × 10^−6^ M in plasma (Figure S12a, Supporting Information). Analytical HPLC (214 nm) demonstrated selective cleavage of the disulfide linker at TME‐relevant GSH concentrations (2 × 10⁻⁵ and 2.5 × 10⁻⁴ m), while it remained stable under plasma‐mimicking GSH levels (1.5 × 10⁻⁶ m) (Figure 5f). Further characterization using Nanosight and transmission electron microscopy (TEM) revealed that NanoCLY underwent micelle dissociation upon exposure to GSH at TME‐mimicking concentrations (Figure 5g,h). The in vivo GSH‐responsiveness of NanoCLY was further validated using H₂O₂‐induced GSH depletion as a control. Analytical HPLC confirmed the selective cleavage of NanoCLY at the tumor site, demonstrating its tumor‐specific responsiveness (Figure S12b, Supporting Information). In contrast, NanoCLY retained its size and structural integrity under plasma conditions (Figure S12c–e, Supporting Information). To evaluate the therapeutic universality and targeting efficiency of NanoCLY, we analyzed CI‐M6PR expression, the key receptor mediating LYTAC function, across various breast cancer subtypes. Bioinformatics analysis revealed a significantly higher expression of CI‐M6PR in breast cancer tissues, particularly in the TNBC and HER2‐positive subtypes (Figure S13a, Supporting Information). Western blotting and ELISA further confirmed that CTGF degradation efficacy mediated by NanoCLY was positively correlated with CI‐M6PR expression (Figure S13b,c, Supporting Information). Notably, CI‐M6PR knockdown significantly impaired CTGF degradation, indicating that NanoCLY activity is dependent on CI‐M6PR (Figure S13d, Supporting Information). Collectively, these results demonstrated that NanoCLY is a stable, tumor‐targeted, and GSH‐responsive nanoplatform with strong potential as a universal strategy for anti‐TNBC therapy.

Moreover, NanoCLY presents the M6P ligand on its surface, facilitating tumor‐targeted delivery due to the high expression of CI‐M6PR at TNBC tumor sites. To evaluate spatial delivery within a 3D bioprinting TNBC tumor model, we prepared FITC@NanoCLY and found that it penetrated throughout the entire spheroid after 4 h (Figure 5i, Figure S14a–d, Supporting Information). We next assessed the TNBC‐targeting ability of NanoCLY in vitro, by incubating BT549, MDA‐MB‐231, and nonmalignant mammary epithelial cells (MCF10A) with FITC@NanoCLY for 0.5 and 1 h. FCM results indicated that significantly stronger cellular uptake of FITC@NanoCLY in BT549 and MDA‐MB‐231 cells compared to MCF10A cells, attributed to the relatively higher expression of CI‐M6PR in TNBC cells (Figure 5j, and Figure S14e,f, Supporting Information). Furthermore, we evaluated the biodistribution and tumor targeting of NanoCLY in orthotopic MDA‐MB‐231 tumor‐bearing mice. For in vivo imaging, DiR was used as the near‐infrared fluorescent probe. Compared to free DiR, DiR@NanoCLY exhibited stronger fluorescent signals at tumor sites across the examined time points (Figure 5k,l, and Figure S15a,c, Supporting Information). Moreover, NanoCLY showed reduced DiR accumulation in the lungs (Figure S15b,c, Supporting Information). Consistent with the NanoCLY penetration observed in 3D bioprinted tumor models, significantly stronger fluorescent signals were detected at tumor sites (Figure 5m). Overall, DiR@NanoCLY demonstrated rapid accumulation at tumor sites and prolonged retention time after systemic administration.

### 2.7 NanoCLY Mediates CTGF Degradation and Exhibits Antitumor Effects in vitro

**Figure 6:**
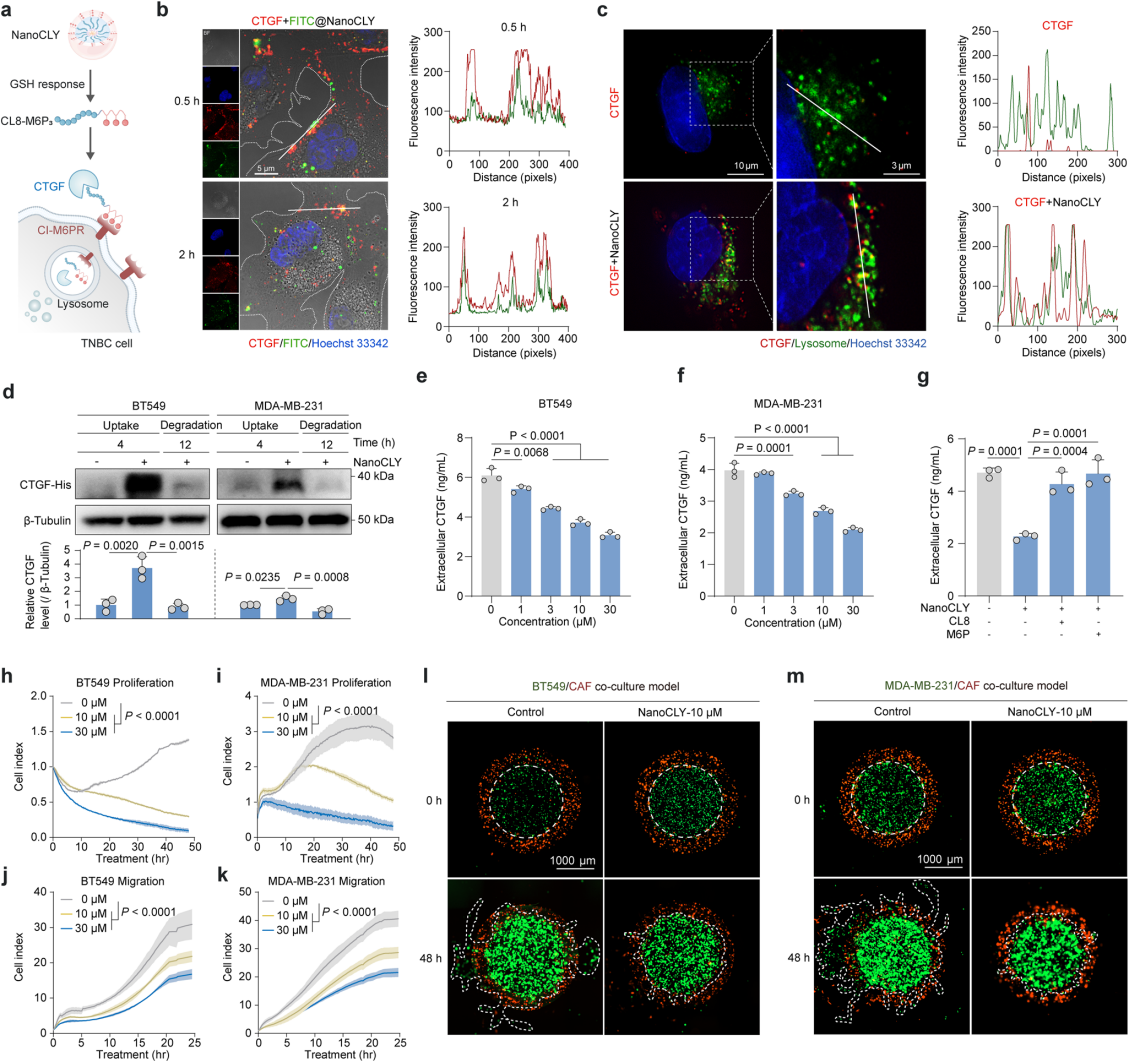
NanoCLY mediates CTGF degradation and exhibits anti‐TNBC efficacy in vitro. a) A schematic diagram showing the TME‐responsive CTGF degradation by NanoCLY. b) Confocal microscopy images of BT549 cells treated with CTGF and FITC@NanoCLY for 0.5 and 2 h, along with colocalization analysis of CTGF and FITC@NanoCLY. c) Confocal microscopy images of BT549 cells treated with CTGF and NanoCLY for 4 h, labeled lysosomes with LysoTracker Green, and colocalization analysis of CTGF and lysosomes. d) The effects of NanoCLY on CTGF protein uptake and decrease in BT549 and MDA‐MB‐231 cells (*n* = 3). The *P* values were calculated by one‐way ANOVA. e, f) ELISA of CTGF protein levels in culture media of BT549 and MDA‐MB‐231 cells incubated with NanoCLY for 12 h (*n* = 3). The *P* values were calculated by one‐way ANOVA. g) ELISA analysis of CTGF protein levels in the culture media of BT549 cells incubated with NanoCLY, with or without 2 × 10^−4 ^_M_ CL8 or M6P (*n* = 3). The *P* values were calculated by one‐way ANOVA. Kinetic curves of the h,i) proliferation and j,k) migration of BT549 and MDA‐MB‐231 cells after NanoCLY treatment, as assessed by xCELLigence RTCA‐DP (*n* = 3). The P values were calculated by two‐way ANOVA. l,m) Immunofluorescence images of BT549/CAF and MDA‐MB‐231/CAF co‐culture 3D bioprinting models after NanoCLY treatment. For (d‐k), the quantified data from different experiments were presented as the mean ± SD.

To evaluate whether NanoCLY could induce CTGF cellular endocytosis in TNBC cells (**Figure** **6**a), BT549 cells were co‐incubated with FITC@NanoCLY and fluorescent CTGF, and then observed using confocal microscopy. As shown in Figure 6b, FITC@NanoCLY exhibited significant colocalization with CTGF at the cell membrane within 30 min, with these signals internalized into the cytoplasm by 2 h. To further confirm the intracellular localization of CTGF, we examined its colocalization with lysosomes in BT549 cells at 4 h, comparing it to CTGF alone treatment. The results suggested the involvement of lysosomes in NanoCLY‐mediated endocytosis (Figure 6c). To assess whether NanoCLY mediates CTGF lysosomal degradation, we quantified CTGF protein levels using western blotting assay and ELISA. The results demonstrated that CTGF uptake mediated by NanoCLY occurred within 4 hours, with subsequent degradation effects extending up to 12 h (Figure 6d). Moreover, extracellular CTGF levels were significantly reduced by NanoCLY in a dose‐dependent manner, similar to the effects observed with CL8‐M6P_3_ (Figure 6e,f). Importantly, coincubation with exogenous ligands (M6P and CL8) effectively blocked CTGF degradation, confirming that the process depends on the formation of a ternary complex involving CTGF, CL8‐M6P_3_, and CI‐M6PR (Figure 6g). Quantitative analysis revealed that ≈50% of extracellular CTGF was degraded by either CL8‐M6P_3_ or NanoCLY in vitro, aligning with the degradation efficiency reported for genetically encodable LYTACs based on nanobody or scFv warheads.^[^ ^37^ ^]^ This moderate degradation may be associated with i) the relatively low binding affinity between warheads (e.g., peptides, nanobody, and scFc) and their targeted proteins and ii) competition for CI‐M6PR by native M6P‐tagged lysosomal hydrolases.^[^ ^38^ ^]^ Strategies that involve covalent binding between warheads and targets have been developed to enhance specificity and efficiency in protein degradation.^[^ ^39^, ^40^ ^]^ Moreover, EndoTags have been designed to bind to an orthogonal site distinct from M6P, thereby reducing competition with native ligands.^[^ ^22^ ^]^ Next, consistent with CL8‐M6P_3_, the cell growth and migration curve from RTCA revealed that NanoCLY significantly inhibited cell proliferation and migration in BT549 and MDA‐MB‐231 cells (Figure 6h‐k). Both mono‐TNBC transwell assay (Figure S16, Supporting Information) and TNBC/CAF co‐culture models (Figure 6l,m) confirmed that NanoCLY significantly inhibited TNBC cell migration. These findings collectively confirmed that NanoCLY promoted CTGF degradation through the endo‐lysosomal pathway, thereby inhibiting TNBC proliferation and migration in vitro.

### 2.8 Anti‐TNBC and Anti‐Metastasis Efficacy of NanoCLY in vivo

**Figure 7:**
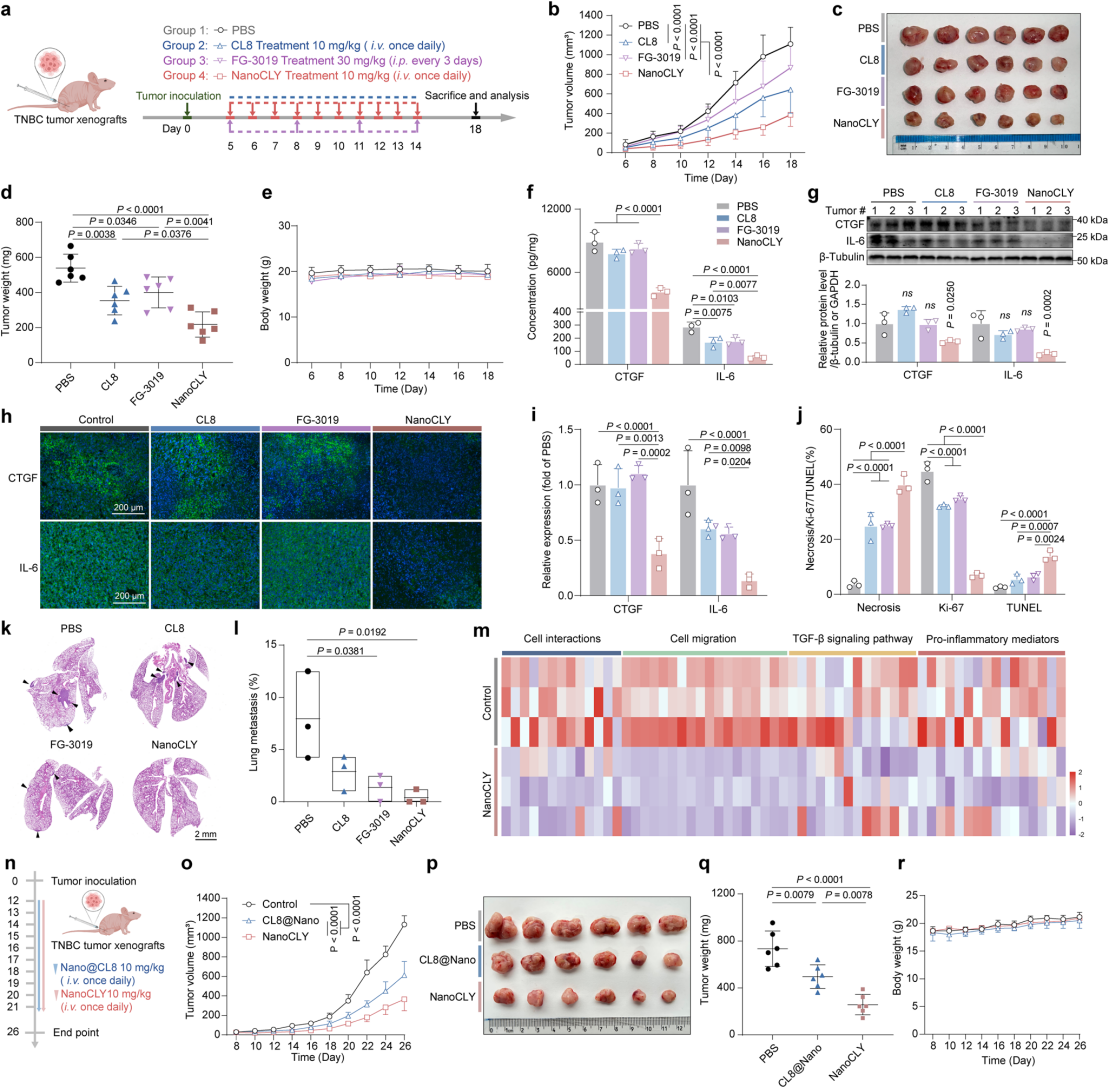
Inhibition of tumor growth and lung metastasis by NanoCLY in vivo. a) Flow diagram of the treatment regimen in the MDA‐MB‐231 tumor model. b) Tumor growth during treatments (*n* = 6). The *P* values were calculated by two‐way ANOVA. c) Photographs of tumors excised post‐treatment (*n* = 6). d) Weight of excised tumors (n = 6). The P values were calculated by one‐way ANOVA. e) Body weight during treatments (*n* = 6). f) ELISA and g) Western blotting assay of CTGF and IL‐6 protein level of tumors (*n* = 3). The *P* values were calculated by one‐way ANOVA. h,i) Immunofluorescence staining and quantitative analysis of CTGF and IL‐6 protein level in tumor samples (*n* = 3). j) Image‐based quantitative results of H&E, Ki‐67, and TUNEL staining of tumor samples (*n* = 3). The *P* values were calculated by one‐way ANOVA. k,l) H&E staining of lung tissues and quantitative analysis of metastatic lesions, indicated by arrows (*n* = 3). The *P* values were calculated by one‐way ANOVA. m) Heatmap images showing a significant reduction in the levels of genes related to cell communications, metastasis, the TGF‐β signaling pathway, and pro‐inflammatory in the NanoCLY‐treated group (*n* = 3). n) Flow diagram of the treatment regimen. o) Tumor growth during treatments (*n* = 6). The *P* values were calculated by two‐way ANOVA. p) Photographs of tumors excised post‐treatment (*n* = 6). q) Weight of excised tumors (*n* = 6). The *P* values were calculated by one‐way ANOVA. r) Body weight during treatments (*n* = 6). For (b,d–g,i,j,l,o,q,r), the quantified data from different experiments were presented as the mean ± SD.

Encouraged by the aforementioned findings, we investigated the tumor‐suppressive capabilities of NanoCLY. To evaluate the in vivo anti‐TNBC effects of NanoCLY, MDA‐MB‐231 tumor‐bearing mice were administered with NanoCLY (10 mg kg^−1^) or CL8 (10 mg kg^−1^) daily via intravenous (i.v.) injection, with FG‐3019 serving as a positive control. The treatment schedules are depicted in **Figure** **7**a. Compared to the PBS, CL8, and FG‐3019 treatment groups, NanoCLY treatment induced significant tumor regression (Figure 7b) and reduced tumor weight (Figure 7c,d), resulted in a substantial tumor growth inhibition (TGI) of 59.62%. Importantly, none of the treatment groups caused significant changes in body weight (Figure 7e) or induced notable histological alterations in major organs, as observed from H&E staining (Figure S17, Supporting Information) and blood analysis (Figure S18, Supporting Information). To assess whether NanoCLY could induce CTGF protein degradation in vivo, we evaluated CTGF levels and the feedback loop signaling protein IL‐6 in tumor samples using ELISA and western blotting assays. The results indicated that CTGF and IL‐6 protein levels were significantly lower in the NanoCLY treatment group compared to the control, CL8, and FG‐3019 treatment groups (Figure 7f,g). Similarly, IF analyses of tumor sections revealed reduced levels of CTGF and IL‐6 in the NanoCLY‐treated group (Figure 7h,i). Collectively, these results indicated that NanoCLY significantly blocked CTGF‐IL‐6 cell crosstalk in vivo. Pathological and immunohistochemical examinations of tumor samples showed that NanoCLY treatment resulted in pronounced necrotic lesions and increased TUNEL‐positive cells, alongside a decrease in Ki‐67 positive cells (Figure 7j, Figure S19, Supporting Information). These observations suggested that NanoCLY‐mediated CTGF degradation promoted its antitumor effects in vivo. Compared to the in vivo anti‐TNBC therapeutic effect of selectively knocking down CTGF expression in tumor cells, NanoCLY resulted in a significant TGI of ≈60%. This difference may be attributed to the multicellular expression of CTGF within the TME, highlighting the advantages of a TME‐CTGF degradation strategy.

Furthermore, reduced metastasis was observed at the lung tissues of the NanoCLY‐treated group, indicating that CTGF degradation mediated by NanoCLY may inhibit TNBC metastasis (Figure 7k,l). Metastasis is a major contributor to the high mortality rate associated with TNBC, with the lung being the second most common site of breast cancer metastasis; the 5‐year overall survival rate is only 16.8%.^[^ ^41^ ^]^ To assess the anti‐metastasis effects of NanoCLY, a lung metastasis model was established by i.v. injection of 5 × 10^5^ MDA‐MB‐231‐Luc cells into mice. Metastatic tumor progression was monitored by noninvasive bioluminescence imaging on day 12, 18, and 24. As shown in Figure S20a–d, Supporting Information, NanoCLY at 10 mg kg^−1^ significantly reduced lung colonization by MDA‐MB‐231 cells. Similar results were obtained from histological analysis of lung samples, which demonstrated a reduction of lung metastasis area after NanoCLY treatment (Figure S20e, Supporting Information). To further elucidate the multiple molecular mechanisms of CTGF degradation by NanoCLY in the TME, transcriptomic analysis was conducted to identify enriched signaling pathways in tumor tissues. The enrichment analyses revealed a series of downregulated signaling pathways in NanoCLY treatment group, including those involved in cell interactions, cell migration, the TGF‐β signaling pathway, and pro‐inflammatory mediators (Figure 7m). These findings indicated that NanoCLY effectively suppressed CTGF‐mediated TNBC progression and metastasis by regulating multiple factors related to cell‐cell interactions, pro‐tumor signaling pathways, and the inflammatory microenvironment in vivo. In comparison to conventional CTGF blockade therapy, NanoCLY demonstrated superior tumor‐targeted delivery and protein degradation capabilities, leading to enhanced anti‐tumor efficacy. To further assess the degradation efficiency of NanoCLY over CL8, we conducted an in vivo experiment using CL8@Nano as the control (Figure S21, Supporting Information). The results showed that NanoCLY exhibited a significantly stronger anti‐tumor effect than CL8@Nano (Figure 7n‐r), highlighting the degradation and therapeutic advantages conferred by the M6P modification in the NanoCLY system.

### 2.9 Synergistic Anti‐TNBC Effects of Combination Therapy with NanoCLY and Paclitaxel

**Figure 8:**
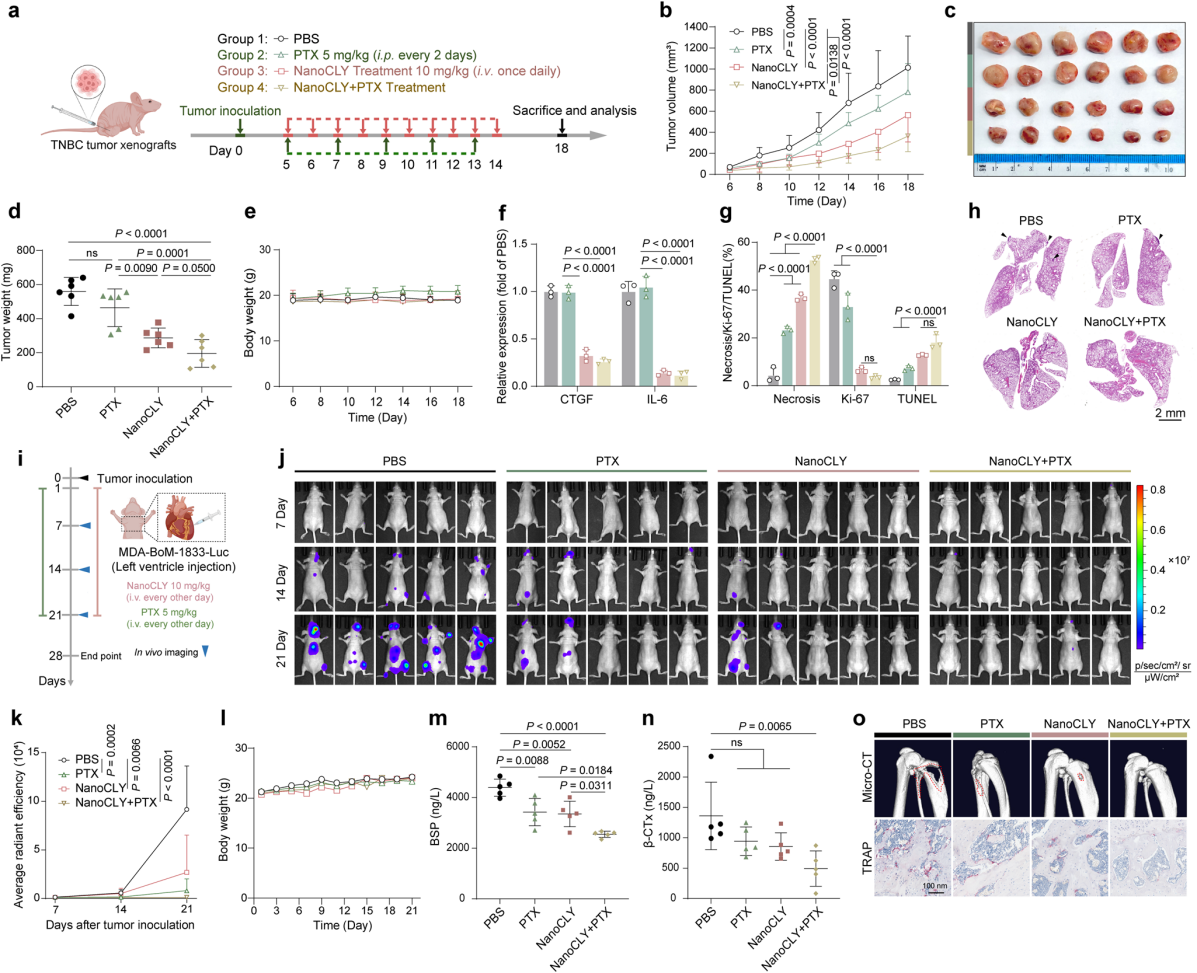
Inhibition of tumor growth and metastasis by combination therapy of PTX with NanoCLY in vivo. a) Flow diagram of a combination treatment regimen in the MDA‐MB‐231 tumor model. b) Tumor growth during treatments (*n* = 6). The *P* values were calculated by two‐way ANOVA. c) Photographs of tumors excised post‐treatment (*n* = 6). d) Weight of excised tumors (*n* = 6). The *P* values were calculated by one‐way ANOVA. e) Body weight during treatments (*n* = 6). f) Immunofluorescence staining and quantitative analysis of CTGF and IL‐6 protein level in tumors (*n* = 3). g) Image‐based quantitative results of H&E, Ki‐67, and TUNEL staining of tumor samples (*n* = 3). The *P* values were calculated by one‐way ANOVA. h) H&E staining lungs and quantitative analysis of different groups. The arrow marks the metastatic lesions (n = 3). The *P* values were calculated by one‐way ANOVA. i) FCM diagram of the treatment regimen in bone metastatic breast cancer model. j) In vivo imaging of mice every week (*n* = 5). k) Quantification of the fluorescence intensity every week (*n* = 5). The *P* values were calculated by two‐way ANOVA. l) Body weight of mice during treatments (*n* = 5). m) BSP and n) 𝛽‐CTx levels in mouse blood serum detected through ELISA (*n* = 5). The P values were calculated by one‐way ANOVA. o) Micro‐CT and histological TRAP images of bone lesions from representative mice. For (b,d–g, k–n), the quantified data from different experiments were presented as the mean ± SD.

Building on the in vivo anti‐TNBC therapeutic outcomes from both CTGF knockdown and CTGF degradation strategies, we found that both approaches resulted in a tumor inhibition rate of ≈50%. Notably, clinical trials have shown that CTGF blockade is generally ineffective as a monotherapy but demonstrates enhanced efficacy when combined with chemotherapeutic agents (Clinical trial No.: NCT02210559, NCT01181245, and NCT03941093).^[^ ^14^ ^]^ CTGF has been implicated in the activation of iCAFs, which contribute to the tumor‐promoting microenvironment, further exacerbating drug resistance.^[^ ^42^ ^]^ In our study, CTGF significantly enhanced iCAF activation, whereas CL8‐M6P_3_ effectively inhibits this activation, reshaping the inflammatory tumor microenvironment and showing potential in combating drug resistance. Furthermore, CTGF blockade has been associated with a decrease in key promoters responsible for chemotherapy resistance.^[^ ^43^ ^]^ Given the widespread clinical use of paclitaxel (PTX) in treating TNBC,^[^ ^44^ ^]^ a chemotherapeutic agent often hindered by intrinsic and acquired resistance,^[^ ^45^ ^]^ we investigated whether combining CTGF degradation with PTX could synergistically enhance therapeutic outcomes for TNBC. The results indicated that NanoCLY potentiated the antitumor effect of PTX in vivo (**Figure** **8**a–d). Consistent with Figure 7h, IF analyses of tumor sections revealed lower levels of CTGF and IL‐6 in both the NanoCLY and combination treatment groups (Figure 8f, Figure S22, Supporting Information). Pathological and immunohistochemical examinations revealed that the combination of NanoCLY with PTX reduced tumor cell proliferation, increased necrosis area, and promoted apoptosis, exhibiting synergistic antitumor effects in vivo (Figure 8g, Figure S23, Supporting Information). In addition, there was no measurable toxicity in the mice receiving the combined treatment, as evidenced by stable body weight (Figure 8e) and no abnormalities in the histology of major organs or blood analysis results (Figures S24 and S25, Supporting Information). As shown in Figure 8h and Figure S26, Supporting Information, the least metastasis was found in the lung tissues of the NanoCLY+PTX‐treated group.

Bone metastasis accounts for ≈75% of metastatic cases, with a 5‐year overall survival rate is just 22.8%.^[^ ^46^ ^]^ Overexpression of CTGF has been linked to enhanced bone metastatic activity in breast cancer cells.^[^ ^11^ ^]^ To evaluate the anti‐bone metastasis effects of CTGF degradation and its potential synergy with chemotherapeutics, a bone metastatic breast cancer model was established. Bone metastatic tumor progression was monitored by in vivo bioluminescence imaging on the 7^th^, 14^th^, and 21^st^ day (Figure 8i). As shown in Figure 8j,k, significant bone metastasis was observed in the control group after 21 days. Both NanoCLY and PTX treatment groups exhibited moderate inhibitory effects on bone metastasis, while the combination therapy displayed the strongest anti‐metastasis effect. None of the treatment groups affected the body weight of the mice, demonstrating their safety (Figure 8l). Metastatic tumor cells can secrete factors that activate osteoclasts in the bone marrow, causing osteolysis and promoting tumor progression.^[^ ^47^ ^]^ Here, two biomarkers of osteoclasts, bone sialoprotein (BSP) and beta C‐terminal cross‐linked telopeptides of type I collagen (𝛽‐CTx), were detected by ELISA. The results revealed that the combination of NanoCLY and PTX minimized levels of BSP and 𝛽‐CTx, indicating reduced osteoclast formation in the bone marrow (Figure 8m,n). Computed tomography (Micro‐CT) analysis demonstrated that osteolysis of the hind limbs was moderately reduced in the PTX and NanoCLY treatment groups, with a significantly reduction in the combined treatment group (Figure 8o). Histological analysis also revealed a maximally decreased tartrate‐resistant acid phosphatase (TRAP)^+^‐osteoclasts in mouse hind limbs in NanoCLY+PTX‐treated group (Figure 8o and Figure S27, Supporting Information). Therefore, the combination of CTGF degradation by NanoCLY with PTX effectively suppressed the growth of MDA‐MB‐231 orthotopic tumors and reduced both lung and bone metastasis in vivo.

Taken together, we explored the potential of CTGF as a TNBC therapeutic target by the Nano‐LYTAC strategy. TNBC represents the most aggressive subtype, notorious for its high recurrence and mortality rates.^[^ ^48^ ^]^ Notably, compared to primary tumors, both lung metastasis^[^ ^49^ ^]^ and bone metastasis of TNBC^[^ ^11^ ^]^ exhibit elevated CTGF expression. Beyond the antiproliferative effects of CTGF‐LYTAC, we were surprised to discover that CTGF degradation can significantly reduce TNBC metastasis in various models. Through omics approaches including transcriptomics, proteomics, and cytokine array, we found that CTGF‐LYTAC significantly regulated the TNBC TME by downregulating the TGF‐β signaling pathway and disrupting TNBC‐CAF communications. Cumulative studies have highlighted the critical role of CAFs in promoting tumor metastasis through paracrine communications. Our findings revealed a CTGF/IL‐6 loop between TNBC and CAFs, where CTGF secreted by TNBC cells activated iCAFs, which in turn secreted IL‐6, further enhancing CTGF expression in TNBC cells. As a key biomarker of iCAFs, IL‐6 plays a crucial role in tumor‐promoting inflammation, the EMT process, and metastasis.^[^ ^50^, ^51^, ^52^ ^]^ Rather than depleting CAFs, CTGF‐LYTAC blocked the CTGF/IL‐6 feedback loop in the TME by degrading CTGF, thereby inhibiting iCAF activation and offering a potent and effective approach for TME‐directed anti‐TNBC therapies. Additionally, NanoCLY demonstrated significant protein degradation efficacy in TNBC, correlating with CI‐M6PR expression levels. Given the relatively high expression of CI‐M6PR in TNBC tissues, we developed NanoCLY to achieve potent protein degradation. To address diseases with low CI‐M6PR expression, a variety of emerging approaches targeting alternative internalizing receptors have been developed, including asialoglycoprotein receptor‐targeted LYTACs,^[^ ^25^ ^]^ cytokine receptor‐targeting chimeras (KineTACs),^[^ ^53^ ^]^ integrin‐facilitated lysosomal degradation (IFLD),^[^ ^54^ ^]^ autophagy‐inducing antibodies (AUTABs),^[^ ^55^ ^]^ signal‐mediated LYTACs (SignalTACs),^[^ ^56^ ^]^ and recycling transferrin receptor‐mediated LYTACs (Pep‐TACs).^[^ ^57^ ^]^ These strategies could be adapted for tumors with low CI‐M6PR expression, broadening the applicability of targeted protein degradation platforms.

## 3 Conclusion

In this study, we observed notable overexpression of CTGF by both TNBC cells and CAFs within the TME, correlating closely with TNBC initiation and prognosis. CTGF, a multifunctional signaling protein with four structural domains, significantly influences cell proliferation, migration, and intercellular interactions within the TME. Consistent with previous findings,^[^ ^9^ ^]^ our results demonstrate that knockdown of full‐length CTGF markedly reduced TNBC cell proliferation and metastasis in vitro and in vivo, underscoring CTGF as a promising therapeutic target for TNBC. Traditional CTGF‐targeting approaches, however, have faced considerable challenges due to the absence of a crystal structure for CTGF and the limitations of current occupation‐driven therapeutics, which may inadequately inhibit full‐length CTGF activity. Our study introduces CL8‐M6P_3_, the first CTGF‐LYTAC, which effectively degrades full‐length CTGF protein, significantly suppressing TNBC cell proliferation and migration. Despite this advancement, the physicochemical properties and limited tumor‐targeting efficiency of LYTACs necessitate further development for clinical translation. To address these challenges, we engineer NanoCLY, a CTGF‐LYTAC nanoplatform with enhanced tumor‐targeting and lysosomal degradation capabilities within the TME. NanoCLY shows substantial efficacy in inhibiting TNBC growth and metastasis both in vitro and in vivo by disrupting TNBC/CAF signaling and cellular crosstalk. The anti‐TNBC efficacy of LYTAC‐mediated CTGF degradation therapy exceeds that of antibody‐based blockade therapy. Moreover, NanoCLY exhibits a synergistic effect when combined with chemotherapy, underscoring its potential as a complementary strategy for TNBC treatment.

In summary, we developed the first LYTAC nanoplatform for CTGF degradation, featuring tumor‐targeted delivery and robust anti‐TNBC activity alongside a synergistic effect with paclitaxel. This LYTAC approach offers a powerful tool for oncology research, establishing a solid foundation for future therapeutic exploration and drug discovery efforts targeting CTGF.

## 4 Experimental Section

### Materials and Reagents

Rink Amide MBHA resin (0.45 mmol g^−1^ loading) was purchased from Tianjin Nankai Hecheng Science & Technology Co., Ltd.; All the other chemical reagents and solvents used were purchased from Adamas‐beta, CSBio (Shanghai), Energy Chemical, MedChemExpress, and Sinopharm Chemical Reagent Co., Ltd.

### Cell Culture, and Animal Models

The BT549, MDA‐MB‐231, Hs578T, and MCF10A cell lines were obtained from the Cell Bank of the Chinese Academy of Sciences (Shanghai, China), among which BT549, MDA‐MB‐231 were labeled with EGFP and luciferase tag in the lab. The MDA‐BoM‐1833 cell line was provided by Professor Yu‐Dong Zhou from the University of Mississippi and labeled with a luciferase tag in the lab. Human breast cancer‐associated fibroblast was obtained from YaJi Biological (Shanghai, China) and labeled with RFP in the lab. BT549 cell was maintained in Roswell Park Memorial Institute (RPMI) 1640 medium supplemented with 10% FBS and 1% penicillin & streptomycin in a 5% CO_2_ incubator at 37 °C. MDA‐MB‐231 cell was cultured in Leibovitz's L‐15 medium supplemented with 10% FBS and 1% penicillin & streptomycin in a non‐CO_2_ incubator at 37 °C. Hs578T and MDA‐BoM‐1833 cells were cultured in Dulbecco's Modified Eagle Medium (DMEM) medium supplemented with 10% FBS and 1% penicillin and streptomycin in a 5% CO_2_ incubator at 37 °C. MCF10A cells were cultured in a specific medium (ZQ‐1311, Zhongqiao Xinzhou Biotechnology) supplemented with 10% FBS and 1% penicillin and streptomycin in a 5% CO_2_ incubator at 37 °C. Breast cancer‐associated fibroblast was cultured in a specific medium (0091a‐003, YaJi Biological) supplemented with 10% FBS and 1% penicillin & streptomycin in a 5% CO_2_ incubator at 37 °C.

Female BALB/c nude mice (4–6 weeks old, 18–20 g) were provided by the Shanghai Model Organisms Center, Inc., and housed under specific pathogen‐free conditions for use in orthotopic xenograft tumor model experiments, lung metastasis tumor model experiments, and bone metastasis tumor model experiments. All animal procedures were carried out under the guidelines approved by the Institutional Animal Care and Use Committee (IACUC) of Shanghai Model Organisms Center Inc. The approval number for animal experiments is 2024‐0011.

### Plasmids and siRNAs

Control shRNA and CTGF‐specific shRNA were made from Genomeditech (Shanghai, China) by inserting the sequences listed in Table S1 (Supporting Information) into the PGMLV‐HU6‐MCS‐CMV‐ZsGreen1‐PGK‐puro viral vector. shRNAs were made by annealing and inserting oligonucleotides into the BamHI and EcoRI sites of the viral vector. Scrambled sequences, confirmed to have no interference with human proteins, were used to generate control shRNA. CTGF knockdown cells were established by CTGF‐specific shRNA and the stable transfected cell lines were then screened by puromycin. Control siRNA and CTGF and CI‐M6PR‐specific siRNAs were purchased from GenePharma (Shanghai, China), and the siRNA sequences were listed in Table S2 (Supporting Information). Transfection reagent GP‐transfect‐mate (G04008, GenePharma) was used according to the manufacturer's protocol. To transfect cells on a 6‐well plate with siRNAs, 150 pmol siRNA was mixed with 6 µL GP‐transfect‐mate, incubated at room temperature for 15 min, and then added to the cells.

### Real‐Time Cell Analysis (RTCA)

The xCELLigence RTCA systems (Agilent, USA), based on electrical impedance, allow real‐time and label‐free measurement of cell proliferation and migration. The obtained cell index reflects a comprehensive characterization including cell adhesion and spreading. The background signal was detected with a cell culture medium/well. For proliferation assays done with expression vector/siRNA transfected cells, 5 × 10^3^ BT549 and MDA‐MB‐231 cells per well were seeded on E‐Plate 16 at 24 h post‐transfection. For proliferation assays done with reagent‐treated cells, 5 × 10^3^ BT549 and MDA‐MB‐231 cells per well were seeded on E‐Plate 16 and incubated overnight. The cells were treated with CL8‐M6P_3_ or NanoCLY at various concentrations (0, 1 × 10^−5^, 3 × 10^−5^ m). Then cell index values were calculated using the RTCA software.

For migration assay done with expression vector/siRNA transfected cells, 1.5 × 10^4^ BT549 and MDA‐MB‐231 cells per 100 µL basic medium were seeded on the upper chamber of CIM‐Plate 16 at 24 h post‐transfection. The lower chamber was added 160 µL growth medium with 10% FBS. Then cell index values were calculated for 24 h. For migration assay done with reagent‐treated cells, 1.5 × 10^4^ BT549 and MDA‐MB‐231 cells per 100 µL basic medium were seeded on the upper chamber of CIM‐Plate and treated with CL8‐M6P_3_ or NanoCLY at various concentrations for 24 h. Then cell index values were calculated using the RTCA software.

### Transwell Migration Assay

Cell migration assay was performed using 8 µm pore size transwell chambers (3422, Corning). For transwell assay done with expression vector/siRNA transfected cells, 2 × 10^4^ BT549 and MDA‐MB‐231 cells per 200 µL basic medium were seeded in the upper chamber for 16 h at 24 h post‐transfection. For transwell migration assay done with reagent‐treated cells, 2 × 10^4^ BT549 and MDA‐MB‐231 cells per 200 µL basic medium were seeded on an upper chamber treated with CL8‐M6P_3_ (0, 3 × 10^−6^, 10 × 10^−6^_,_ 30 × 10^−6^ m), FG‐3019 (100 µg mL^−1^) or NanoCLY (0, 10 × 10^−6^, 30 × 10^−6 ^_M_) for 12 h. 2× 10^4^ CTGF knockdown BT549 and MDA‐MB‐231 cells treated with 5 × 10^−8^ M CTGF were seeded on the upper chamber in the presence or absence of 5 × 10^−6 ^M CL8‐M6P_3_ for 16 h. The lower chamber was added with 500 µL normal growth medium supplemented with 10% FBS. Cells fixed with 4% paraformaldehyde and stained by crystal‐violet dye for 30 min on the bottom surface of the polycarbonate membranes were observed under a microscope for analysis. The intensity of the migrated cells was measured using ImageJ software.

### Colony Formation Assay

The colony formation assay was used to evaluate the proliferative ability of BT549 and MDA‐MB‐231 cells. BT549 and MDA‐MB‐231 cells at 24 h post‐transfection were seeded at 2 × 10^3^ cells per well in 12‐well plates. After 7 days, cells were fixed with 4% paraformaldehyde and stained with 0.5% crystal‐violet dye for 30 min. Images were obtained with Cytation5 (Biotek, USA).

### 3D‐Bioprinting TNBC‐CAF Co‐culture Model

For 3D co‐culture, the cell suspension for the model core consisted of 1 × 10^6^ cells mL^−1^ TNBC cells, including shControl, shCTGF BT549 cells labeled with EGFP, and MDA‐MB‐231 cells labeled with EGFP. The cell suspension solution for the peripheral region consisted of 1 × 10^6^ cells mL^−1^ CAFs labeled with RFP. All cell suspensions were aliquoted into tubes and stored on ice before use. The prepolymer solution for bioprinting was prepared with 16% (w/v) GelMA and 0.4% (w/v) lithium phenyl(2,4,6‐trimethylbenzoyl) phosphinate (LAP), which was kept at 37 °C in dark before use. Cell suspension was mixed with prepolymer solution at a 1:1 ratio immediately before printing to maximize viability. TNBC/CAF co‐culture models were two‐step printed using a light‐based bioprinter, Biocube (Cyberiad Biotechnology, China), with a wavelength of 405 nm and a power density (irradiance) of 50 mW/cm^2^ and a scaffold thickness of 0.3 mm. The CAF‐material mixture was firstly exposed to the bioprinter for an optimized 30 s duration to prepare the peripheral region, and washed with PBS. The TNBC‐material mixture was exposed to the bioprinter for an optimized 30 s duration to prepare the core region. Afterward, the bioprinted constructs were rinsed with PBS and cultured in a medium with or without CL8‐M6P_3_ or NanoCLY at 37 °C and 5% CO_2_.

### Western Blotting Analysis

BT549 and MDA‐MB‐231 cells were treated in different conditions. The cells were then collected and lysed in NP40 lysis buffer. Total protein was quantified using a BCA assay kit (P0010S, Beyotime). The proteins were separated by SDS‐PAGE and transferred onto a PVDF membrane. The PVDF membrane was blocked in TBST containing 5% BSA, and then incubated with primary antibodies, including CTGF Rabbit mAb (D8Z8U, CST), Smad3 Rabbit mAb (C67H9, CST), Phospho‐SMAD3 (Ser423/425) Rabbit mAb (C25A9, CST) Notch3 Rabbit mAb (D11B8, CST), Jagged1 Rabbit mAb (D4Y1R, CST), His‐Tag Rabbit mAb (D3I1O, CST), β‐tubulin mouse monoclonal antibody (LF203, Epizyme Biotech), and GAPDH mouse monoclonal antibody (LF205, Epizyme Biotech), overnight at 4 °C. Subsequently, membranes were incubated with horseradish peroxidase‐conjugated anti‐rabbit or anti‐mouse IgG secondary antibody (LF101, LF102, Epizyme Biotech), including for 1 h at room temperature. The membranes were analyzed by a ChemiDoc MP imaging system (Bio‐Rad, USA) and quantified by ImageJ software.

### Cleavage of CTGF by TNBC Cell Conditioned Medium

BT549 cells at 24 h post‐siCTGF transfection were seeded at 5 × 10^4^ cells per well in 12‐well plates. The medium from the 12‐well plate was collected after 48 h of cell culture. The recombinant human CTGF protein (1 × 10^−8^ _M_) was incubated with the conditioned medium at 37 °C with shaking for 24 h. After incubation, the mixture of CTGF and medium was added with 5 × loading buffer, and then tested as detailed in “Western blotting assay”.

### Biotin‐M6P*_n_* and CL8‐M6P_3_ Synthesis

The Biotin‐M6P*_n_* (*n* = 1, 2, and 3) were synthesized by Fmoc solid phase peptide synthesis (SPPS) and click chemistry. Rink amino resin with a sample loading of 0.54 mmol g^−1^ was used as a solid phase carrier to connect amino acids. A 20% piperidine solution in *N*,*N*‐dimethylformamide (DMF) was used to remove the Fmoc protecting group on the amino group. The condensation reaction was carried out using ethyl 2‐oxime cyanoacetate/N, N‐diisopropylcarbodiimide (DIC) system, which has high condensation efficiency and a strong ability to inhibit racemization. The Biotin‐PEG_3_‐(PraβAla)_3_ were cleaved from the solid support by treatment with TFA/triethylsilane/H_2_O (95:2.5:2.5, v/v/v) for 2 h and purified by semi‐preparative RP‐HPLC (10 × 250 mm) using a gradient of 5–65% CH_3_CN (0.1% TFA) in water (0.1% TFA), and the purified products were characterized by MS. For click chemistry, M6P azide (2.0 eq. per one alkyne group) dissolved in DMSO were added to peptide followed by addition of a solution of sodium ascorbate (1.0 eq. per one alkyne group) and CuSO_4_ (1.0 eq. per one alkyne group) in water for 24 h and purified by RP‐HPLC to get the final products.

For CL8‐M6P_3_ synthesis, CL8‐PEG_3_‐[Pra(M6P)βAla]_3_‐amino Resin was synthesized by SPPS. For click chemistry, 10 µmol CL8‐PEG_3_‐[Pra(M6P)βAla]_3_‐amino Resin (2.0 eq. per one alkyne group) dissolved in DMF (1 mL) was added to M6P azide (2.0 eq. per one alkyne group) dissolved in DMF (1 mL) followed by the addition of a solution of sodium ascorbate (1.0 eq. per one alkyne group), CuSO_4_ (1.0 eq. per one alkyne group) in ddH_2_O (100 µL) and Tris(benzyltriazolylmethyl)amine (TBTA, 0.5 equiv per one alkyne group) in DMF (100 µL). After shaking for 36 h, the CL8‐M6P_3_ were cleaved from the solid support by treatment with TFA/triethylsilane/H_2_O (95:2.5:2.5, v/v/v) for 2 h, and purified by semi‐preparative RP‐HPLC (10 × 250 mm) using a gradient of 5–65% CH_3_CN (0.1% TFA) in ddH_2_O (0.1% TFA), and the purified products were characterized by MS (Table S4, Supporting Information).

### NanoCLY Synthesis and Preparation

A solution of 1‐Dodecanethiol (0.5 mmol) and 2,2′‐Dithiodipyridine (0.6 mmol) in 10 mL MeOH was vigorously stirred for 3 h at room temperature. The reaction mixture was diluted with petroleum ether/ethyl acetate (50:1, v/v). The purified products 2‐(dodecyldisulfaneyl)pyridine were characterized by MS. CL8‐M6P_3_ (1.4 mg, 0.56 µmol, 1 eq.) and 2‐(dodecyldisulfaneyl)pyridine (261.7 µg, 0.84 µmol, 1.5 eq.) dissolved in 1 mL DMF/ddH_2_O (1/1, v:v) was vigorously stirred for 16 h at room temperature. The products were diluted with ddH_2_O. The aqueous layer was extracted with ethyl acetate three times, and filtered and concentrated under reduced pressure. For particle size measurement, NanoCLY was prepared at a final concentration of 500 µg/mL in PBS, sonicated for 30 min, and used for particle size analyzer (Malvern Panalytical, UK), transmission electron microscope (TEM) analysis (JEOL, Japan) and Nanosight NS300 (Malvern Panalytical, UK). 2‐(dodecyldisulfaneyl)pyridine, ESI‐MS calcd for C_17_H_29_NS_2_ [M + H]^+^ 312.55, found 312.20. NanoCLY, ESI‐MS calcd for C_107_H_176_N_27_O_47_P_3_S_2_ [M + H]^+^ 2750.77, found 1375.50.

### Microscale Thermophoresis (MST)

The affinity between various peptides (CL8, CC14, LT12, CC14S, CE8, FH14S, FH14, and CL8‐M6P_3_) and purified rhCTGF‐His tag protein (C15W, Novoprotein), IGFBP protein, and TSP proteins, was evaluated using microscale thermophoresis (NanoTemper, Germany). Additionally, the interaction between CL8‐M6P_3_ and the CCN1, CCN3, and CCN4 proteins was measured using the Dianthus system (NanoTemper, Germany). Prior to measurements, the purified proteins were labeled with fluorescent dye Monolith RED‐NHS (MO‐L011, NanoTemper). For the MST assay, 2 × 10^−6 ^M proteins (10 µL) were added with variable concentrations of the above peptides in PBST buffer (10 µL, PBS containing 0.05% Tween‐20). After a short incubation, the mixture was loaded into the Monolith NT.115 capillary (MO‐K022, NanoTemper) and analyzed using the Monolith NT.115 (NanoTemper, Germany).

### Proteins Colocalization with Lysosome

BT549 cells were plated at a density of 4 × 10^4^ cells per 20 mm diameter culture dish, and incubated overnight. For NA650 uptake, cells were incubated in a culture medium supplemented with 1 × 10^−7^ m NA650, or were co‐incubated with NA650 and 1× 10^−6^ m Biotin‐M6P_3_ at 37 °C for 6 h. For CTGF uptake, CTGF was labeled with a fluorescent dye (MO‐L011, NanoTemper) in advance. Cells were incubated in a complete growth medium supplemented with 2× 10^−8^ m fluorescent CTGF, or were co‐incubated with CTGF and 3 × 10^−6^ m CL8‐M6P_3_ or NanoCLY at 37 °C for 6 h. Lastly, the cells were stained with Lysotracker‐Green (Ex: 577 nm and Em: 590 nm, C1047S, Beyotime) at 7.5 × 10^−8^ m for 30 min to label lysosome, and were stained with Hoechst 33342 (C1022, Beyotime) at 5 × 10^−6^ m for 5 min to label the cell nuclei. Then, the cells were washed with cold PBS 3 times. Lastly, cells were observed by GE DeltaVision OMX SR (GE, USA) and analyzed with ImageJ software.

### CTGF Degradation Experiments

For evaluation of degradation time, BT549 and MDA‐MB‐231 cells were plated at a density of 2.5 × 10^5^ cells in 6‐well plates overnight. The cells were incubated with 2 × 10^−8^ m CTGF in the presence or absence of 1 × 10^−5^ m CL8‐M6P_3_ or NanoCLY. After 4 h, the cells were washed with PBS 3 times and were incubated with culture medium for 12 and 24 h, then lysed as detailed in “Western blotting assay”. For exploration of the degradation mechanism, the cells were incubated with 2 × 10^−8^ m CTGF in the presence or absence of 1 × 10^−5^ m CL8‐M6P_3_ or NanoCLY. After 4 h, the cells were washed with PBS 3 times, and were incubated with 0.1 mg mL^−1^ LPT, 5 × 10^−8^ m BafA1, and 1× 10^−5^ m CQ for 12 h, then lysed as detailed in “Western blotting assay.”

### Circular Dichroism Spectra

The CD spectra were used to explore the secondary structure of peptides on the BRIGHT TIME Chirascaspectropolarimeter (Applied Photophysics, Britain). CL8, CL8‐M6P_3_, and NanoCLY in 50% 2,2,2‐Trifluoroethanol (TFE) were recorded at 25 °C in a quartz cell of 1 mm and 0.5 mm path length, respectively. All spectra were converted to a uniform scale of molar ellipticity after background subtraction. The curves were smoothed using standard parameters.

### Protein Uptake Experiments

For NeutrAvidin650 (NA650, 84 607, Invitrogen) uptake experiment, 3 × 10^4^ BT549 cells were seeded in 24‐well plates or in a glass‐bottom dish and were incubated in complete growth medium supplemented with 1 × 10^−7^ _M_ NA650, or NA650 and 1 × 10^−6 ^_M_ Biotin‐M6P_n_ (*n* = 1, 2, 3) at 37 °C and 5% CO_2_. After incubation for 6 h, cells were harvested, washed with PBS, and centrifuged at 4 °C 300 × *g* for 3 min. Lastly, cells were tested by flow cytometer (Beckman Coulter, Germany), and analysis was performed using the FlowJo software. For observation, cells were washed three times with PBS, stained with Hoechst 33 342 to label the cell nuclei, and observed by GE DeltaVision OMX SR (GE, USA).

For the CTGF uptake experiment, 3 × 10^4^ BT549 cells were seeded in 24‐well plates. CTGF was labeled with fluorescent dye in advance. Cells were incubated in a complete growth medium supplemented with 2 × 10^−8^ m fluorescent CTGF and CL8‐M6P_3_ or CL8 (0, 1, 3, 10, 30 × 10^−6^ m) at 37 °C and 5% CO_2_. After incubation for 6 h, cells were harvested, washed with PBS, and centrifuged at 4 °C 300 × *g* for 3 min. Lastly, cells were tested by flow cytometer (Beckman coulter, Germany), and analysis was performed using the FlowJo software.

### Enzyme‐Linked Immunosorbent Assay (ELISA)

BT549 and MDA‐MB‐231 cells were treated with various concentrations of CL8‐M6P_3_ or NanoCLY for 12 h. Extracellular CTGF protein level in conditioned media was quantified by a human CTGF ELISA kit following the manufacturer's instructions (ab261851, Abcam and CSB‐E07875h, Cusabio). Extracellular CCN1, CCN3, and CCN4 protein levels were quantified using respective ELISA kits (HY‐P7842, HY‐P701327, HY‐P78051, MCE) following the manufacturer's instructions (CSB‐EL015956HU, CSB‐EL026119HU, CSB‐E13884h, Cusabio). Additionally, BSP and β‐CTx levels in serum were quantified by ELISA kit following the manufacturer's instructions (BPE10823, BPE10438, lengton).

### RNA Sequencing Analysis

Exponentially grown sh‐Control and sh‐CTGF BT549 cells (1 × 10^6^) were seeded into T25 cell culture flash and cultured for 24 h. BT549 cells (1 × 10^6^) were incubated with or without 1 × 10^−5 ^_M_ CL8‐M6P_3_. And breast CAFs (1 × 10^6^) were seeded into 6‐well plates and cultured for 24 h. Then, the CAFs were incubated with 5 × 10^−8^ m rhCTGF in the presence or absence of 1 × 10^−5^ m CL8‐M6P_3_ for 24 h and were collected. The total RNA was isolated using Trizol (B511311, Sangon Biotech) according to the manufacturer's instructions. The RNA purity was evaluated using a NanoDrop 2000/c spectrophotometer (Thermo Fisher Scientific, USA), and libraries were constructed using a VAHTS Universal V6 RNA‐seq Library Prep Kit. The transcriptome sequencing and analysis were conducted by OE Biotech. The libraries were sequenced on a Lumina Novaseq 6000 platform, and 150 bp paired‐end reads were generated. HISAT was used to map the clean reads to the reference genome. Fragments per kilobase per million of each gene were calculated, and the read count for each gene was obtained using HTSeq‐count. Then, principal component analysis was performed using R v.3.2.0. DEGs between two treatment groups were defined as having a q‐value <0.05 and fold‐change >1.2. The hypergeometric distribution of the DEGs was confirmed, and R v.3.2.0 was used to perform KEGG pathway enrichment analyses and generate the relevant figures.

### Quantitative Proteomics Analysis

For sample preparation, exponentially grown BT549 cells (1 × 10^6^) were seeded into T25 cell culture flash and cultured for 24 h. The cells were incubated with 1 × 10^−5^ m CL8‐M6P_3_ for 24 h. Cells were collected and added to NP‐40 buffer containing phosphatase inhibitor and protease inhibitor, lysed on ice for 30 min, and the total supernatant protein was quantified by BCA assay kit (P0010S, Beytime). The samples were added with acetone for 16 h, and lyophilization by a centrifugal concentrator. The proteins were added with 8 m urea for 1.5 h, 0.1 m 1,4‐Dithiothreitol (DTT) for another 1.5 h, and 0.5 m 2‐Iodoacetamide (IAA) for 40 min. Trypsin digestion buffer was added, and the mixture was incubated at 37 °C for 16 h with agitation at 400 rpm. Then, cleanup and desalination steps were conducted using the SPE Spin Centrifuge Columns (5010‐21701, GL Sciences), and then subjected to lyophilization by a centrifugal concentrator. The LC‐MS analysis was carried out on Q Exactive Plus Orbitrap LC‐MS/MS System (Thermo Fisher Scientific, USA). Peptides were separated over 115 min or 180 min with a water‐acetonitrile gradient containing 0.1% formic acid on a 25 cm long Aurora Series UHPLC column (lon Opticks) with 75 µm inner diameter. MS1 spectra were acquired at 120k resolution in the Orbitrap, MS2 spectra were acquired after ClD activation in the ion trap, and MS3 spectra were acquired after HCD activation with a synchronous precursor selection approach using 5 or 8 notches and 60k or 50k resolution in the Orbitrap.

For data analysis, the obtained spectra were analyzed with Proteome Discoverer 2.4 (Thermo Fisher Scientific). The database employed was homo sapiens sourced from the UniProt database. The false discovery rate (FDR) for proteins and peptides was set at 1%. Proteins were quantified and normalized using MaxLFQ with a label‐free quantification (LFQ) minimum ratio count of 2. In order to assess the two‐sided null hypothesis indicating no alterations in abundance, an unpaired t‐test was used to calculate the P values followed by Benjamini–Hochberg adjustment. Proteins that exhibited fold change greater between the two treatment groups were defined as having a *q*‐value < 0.05 and fold‐change >1.2.

### Cytokine Profiling

The Proteome Profiler Human XL Cytokine Array Kit (ARY022B, R&D systems) was used for the relative evaluation of corresponding proteins in CAF supernatants. CAFs were incubated with 5 × 10^−8^ m CTGF in the presence or absence of 3 × 10^−5^ m CL8‐M6P_3_ for 72 h. Culture supernatants were collected and spun down at 1200 × *g* and 4 °C for 10 min. Samples were analyzed using the Cytokine Array according to the manufacturer's instructions. The signal was visualized on a ChemiDoc MP imaging system.

### Quantitative Real‐time Polymerase Chain Reaction (qPCR)

Total RNA was extracted from cultured cells with FastPure Cell/Tissue Total RNA Isolation Kit V2 (RC112, Vazyme), and performed cDNA synthesis with 1 µg of total RNA with HiScript II Q RT SuperMix for qPCR kit (R223, Vazyme) based on the manufacturer's instructions. qRT‐PCR is performed to detect relevant mRNA expression with a ChamQ Universal SYBR qPCR Master Mix kit (Q711, Vazyme) and a LightCycler 96 Real‐Time PCR System (Roche, Switzerland). The mRNA expression level of IL‐6 or CXCL‐8 relative to GAPDH was assessed by the 2^−ΔΔ Ct^ method. The qRT‐PCR primer sequences are listed in Table S3 (Supporting Information).

### Hemolysis Assay

The hemolytic activities of CL8, CL8‐M6P_3,_ and NanoCLY were determined by monitoring hemoglobin release from red blood cells. Fresh erythrocytes were obtained from the whole blood of mice by centrifugation (1000 × *g* for 30 min at 4 °C), and washed three times with cold 0.9% NaCl. The erythrocytes were dispensed into 96‐well plates, incubated with CL8, CL8‐M6P_3_ and NanoCLY at specified concentrations (0, 0.3, 1, 3, 10, and 30 × 10^−6^ m) for 1 h at 37 °C, centrifuged at 1000 × *g* for 10 min, the supernatant samples were collected, and the absorbance at 570 nm was measured by microplate reader Cytation 5 (Biotek, USA). The negative control group was defined as the absorbance obtained in 1× PBS (*A*_PBS_), and 100% hemolysis in 0.1%Triton X‐100 (v/v) (*A*_Triton_). The hemolysis percentage was calculated as: (*A*_peptide_ − *A*_PBS_)/(A_Triton_ − *A*_PBS_) × 100%.

### Measurement of CMC

The CMC value of NanoCLY in an aqueous solution was estimated by pyrene. Pyrene with the final concentration of 6.08 mg L^−1^ was added 100 µL to Eppendorf tubes and dried overnight, and was added with 500 µL of various concentrations of NanoCLY (0.001, 0.006, 0.0125, 0.025, 0.05, 0.2, 0.6 mg mL^−1^), and sonicated for 2 h. After sonicating, the supernatant samples were collected, and recorded the intensity ratios of I390/I380 by microplate reader Cytation 5 (Biotek, USA). The crossing point of the two linear fittings of the logarithm of NanoCLY concentrations was set as the CMC value.

### Measurement of GSH Concentration

BT549 cells were seeded into 12‐well plates at a density of 5 × 10^4^ cells per well and the supernatant sample was collected after 24 h. For in vivo experiments, 3 × 10^6^ cells were injected into the fourth pair of breast pads on the left side of the female nude mice (4–6 weeks old). When the tumor volume reached about 200 mm^3^, tumors and blood were collected for GSH concentration measurement using a GSH Assay Kit following the manufacturer's instructions (S0053, Beyotime).

### GSH Response of NanoCLY

For in vitro experiments, NanoCLY (0.1 mg mL^−1^) was incubated with varying concentrations of GSH (0, 2, 25 × 10^−5^ m) in PBS at 37 °C for 6 h. After incubation, the aliquots were analyzed by NanoSight (Malvern Panalytical, UK), HPLC (Agilent, USA), and TEM (JEOL, Japan). For in vivo experiments, when tumor volume reached about 200 mm^3^, mice were intratumorally injected with 50 µL NanoCLY (2.5 mg kg^−1^), or NanoCLY (2.5 mg kg^−1^) with a 20‐min preinjection of H_2_O_2_ (2.5 × 10^−2 ^M). Tumors were collected after 4 h for HPLC analysis.

### Plasma Stability Assay

For the DLS assay, NanoCLY solution was mixed with 50% mouse plasma and incubated for different time periods. After incubation, the aliquots were analyzed using DLS. For the NanoCLY stability assay under simulated plasma GSH conditions, NanoCLY solution was mixed with 1.5 × 10^−6 ^_M_ GSH or 20% mouse plasma, and was incubated for different time periods. After incubation, the aliquots were diluted and analyzed by HPLC.

### Biodistribution of the NanoCLY

Orthotopic MDA‐MB‐231 tumor model was established as described above. When the tumor volume reached about 200 mm^3^, three mice from each group were intravenous injected with 100 µL DiR, DiR@NanoCLY (0.5 mg kg^−1^ DiR), respectively. After 2, 4, 8, 12, and 24 h, the mice were anesthetized and observed by an IVIS imaging system (VISQUE In Vivo Elite, Korea). Following in vivo imaging, the mice were sacrificed, and the tumors and major organs were harvested to determine the fluorescence intensity. The results were analyzed by the software VISQUE Clevue.

### Targeted Uptake of NanoCLY in Vitro

Exponentially grown BT549, MDA‐MB‐231, and MCF10A cells were seeded into 12‐well plates at a density of 5 × 10^4^ cells per well and incubated overnight. The cells were treated with 5 × 10^−6^ m FITC@NanoCLY for 0.5 and 2 h, washed twice with 1 × PBS, and detached with trypsin treatment. The cell pellet samples were resuspended in 1 × PBS, examined by FCM (Beckman coulter, Germany), and the data analysis was performed using the FlowJo software.

### In Vivo Antitumor Therapy

For the orthotopic xenograft MDA‐MB‐231 tumor model, 3 × 10^6^ cells were injected into the fourth pair of breast pads on the left side of the female nude mice (4–6 weeks old). To evaluate the antitumor effect of NanoCLY in vivo, the mice were treated with 10 mg kg^−1^ NanoCLY daily once the tumor volume reached ≈50 mm^3^, with CL8 peptide (10 mg kg^−1^, daily) and FG‐3019 (30 mg kg^−1^, every 3 days) as the positive control group and PBS as negative control group. The dose selection and administration frequency of NanoCLY were based on both references to previously reported protein degrader studies with similar mechanisms and effective concentrations,^[^ ^28^, ^54^, ^58^ ^]^ and our preliminary in vivo dose‐escalation experiments. Preliminary experimental results indicated that a dose of 10 mg kg^−1^ achieved a tumor inhibition rate of over 50%. Based on this, 10 mg kg^−1^ was selected for subsequent experiments. As for FG‐3019, the 30 mg kg^−1^ dose was chosen based on previous studies.^[^ ^43^, ^59^ ^]^ To evaluate the advantages of degradation strategies in vivo, the mice were treated with 10 mg kg^−1^ NanoCLY daily with Nano@CL8 (10 mg kg^−1^, daily) and PBS as control group. To evaluate the antitumor effect of combination therapy in vivo, the mice were treated with NanoCLY (10 mg kg^−1^, daily), PTX (5 mg kg^−1^, every two days), and PTX plus NanoCLY. The tumors were collected for western blotting assay (D5W4V, D8Z8U, CST; LF203, Epizyme Biotech) and ELISA (ab261851, Abcam; EK206, MULTI SCIENCES), and stained for CTGF, IL‐6, H&E, Ki‐67, and TUNEL assays, which were conducted by Wuhan Servicebio Technology Co., Ltd. Major organs (heart, liver, spleen, lung, and kidney) were also collected for H&E analysis by Wuhan Servicebio Technology Co., Ltd.

### Anti‐Lung Metastasis Efficacy in Vivo

Exponentially grown 5 × 10^5^ MDA‐MB‐231‐Luc cells were suspended in 100 µL of PBS and intravenously injected into female nude BALB/c mice. The mice were randomly divided into four groups (*n* = 3) and treated with PBS, 10 mg kg^−1^ NanoCLY via intravenous injection every 2 days for 12 times in total. The growth of lung metastatic tumors was monitored by VISQUE In Vivo Elite after intraperitoneal injection of 20 mg kg^−1^ D‐luciferin on day 12, day 18, and day 24. At the end of monitoring on day 24, the lungs were photographed and fixed, and H&E staining. Metastatic areas on the lung surface were quantified by ImageJ software.

### Anti‐Bone Metastasis Efficacy in Vivo

To establish the bone metastasis tumor model, 1 × 10^5^ MDA‐BoM‐1833 cells were injected into the left ventricle of female nude BALB/c mice (4‐6 weeks old), and surgery was monitored using a cardiac ultrasound instrument. The mice were randomly divided into 4 groups: PBS (control group, *n* = 6), 5 mg kg^−1^ PTX (*n* = 6), 10 mg kg^−1^ NanoCLY (*n* = 6), and PTX+NanoCLY (*n* = 6), starting on the designated day after tumor cell injection. Tumor cell metastasis was monitored using an in vivo imaging instrument (IVIS Spectrum, PerkinElmer, USA) by intraperitoneal injection of 20 mg kg^−1^ d‐luciferin.

### Statistical Analysis

Statistical analysis was performed with Student's *t*‐test or ANOVA with Tukey's multiple comparison tests via GraphPad Prism 8.0. All data are presented as means ± SD and represent three independent experiments. ns. stands for not significant.

## Conflict of Interest

The authors declare no conflict of interest.

## Supporting information



Supporting Information

## Data Availability

The data that support the findings of this study are available in the supplementary material of this article.
