# Supplementary material for: A Self‐Assembling LYTAC Mediates CTGF Degradation and Remodels Inflammatory Tumor Microenvironment for Triple‐Negative Breast Cancer Therapy
Source: Adv Sci (Weinh). 2025 May 11;12(23):2500311. doi: 10.1002/advs.202500311 (PMC12199414; doi:10.1002/advs.202500311)
Supplement: Supplementary file 1 — Supporting Information [file ADVS-12-2500311-s001.docx]

Supporting Information

A Self-assembling LYTAC Mediates CTGF Degradation and Remodels Inflammatory Tumor Microenvironment for Triple-Negative Breast Cancer Therapy

Jia-Yi Lin, Ye Wu, Xiao-Hui Liang, Min Tang, Xin Sun, Sheng-Xin Lu, Jin-Mei Jin, Xin Guo, Bei Wang, Hong-Zhuan Chen*, Wei-Dong Zhang*, and Xin Luan*

J.-Y. Lin, Y. Wu, X.-H Liang, M. Tang, X. Sun, S.-X Lu, J.-M Jin, X. Guo, B. Wang, H,-Z Chen, X. Luan

State Key Laboratory of Discovery and Utilization of Functional Components in Traditional Chinese Medicine, Shanghai Frontiers Science Center of TCM Chemical Biology, Institute of Interdisciplinary Integrative Medicine Research and Shuguang Hospital, Shanghai University of Traditional Chinese Medicine, Shanghai 201203, China

E-mail: [hongzhuan_chen@hotmail.com](mailto:hongzhuan_chen@hotmail.com); luanxin@shutcm.edu.cn

W.-D. Zhang

State Key Laboratory for Quality Ensurance and Sustainable Use of Dao-di Herbs, Institute of Medicinal Plant Development, Chinese Academy of Medical Sciences & Peking Union Medical College, Beijing, 100700, China

Email: wdzhangy@hotmail.com

W.-D. Zhang

School of Pharmacy, Second Military Medical University, Shanghai 200433, China

**Table of contents**

[**Figure S1.** Cleavage and knockdown of CTGF. 3](#_Toc192540111)

[**Figure S2.** The synthesis and characterization of Biotin-M6Pn. 4](#_Toc192540112)

[**Figure S3.** Design and characterization of M6P_3_-based LYTAC platform. 5](#_Toc192540113)

[**Figure S4.** MST analysis of various peptides binding to CTGF. 6](#_Toc192540114)

[**Figure S5.** The synthesis and characterization of CL8-M6P_3_. 6](#_Toc192540115)

[**Figure S6.** Binding affinity between the N-terminal domain, and the C-terminal domain of CTGF and CL8-M6P_3_. 7](#_Toc192540116)

[**Figure S7.** Analysis revealed that CL8-M6P_3_ had minimum effects on other CCN family members. 7](#_Toc192540117)

[**Figure S8.** CTGF-His level change without CL8-M6P_3_ after 12 h and 24 h. 8](#_Toc192540118)

[**Figure S9.** The synthesis and characterization of NanoCLY. 8](#_Toc192540119)

[**Figure S10.** Circular dichroism spectrum of CL8, CL8-M6P_3,_ and NanoCLY. 8](#_Toc192540120)

[**Figure S11.** Hemolysis activity of CL8, CL8-M6P_3,_ and NanoCLY. 9](#_Toc192540121)

[**Figure S12.** The plasma stability and GSH responsiveness of NanoCLY. 9](#_Toc192540122)

[**Figure S13.** The expression of CI-M6PR and NanoCLY degradation effects. 10](#_Toc192540123)

[**Figure S14.** The characterization and cellular uptake of FITC@NanoCLY. 10](#_Toc192540124)

[**Figure S15**. Biodistribution and tumor targeting of DiR@NanoCLY. 11](#_Toc192540125)

[**Figure S16.** Transwell assay. 11](#_Toc192540126)

[**Figure S17.** H&E staining of major organs. 12](#_Toc192540127)

[**Figure S18.** The complete blood panel analysis. 12](#_Toc192540128)

[**Figure S19.** H&E, Ki-67, and TUNEL staining of tumor samples. 13](#_Toc192540129)

[**Figure S20.** *In vivo* inhibition of MDA-MB-231 lung metastasis. 14](#_Toc192540130)

[**Figure S21.** Characterization of CL8@Nano. 14](#_Toc192540131)

[**Figure S22.** CTGF and IL-6 immunofluorescence staining. 15](#_Toc192540132)

[**Figure S23.** H&E, Ki-67, and TUNEL staining of tumor samples. 15](#_Toc192540133)

[**Figure S24.** H&E staining of major organs. 16](#_Toc192540134)

[**Figure S25.** The complete blood panel analysis. 16](#_Toc192540135)

[**Figure S26.** H&E staining of lungs. 17](#_Toc192540136)

[**Figure S27.** TRAP ratio in the mouse femoral heads. 17](#_Toc192540137)

[**Table S1.** shRNA sequences. 18](#_Toc192540138)

[**Table S2.** siRNA sequences. 18](#_Toc192540139)

[**Table S3.** qRT-PCR primer sequences. 18](#_Toc192540140)

[**Table S4.** The sequence, molecular formula, molecular weight, and observed MS of peptides. 19](#_Toc192540141)

[**References** 20](#_Toc192540142)

**
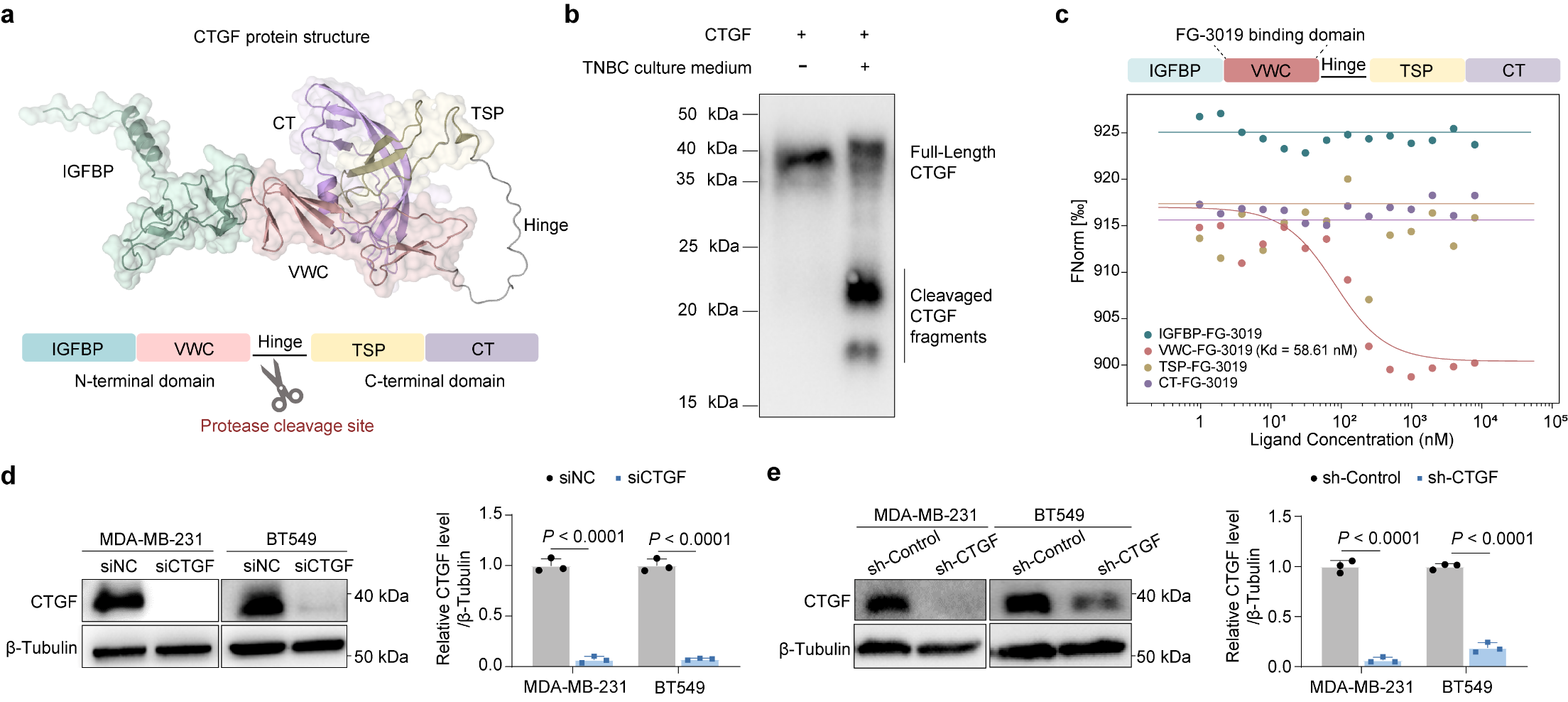
**

**Figure S1.** Cleavage and knockdown of CTGF. a) Structure of CTGF protein (AlphaFoldDB ID: AF-P29279-F1). b) Western blotting assay showing both full-length and cleaved fragments of CTGF. c) MST assays evaluating binding affinities of FG-3019 with IGBP, VWC, TSP, and CT, respectively. d, e) Western blotting assay and relative statistics of CTGF protein levels in MDA-MB-231, BT549 cells after siRNA and shRNA transfection (n = 3). The P values were calculated by one-way ANOVA. For d and e, the quantified data from different experiments were presented as the mean ± SD.

**
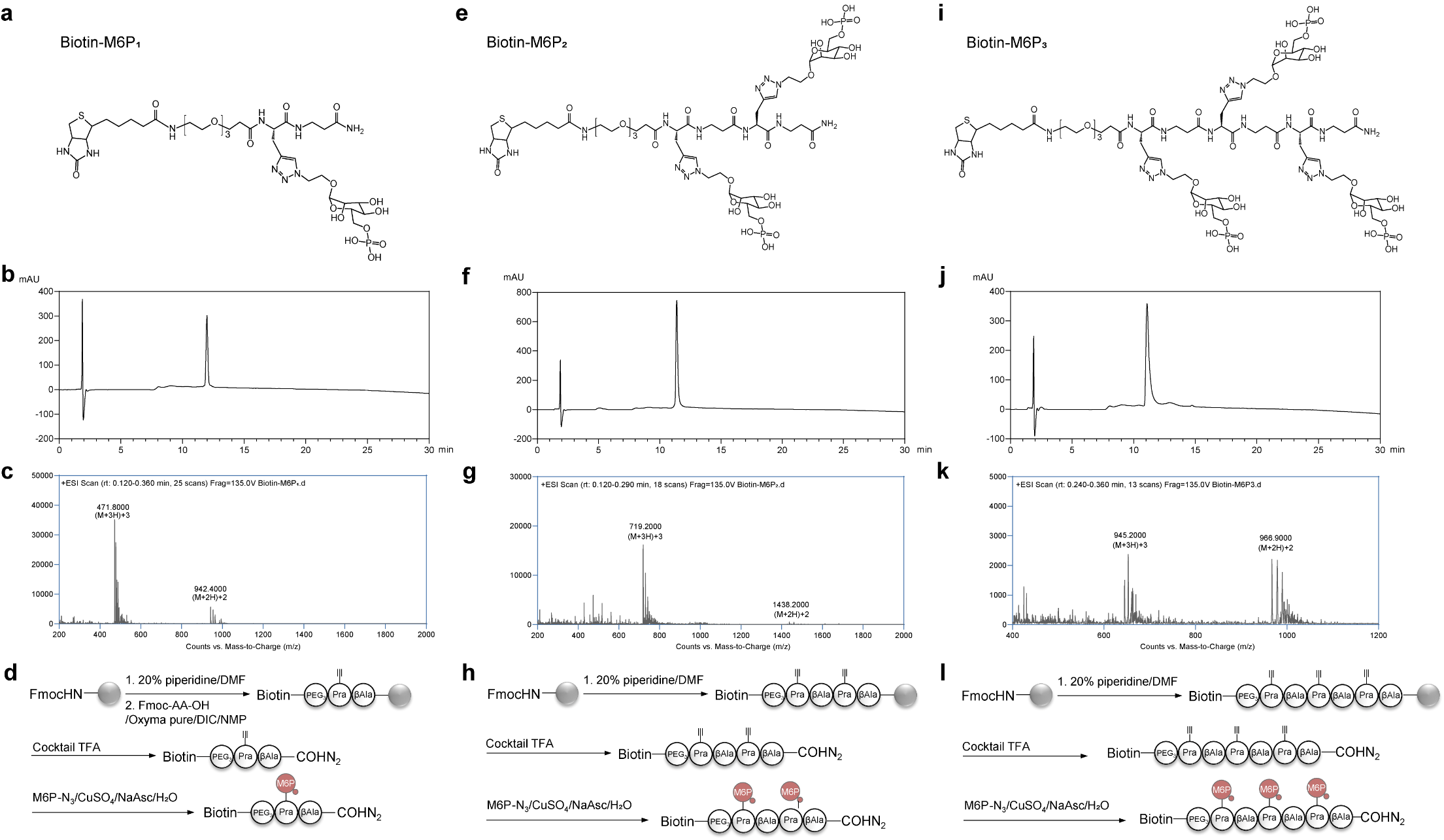
**

**Figure S2.** The synthesis and characterization of Biotin-M6Pn. a, e, i) Structures of Biotin-M6P_1_, Biotin-M6P_2_, and Biotin-M6P_3_. b, f, j) HPLC profiles of Biotin-M6P_1_, Biotin-M6P_2_, and Biotin-M6P_3_.

c, g, k) ESI-MS of Biotin-M6P_1_, Biotin-M6P_2_, and Biotin-M6P_3_. d, h, l) Synthetic route of Biotin-M6P_1_, Biotin-M6P_2_, and Biotin-M6P_3_.

**
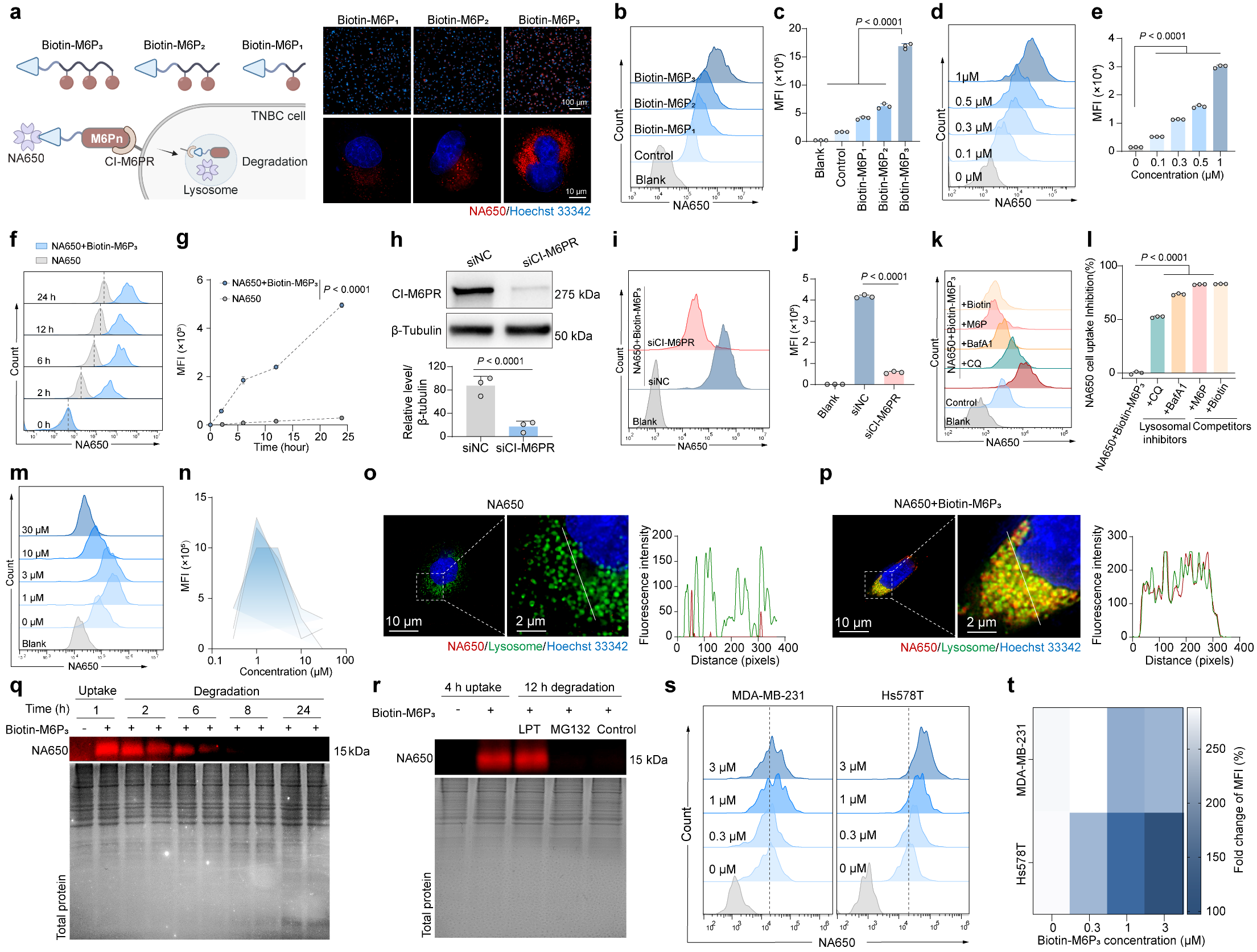
**

**Figure S3.** Design and characterization of M6P_3_-based LYTAC platform. a) Live-cell confocal microscopy images of BT549 cells treated with 1 × 10^-7^ _M_ NA650 and 1× 10^-6^ _M_ Biotin-M6P_1, 2, 3_. b, c) FCM results and relative statistics of BT549 cells treated as in a) (n = 3). The P values were calculated by one-way ANOVA. d, e) FCM results and relative statistics of BT549 cells treated with NA650 and Biotin-M6P_3_ at different concentration (n = 3). The P values were calculated by one-way ANOVA. f, g) FCM results and relative statistics of BT549 cells treated with NA650 and Biotin-M6P_3_ for different time (n = 3). The P values were calculated by two-way ANOVA. h-j) FCM results and relative statistics of BT549 (siNC and siCI-M6PR) treated with 100 nM NA650 and 1× 10^-6^ _M_ Biotin-M6P_3_ (n = 3). The P values were calculated by one-way ANOVA. k, l) NA650 cellular uptake in BT549 cells was inhibited by CQ, BafA1, excess M6P or Biotin (n = 3). The P values were calculated by one-way ANOVA. m, n) Hook effect of Biotin-M6P_3_, measured by FCM (n = 3). o, p) Confocal microscopy images of BT549 cells and colocalization analysis of NA650 and lysosomes in BT549 cells. q) In-gel fluorescence analysis of BT549 cells treated with NA650 and Biotin-M6P_3_, followed by washing and lysis after different times. r) In-gel fluorescence analysis of BT549 cells treated with NA650 and Biotin-M6P_3_, followed by washing and replacement of culture medium with LPT and MG132 for 12 hours. s, t) NA560 cellular uptake and heat maps showing the fold change of MFI in MDA-MB-231 and Hs578T cells (n = 3). For (c, e, g, h, j, l), the quantified data from different experiments were presented as the mean ± SD.

**
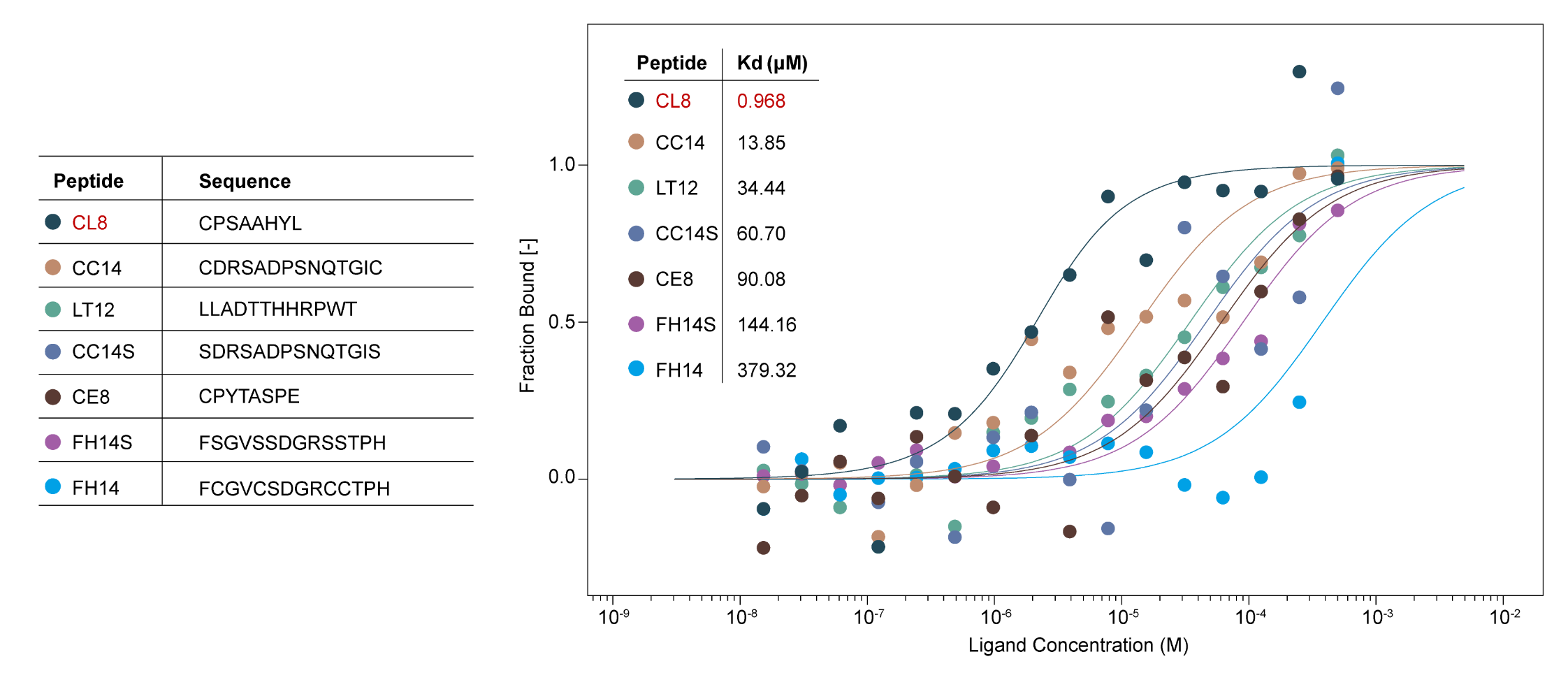
**

**Figure S4.** MST analysis of various peptides binding to CTGF. CL8 and CE8 peptides were screened by the bacteriophage display library.^[1]^ The LT12 peptide was developed by phage display technology.^[2]^ CC14, CC14S, FH14S, and FH14 peptides were designed in a patent.^[3]^

**
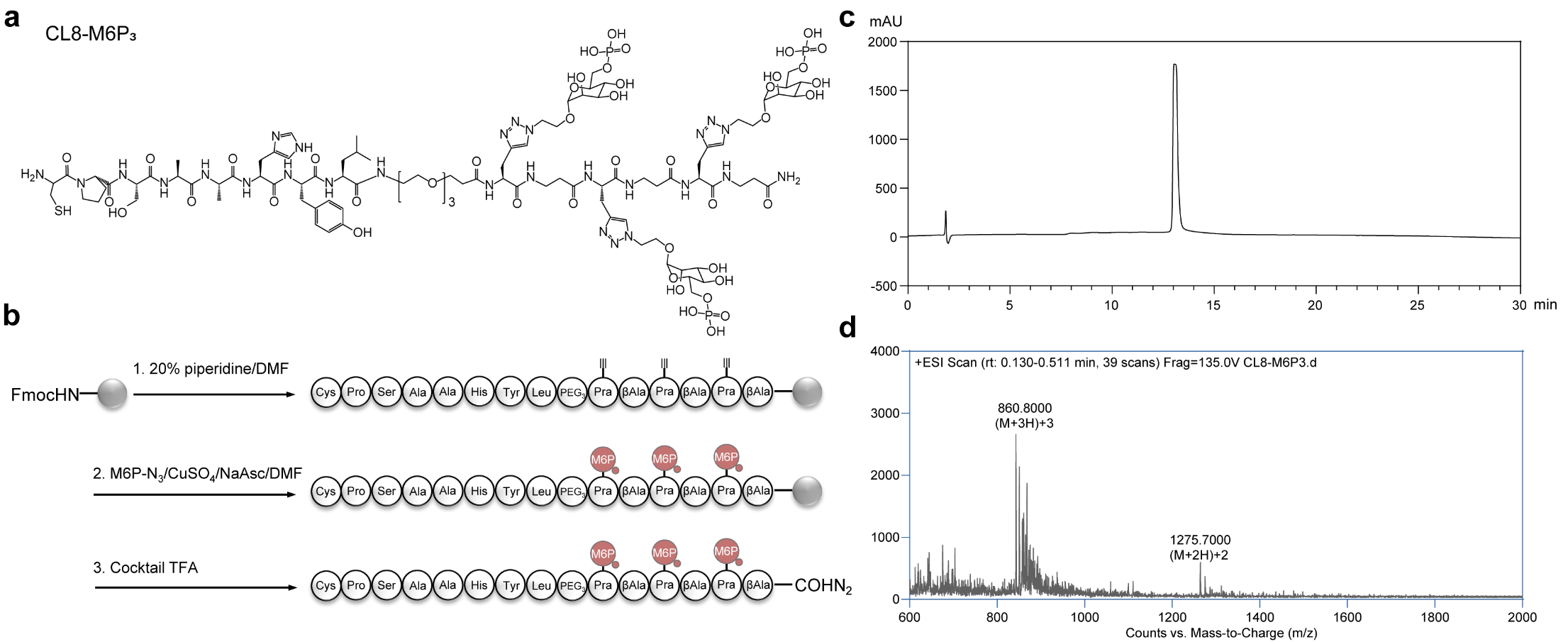
**

**Figure S5.** The synthesis and characterization of CL8-M6P_3_. a) Structure, b) Synthetic route, c) HPLC, and d) ESI-MS of CL8-M6P_3_.


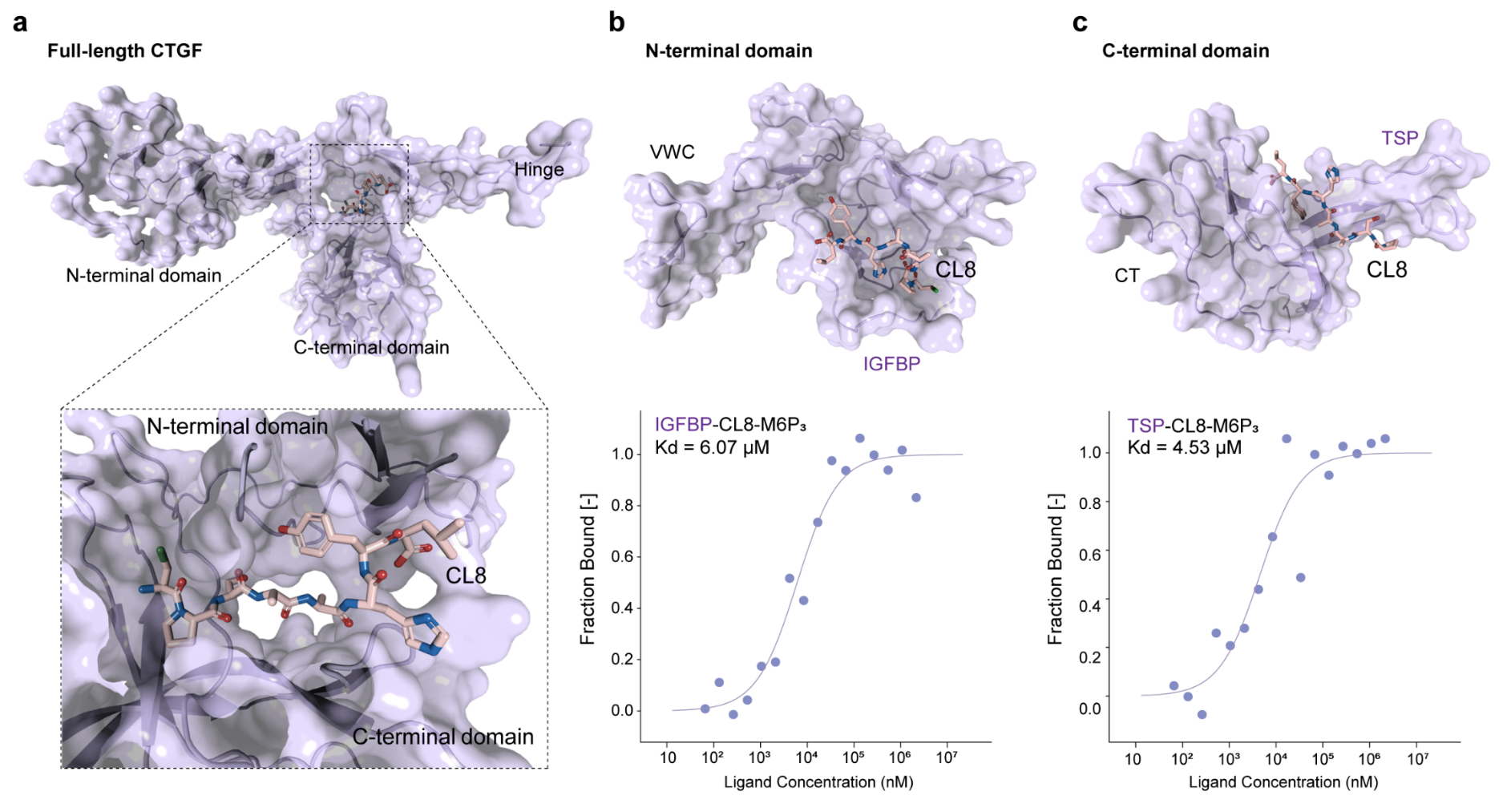


**Figure S6.** Binding affinity between the N-terminal domain and the C-terminal domain of CTGF and CL8-M6P_3_. a) Global docking of full-length CTGF and CL8 using the CABS-dock server. Global docking and MST analysis showing binding interactions between b) IGFBP and CL8-M6P_3_, and c) TSP and CL8-M6P_3_.


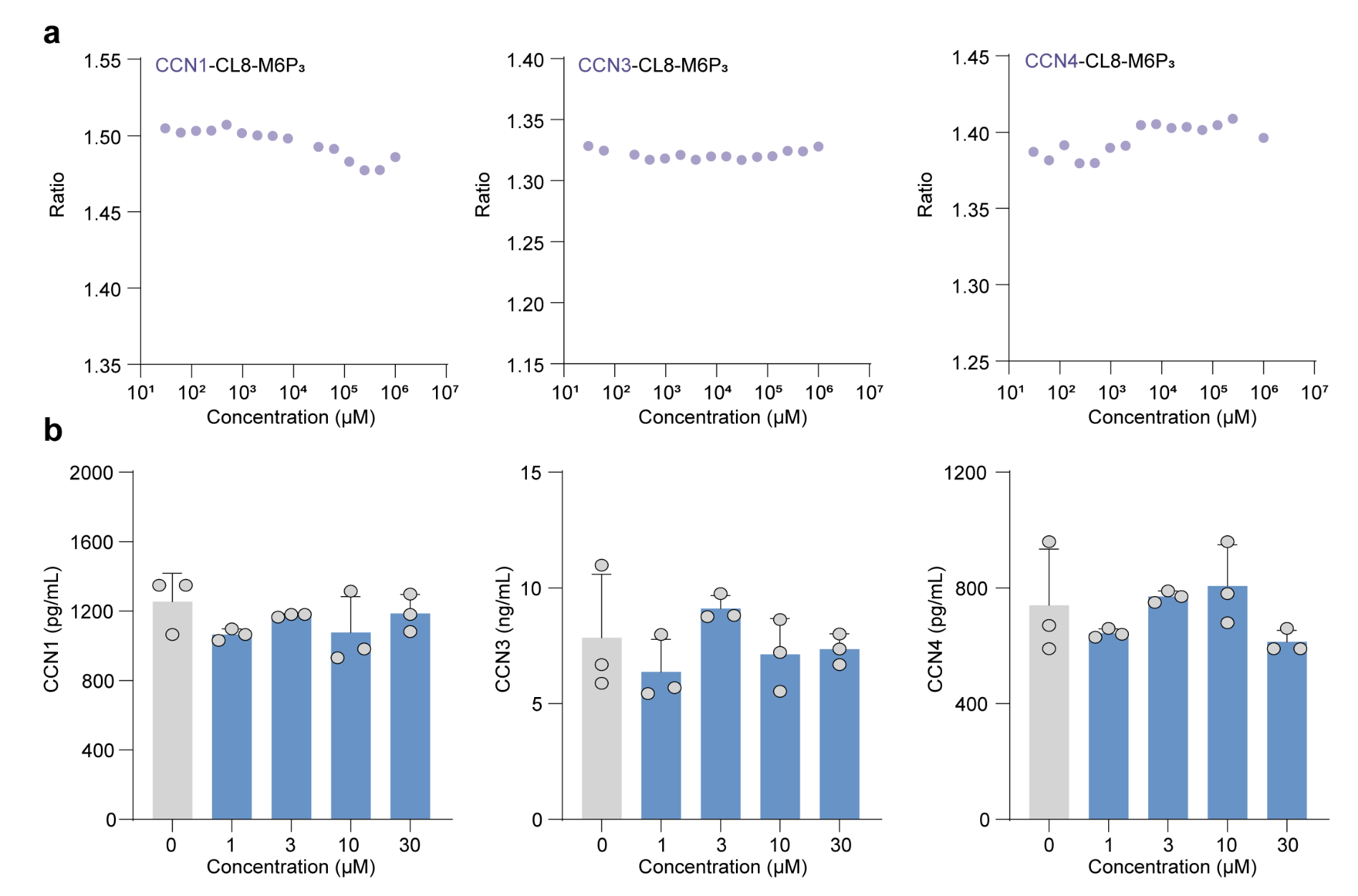


**Figure S7.** Analysis revealed that CL8-M6P_3_ had minimum effects on other CCN family members. a) MST analysis showing no binding interactions between other CCN family members and CL8-M6P_3_. b) CCN1, CCN3, and CCN4 ELISA of CL8-M6P_3_ in BT549 cell line.


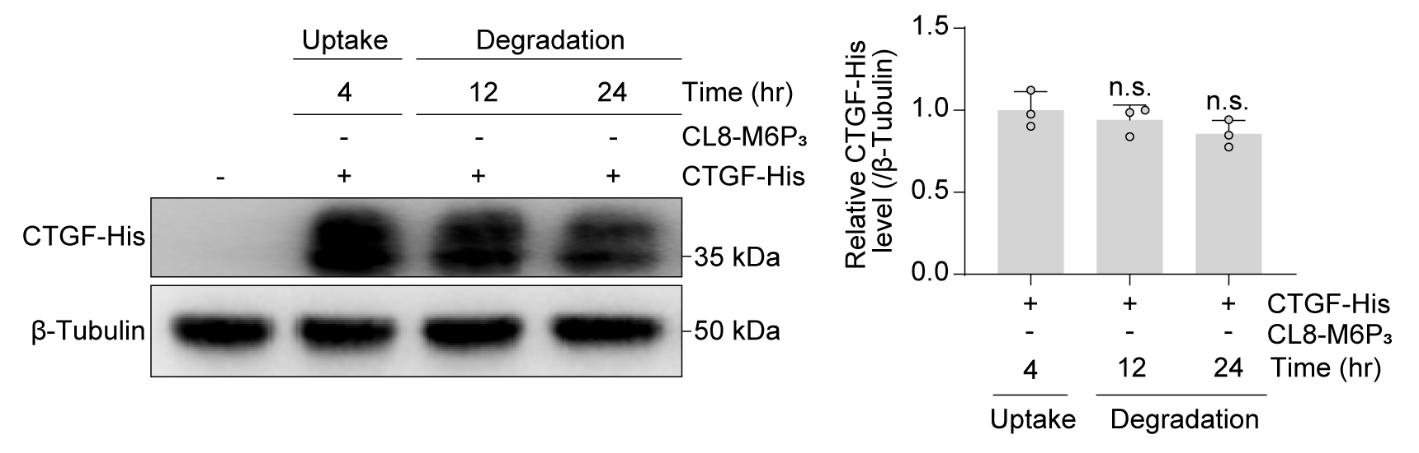


**Figure S8.** CTGF-His level changes without CL8-M6P_3_ after 12 h and 24 h.

**
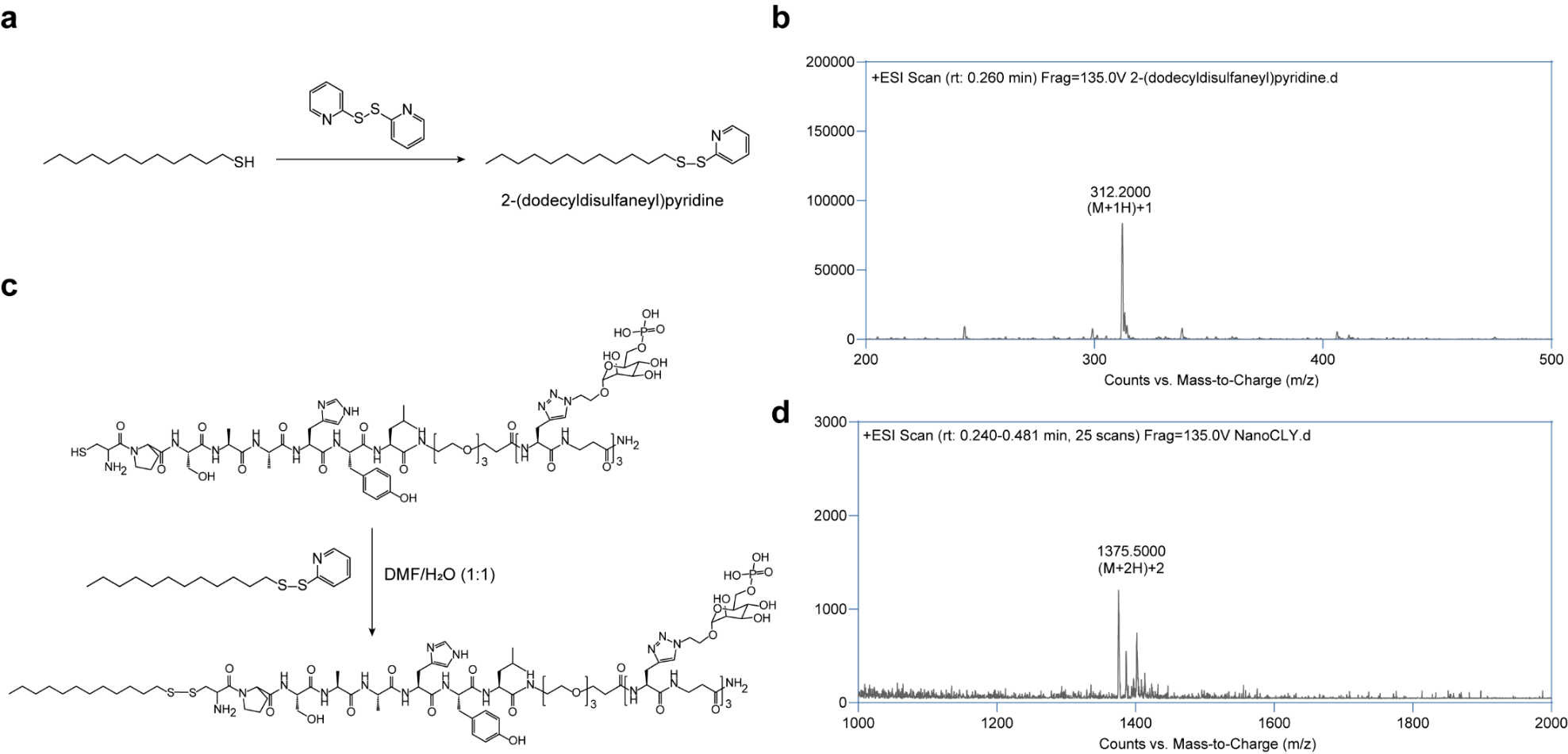
**

**Figure S9.** The synthesis and characterization of NanoCLY. a) Synthesis route and structure and b) ESI-MS of 2-(dodecyldisulfaneyl)pyridine. c) Synthesis route and structure, and d) ESI-MS of NanoCLY.

# **Figure S10.** Circular dichroism spectrum of CL8, CL8-M6P_3,_ and NanoCLY.

**
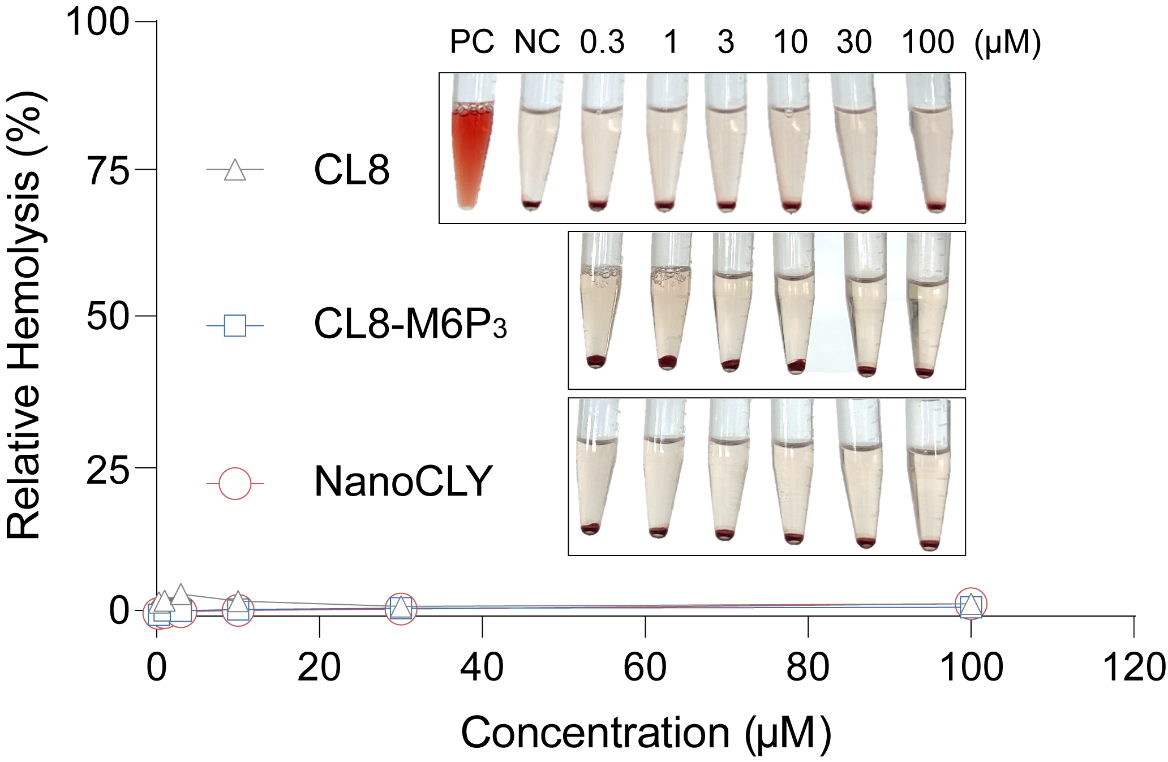
**

# **Figure S11.** Hemolysis activity of CL8, CL8-M6P_3,_ and NanoCLY.


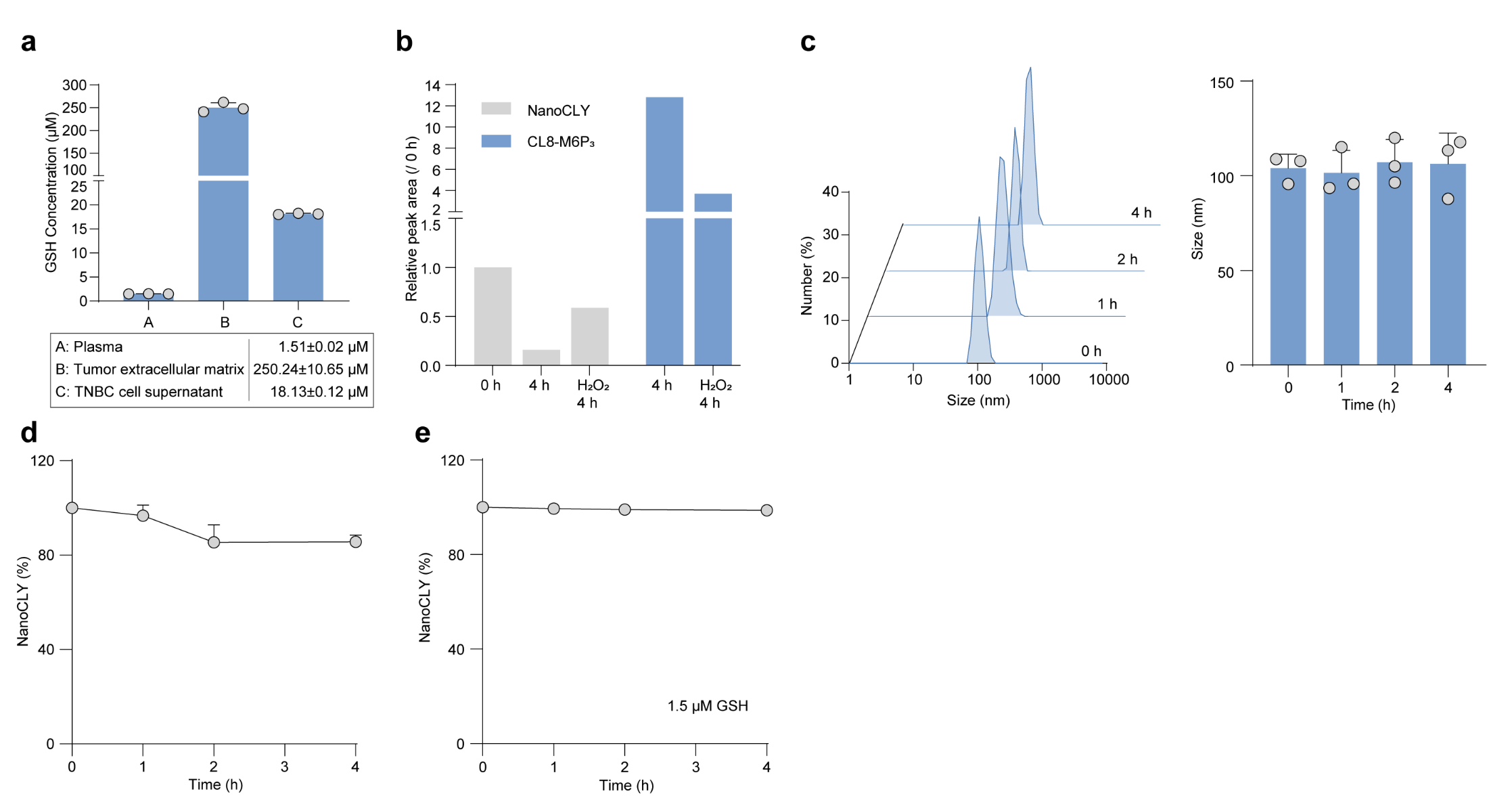


**Figure S12.** The plasma stability and GSH responsiveness of NanoCLY. a) GSH concentration in mouse plasma, TNBC tumor matrix, and TNBC cell supernatant (n = 3). b) The *in vivo* GSH responsiveness of NanoCLY at tumor sites. c) The size of the NanoCLY after incubation in 50% mouse plasma for different times (n = 3). d) HPLC analysis of NanoCLY under 20% plasma for different times (n = 3). e) HPLC analysis of NanoCLY under simulated plasma GSH concentration (1.5 × 10^-6^ M).


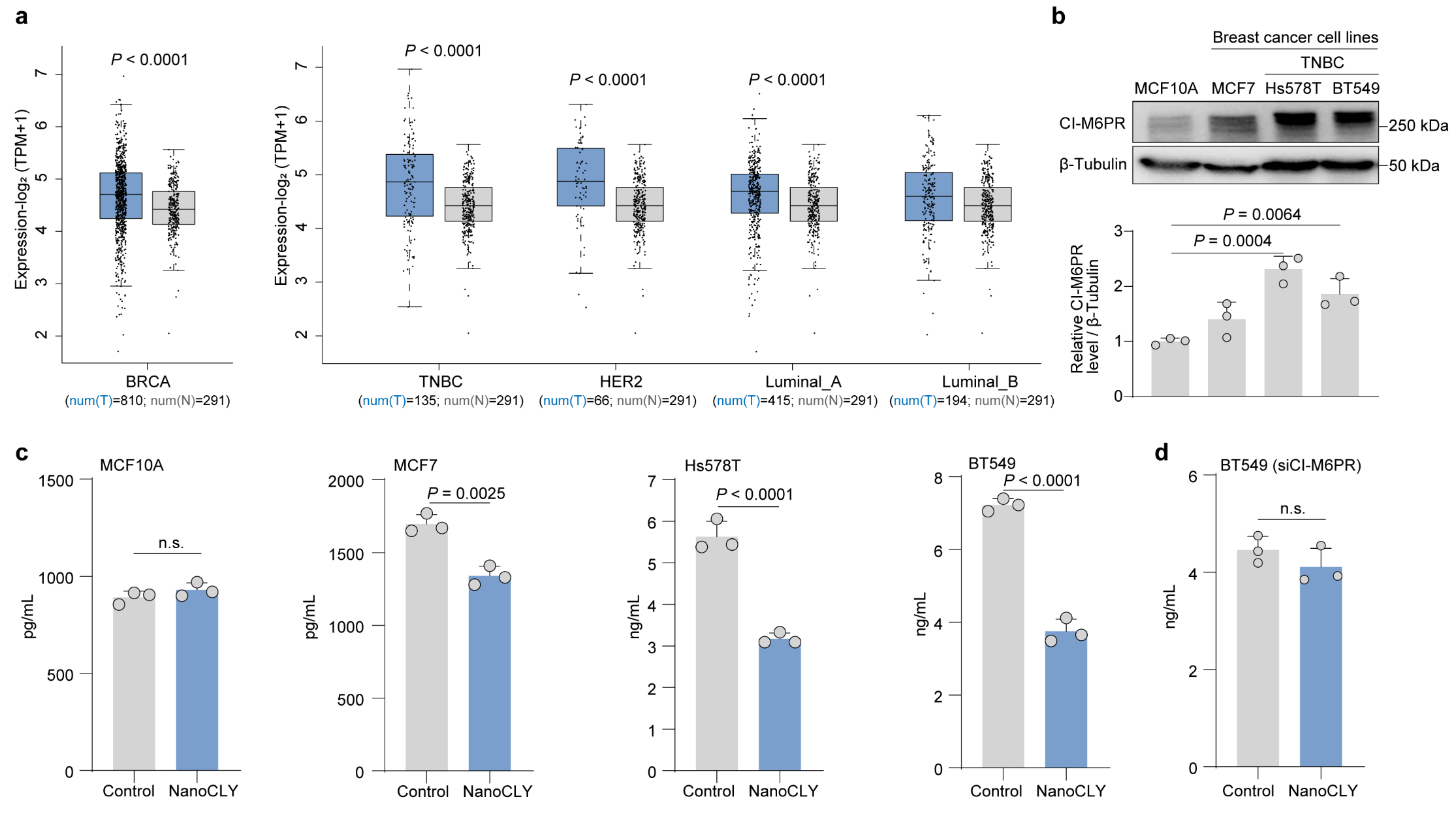


**Figure S13.** The expression of CI-M6PR and NanoCLY degradation effects. a) Increased expression of CI-M6PR was observed in patients with different molecular subtypes of breast cancer by GEPIA2 (http://gepia2.cancer-pku.cn/). b) Western blotting analysis of CI-M6PR protein level in normal breast epithelial cell MCF10A and breast cancer cell lines. c) CTGF ELISA in MCF10A, MCF7, BT549, and Hs578T after incubation with NanoCLY for 8 h. d) CTGF ELISA in BT549 (siCI-M6PR) after incubation with NanoCLY for 8 h. The P values were calculated by one-way ANOVA. Data shown are mean ± SD (n = 3).

**
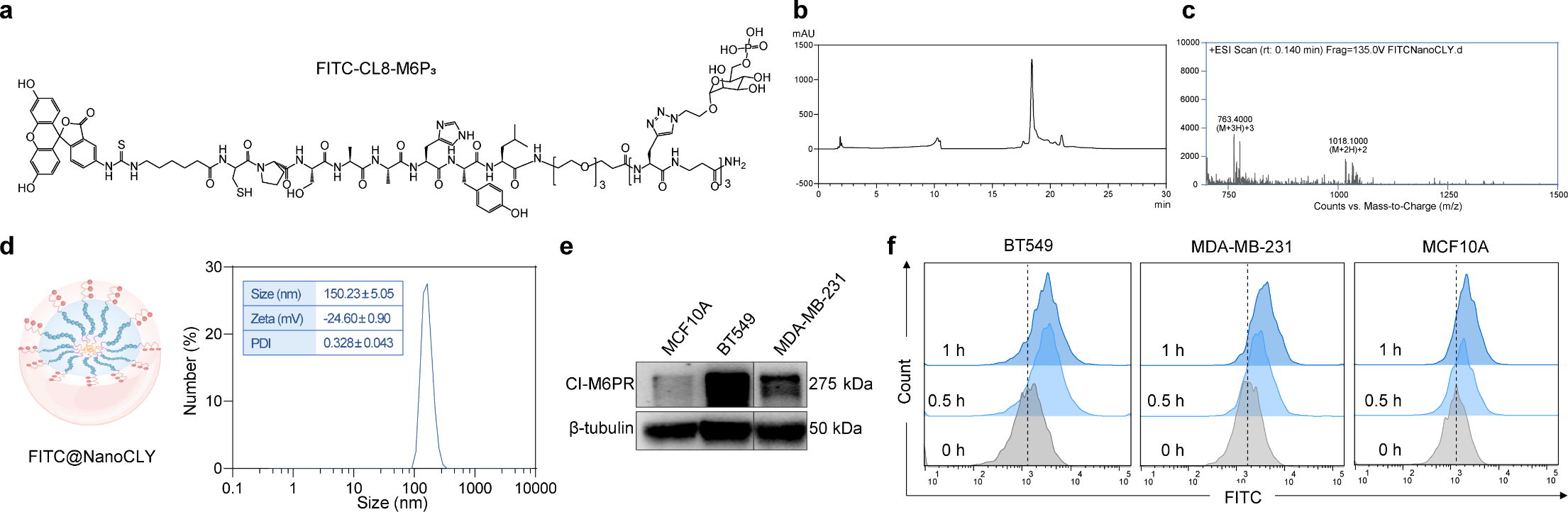
**

**Figure S14.** The characterization and cellular uptake of FITC@NanoCLY. a) Chemical structure of, b) HPLC, e) ESI-MS, and d) particle size of FITC-CL8-M6P_3_. e, f) Western blotting assay and cellular uptake of FITC@NanoCLY in BT549, MDA-MB-231, and MCF10A cells.

**
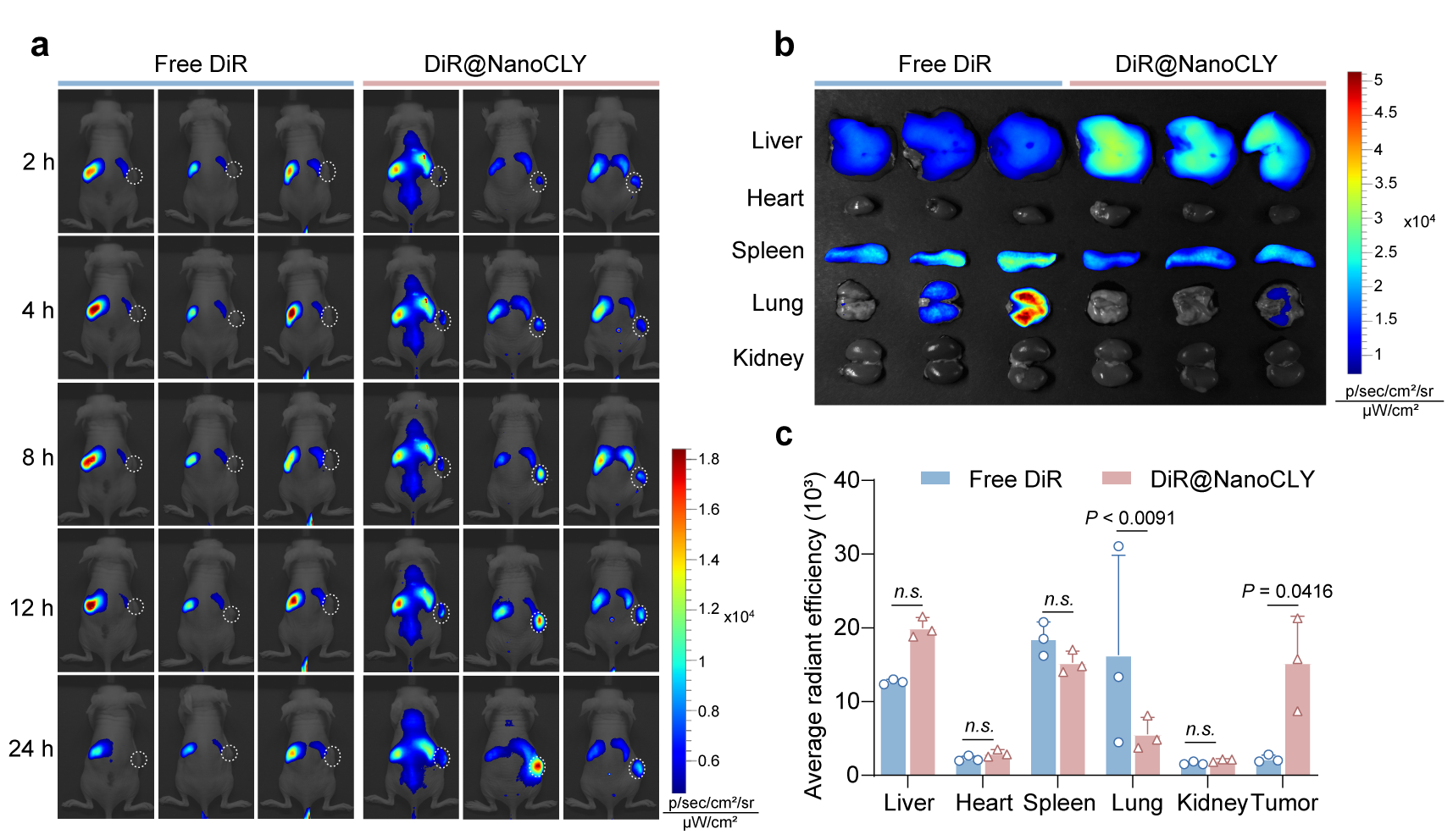
**

**Figure S15**. Biodistribution and tumor targeting of DiR@NanoCLY. a) Mice bearing MDA-MB-231 tumors (~200 mm^3^) received a single i.v. injection of free DiR or DiR@NanoCLY (containing DiR at 0.5 mg kg^-1^). *In vivo* DiR fluorescence was imaged at the specified time points post-injection using Bio-Imaging Technologies (VISQUE In Vivo Elite). b) Fluorescence of major organs at 24 hours after injection. c) Quantification of the fluorescence intensity in major organs and tumors. The P values were calculated by one-way ANOVA. Data shown are mean ± SD (n = 3).

**
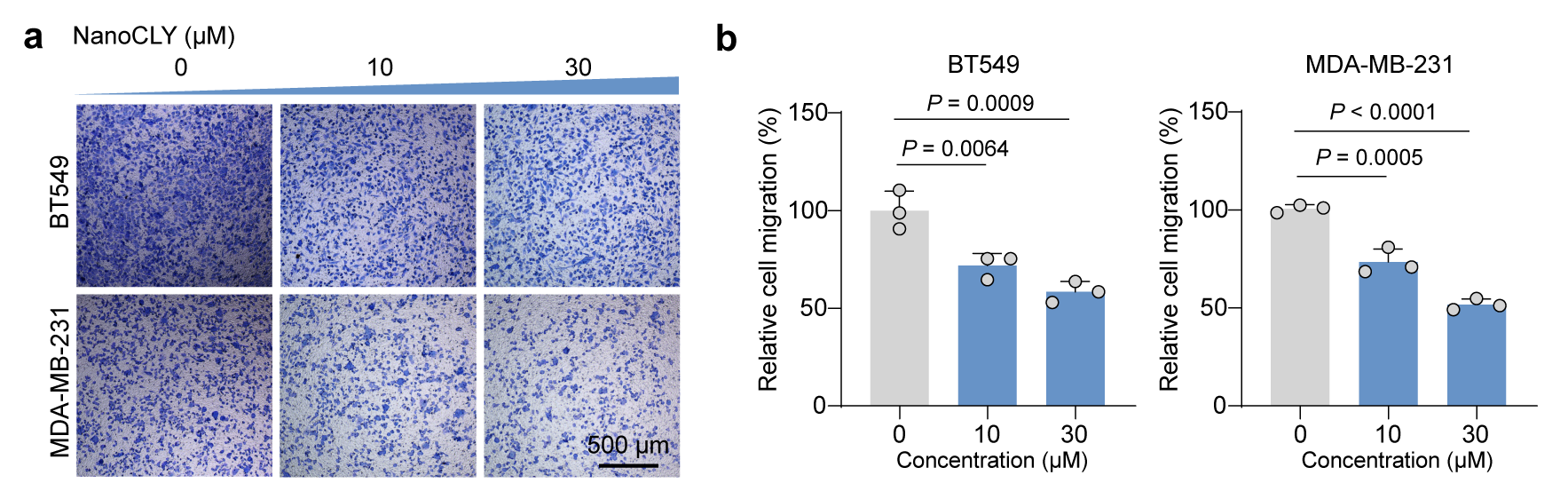
**

**Figure S16.** Transwell assay. a, b) Transwell assay and relative statistics analysis of BT549 and MDA-MB-231 cells treated with NanoCLY at different concentrations. The P values were calculated by one-way ANOVA. Data shown are mean ± SD (n = 3).

**
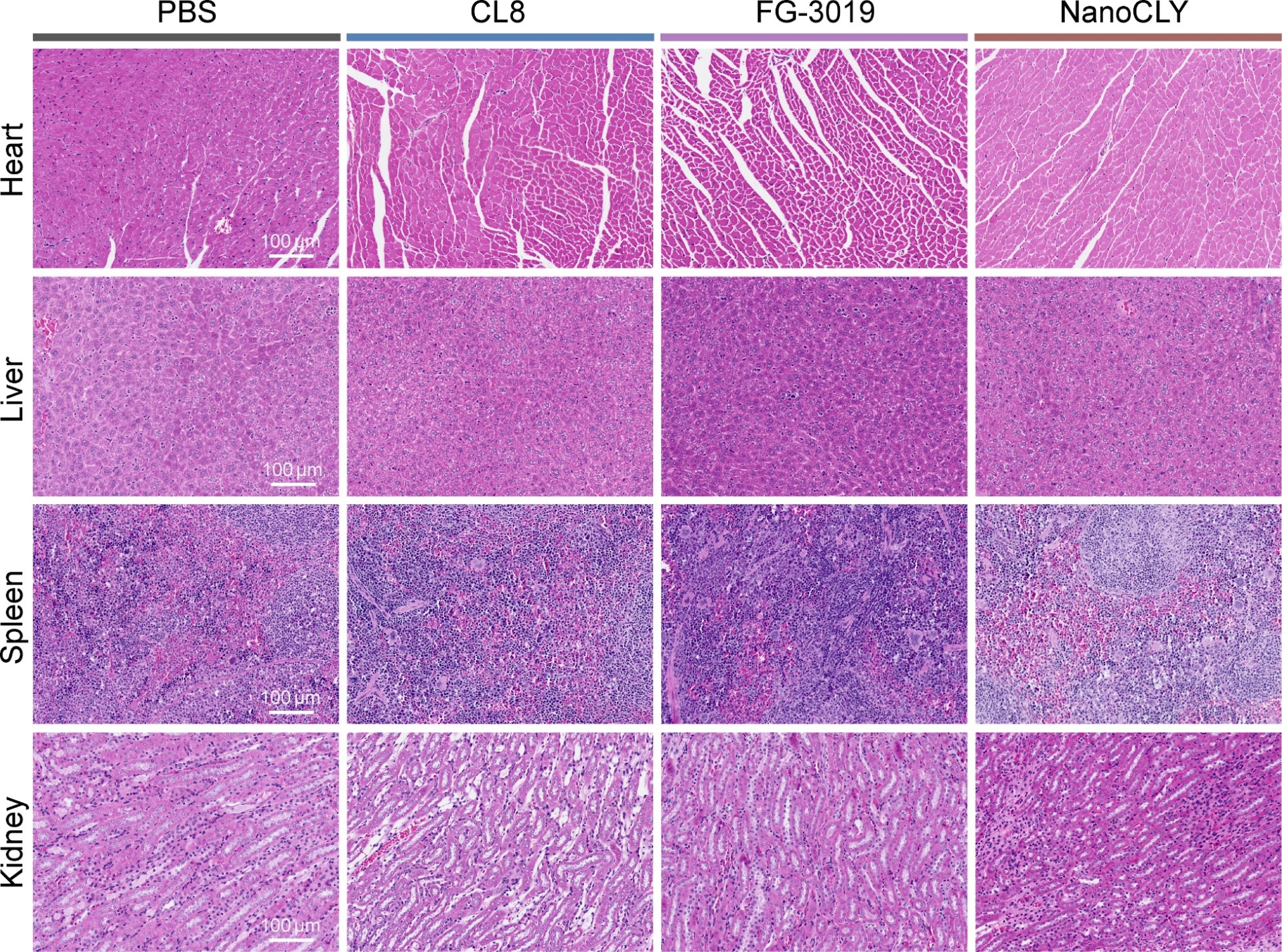
**

**Figure S17.** H&E staining of major organs. Representative images of H&E staining of the heart, liver, spleen, and kidney collected from groups treated with PBS, CL8, FG-3019, and NanoCLY in MDA-MB-231 orthotopic models at the end of observation (n = 3).

**
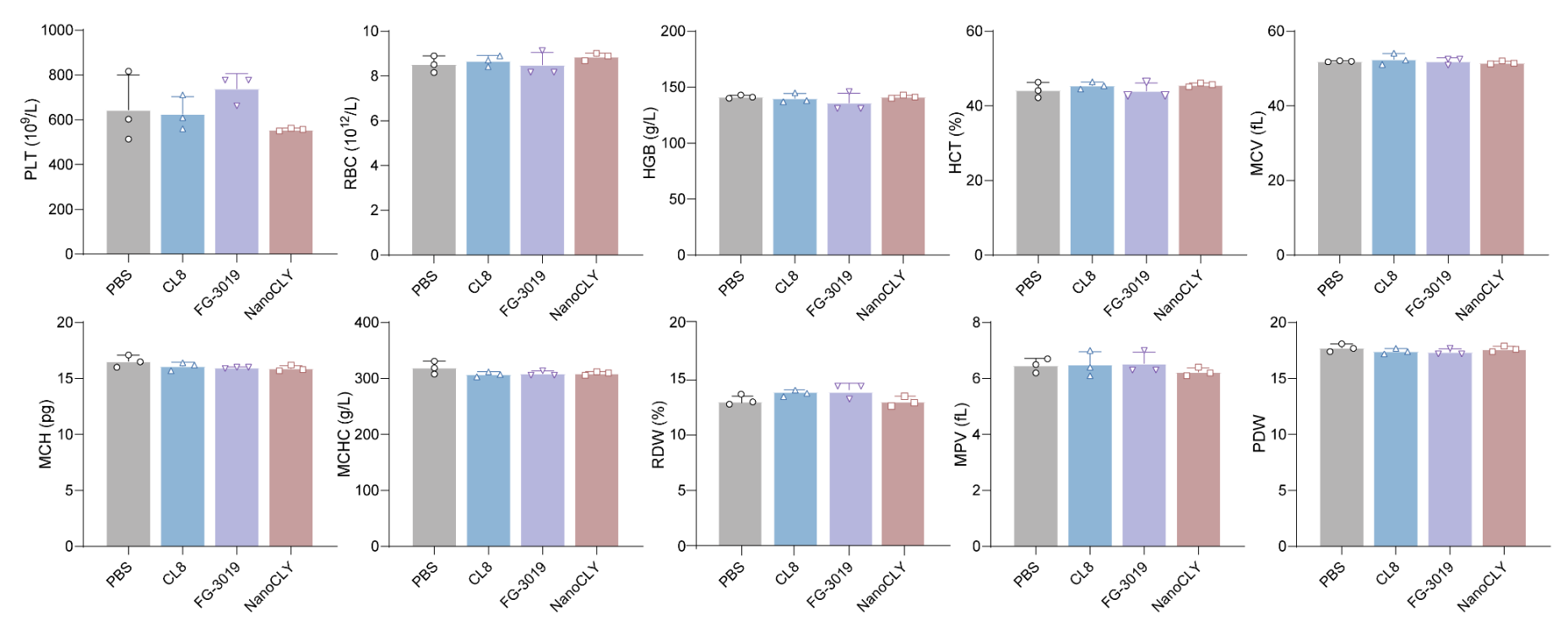
**

**Figure S18.** The complete blood panel analysis. The complete blood panel analysis of the mice bearing MDA-MB-231 tumors from groups treated with PBS, CL8, FG-3019, and NanoCLY at the end of observation. PLT, platelet. RBC, red blood cells. HGB, hemoglobin. HCT, hematocrit. MCV, mean corpuscular volume. MCH, mean erythrocyte hemoglobin content. MCHC, mean corpuscular hemoglobin concentration. RDW, red blood cell volume distribution width. MPV, mean platelet volume. PDW, platelet distribution width (n = 3).

**
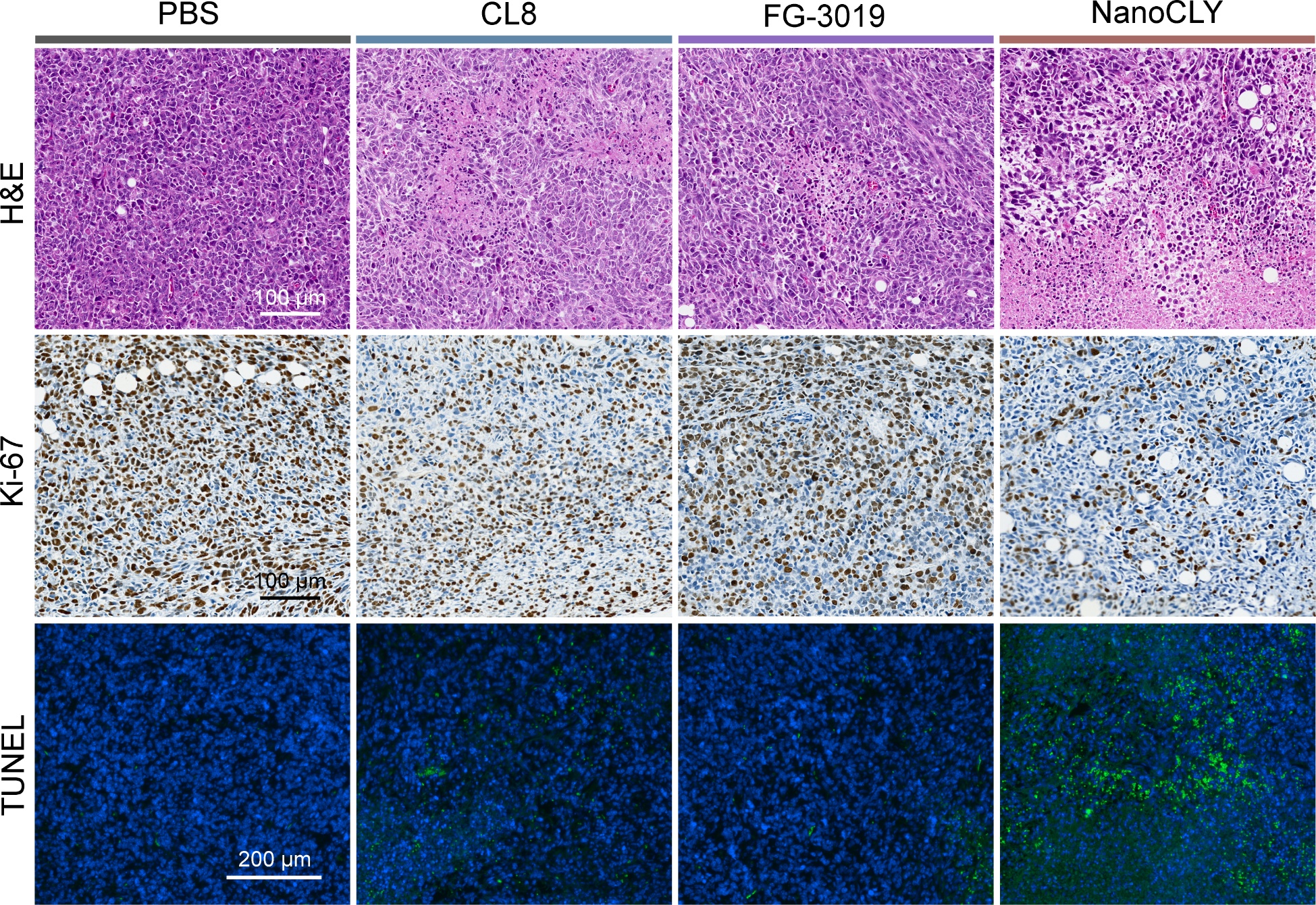
**

**Figure S19.** H&E, Ki-67, and TUNEL staining of tumor samples. Representative images of H&E, Ki-67, and TUNEL staining of MDA-MB-231 tumor samples collected from groups of PBS, CL8, FG-3019, and NanoCLY at the end of observation (n = 3).

**
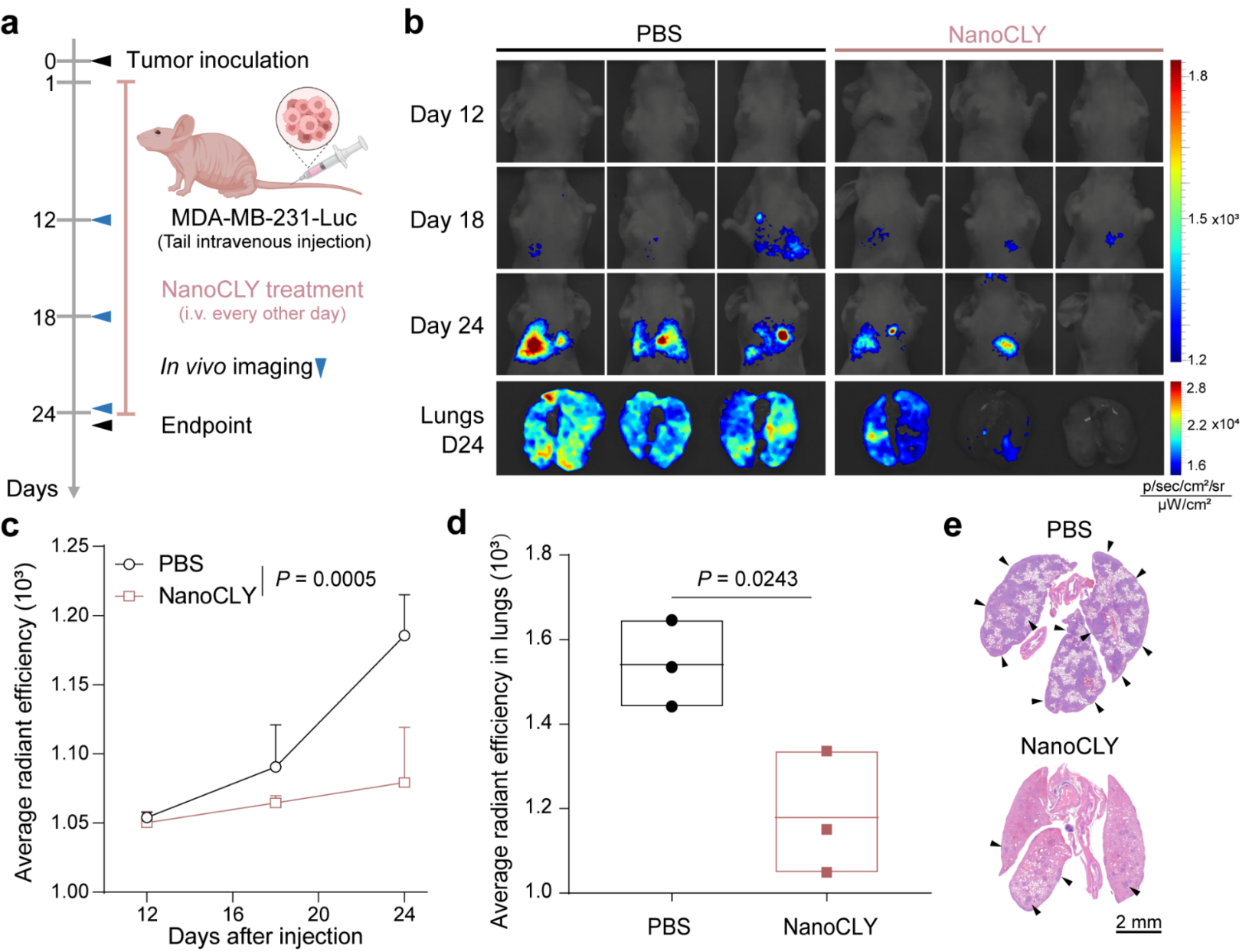
**

**Figure S20.** *In vivo* inhibition of MDA-MB-231 lung metastasis. a) Schematic diagram of the MDA-MB-231 tumor pulmonary metastasis experiment. b) Mice with *in vivo* luciferase fluorescence were imaged at a pre-determined time after injection using Bio-Imaging Technologies (VISQUE In Vivo Elite). c) Quantification of the fluorescence intensity at various times after injection. The P values were calculated by two-way ANOVA. d) Quantification of the fluorescence intensity in the lungs at the end of the observation period. The P values were calculated by one-way ANOVA. Data shown are mean ± SD (n = 3). e) Representative H&E-stained lung images in two groups. The black arrow marks the metastatic lesions.


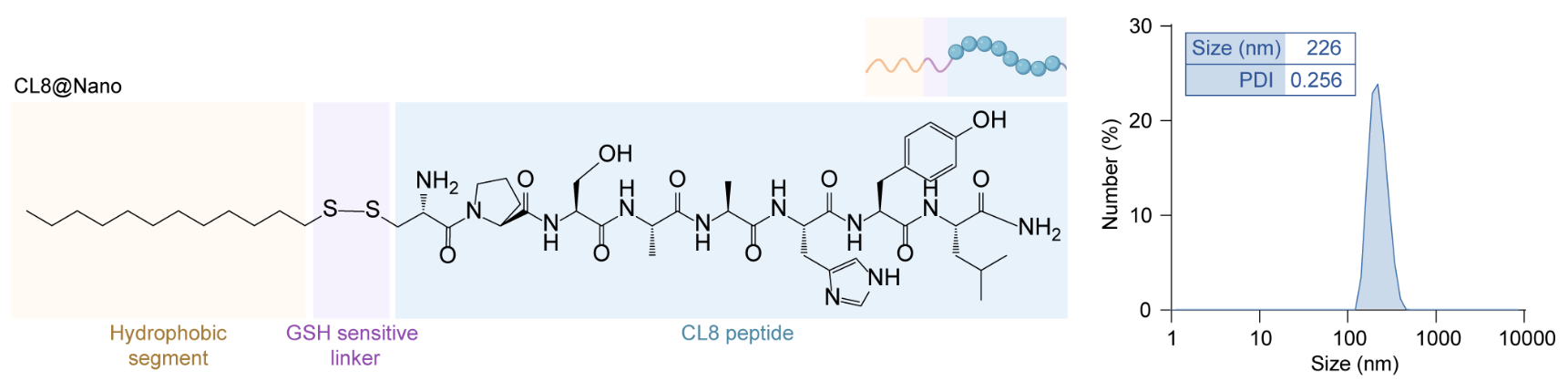


**Figure S21.** Characterization of CL8@Nano.

**
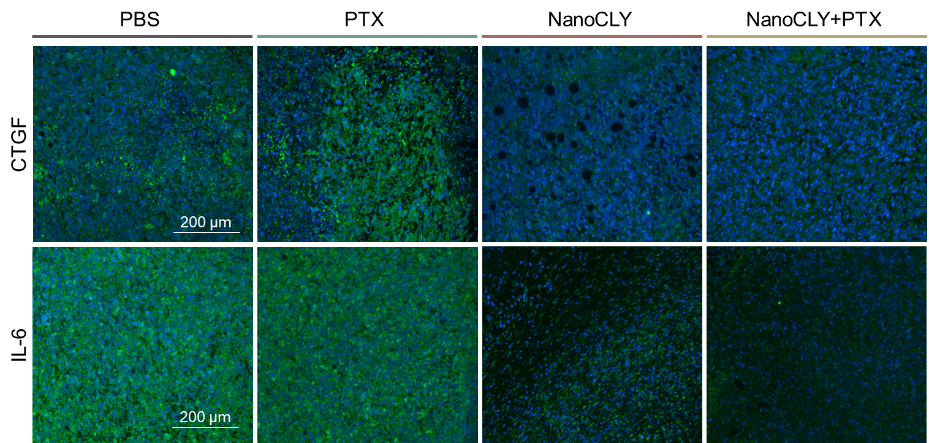
**

**Figure S22.** CTGF and IL-6 immunofluorescence staining. Representative images of CTGF and IL-6 immunofluorescence staining of MDA-MB-231 tumor samples collected from groups of PBS, PTX, NanoCLY, and NanoCLY+PTX treatment at the end of observation (n = 3).

**
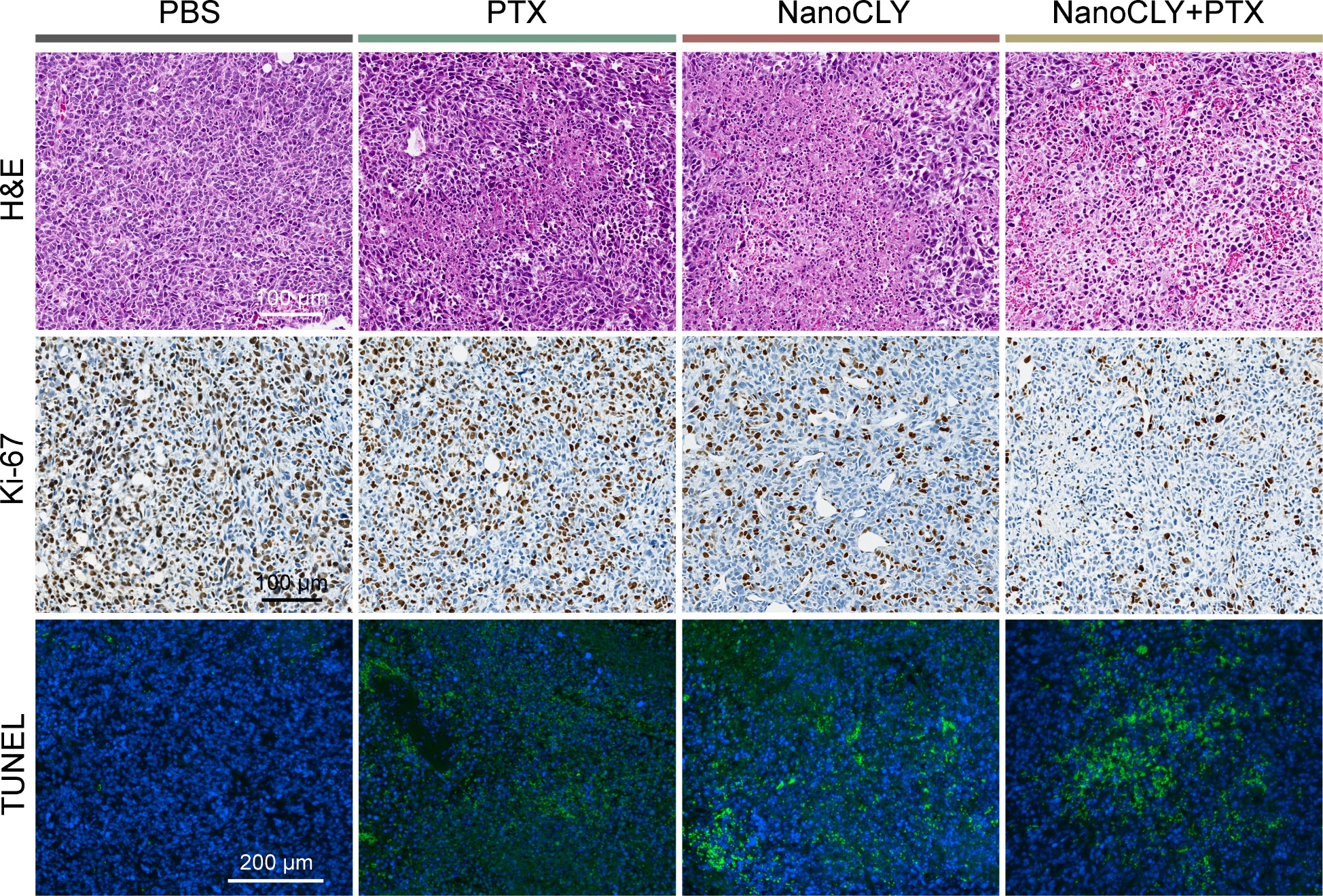
**

**Figure S23.** H&E, Ki-67, and TUNEL staining of tumor samples. Representative images of H&E, Ki-67, and TUNEL staining of MDA-MB-231 tumor samples collected from groups of PBS, PTX, NanoCLY, and NanoCLY+PTX treatment at the end of observation (n = 3).

**
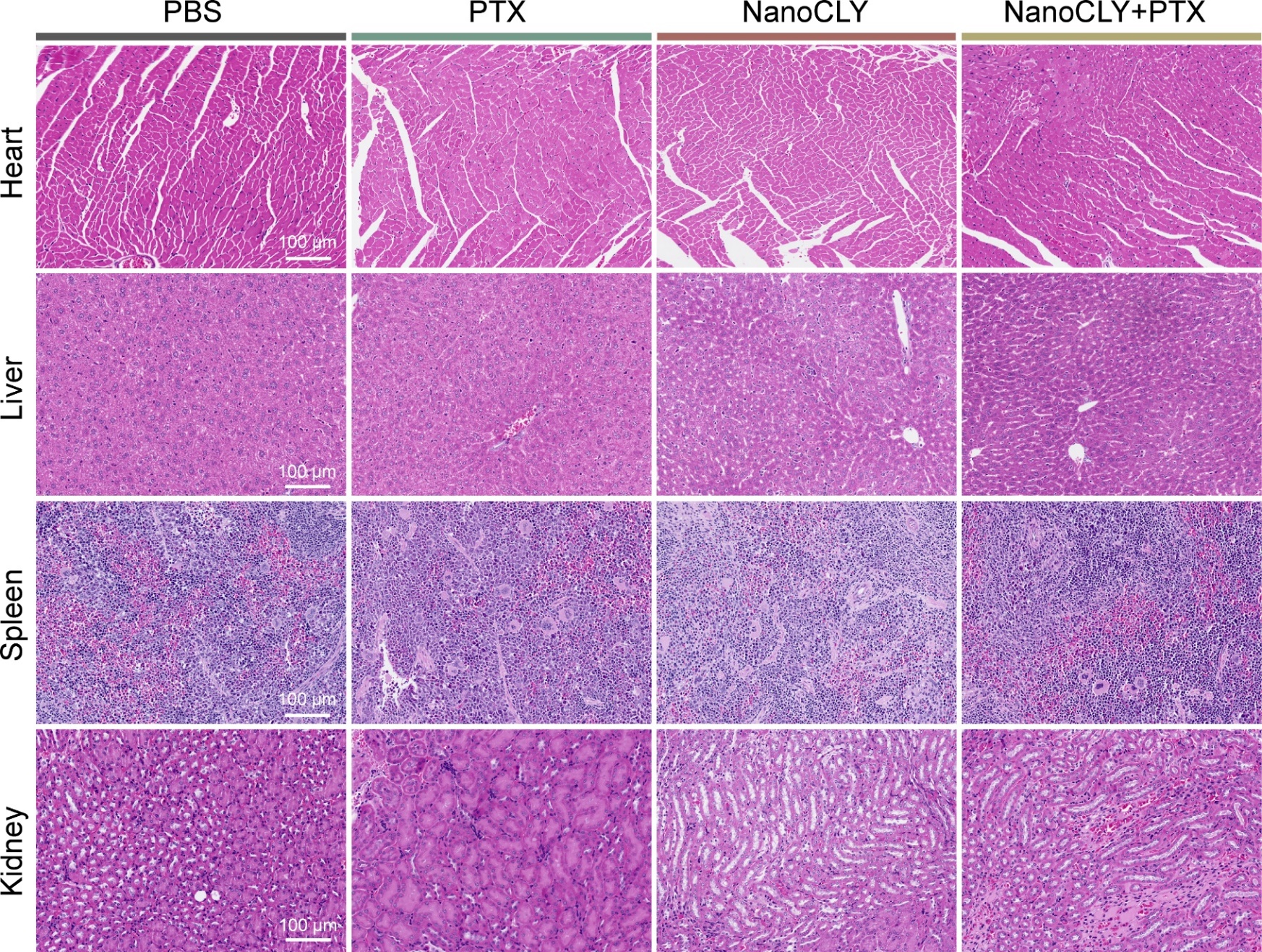
**

**Figure S24.** H&E staining of major organs. Representative images of H&E staining of the heart, liver, spleen, and kidney collected from groups of PBS, PTX, NanoCLY, and NanoCLY+PTX treatment in the MDA-MB-231 orthotopic model at the end of observation (n = 3).

**
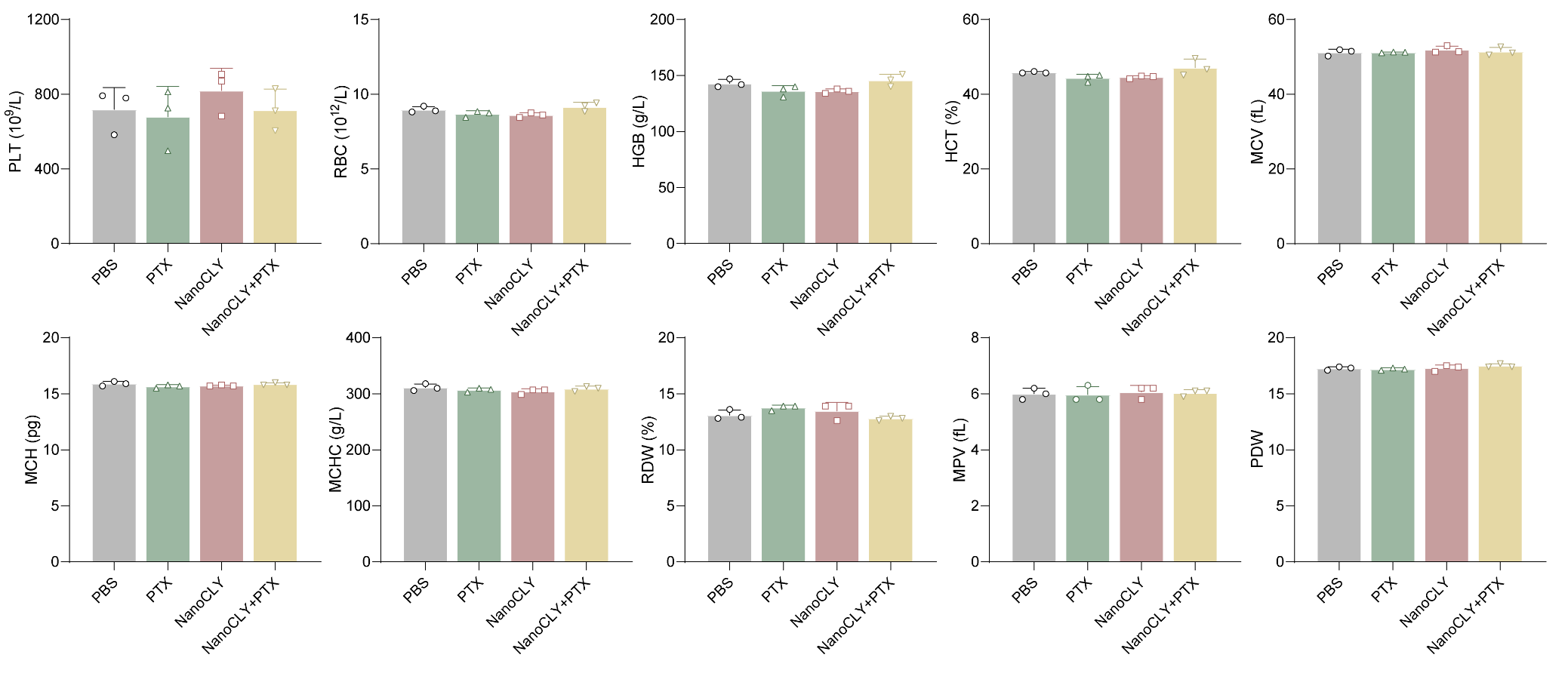
**

**Figure S25.** The complete blood panel analysis. The complete blood panel analysis of the mice bearing MDA-MB-231 tumors at the end of observation (n = 3). PLT, platelet. RBC, red blood cells. HGB, hemoglobin. HCT, hematocrit. MCV, mean corpuscular volume. MCH, mean erythrocyte hemoglobin content. MCHC, mean corpuscular hemoglobin concentration. RDW, red blood cell volume distribution width. MPV, mean platelet volume. PDW, platelet distribution width.

**
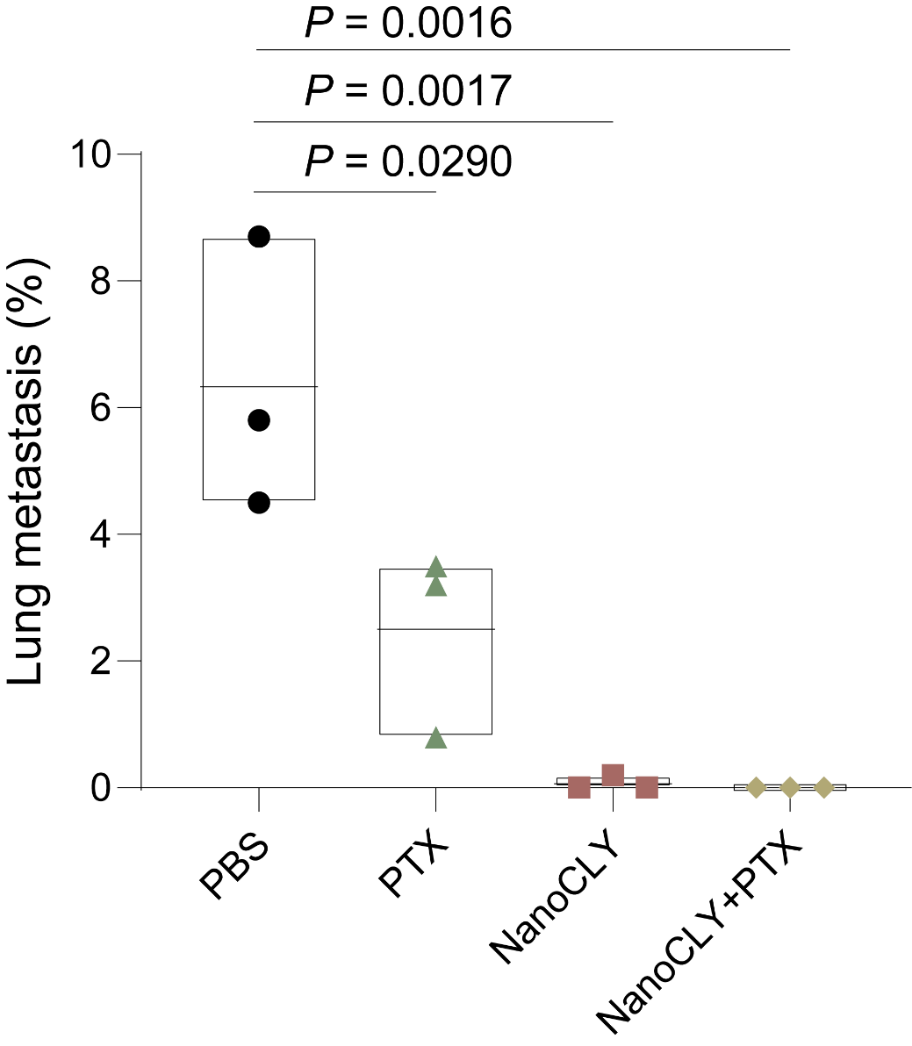
**

**Figure S26.** H&E staining of lungs. Quantitative analysis of H&E staining of lungs at PBS, PTX, NanoCLY, and NanoCLY+PTX groups (n = 3). The quantified data from different experiments were presented as the mean ± SD. The P values were calculated by one-way ANOVA.

**
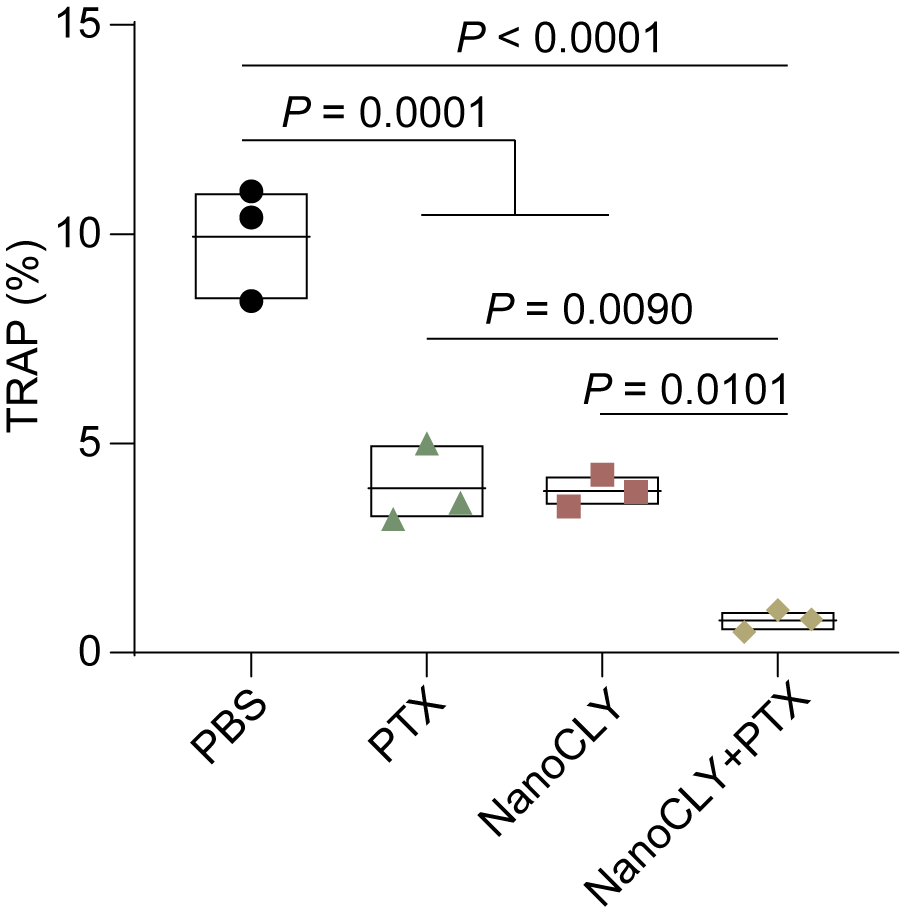
**

**Figure S27.** TRAP ratio in the mouse femoral heads. Relative statistics of the TRAP ratio in the mouse femoral heads at PBS, PTX, NanoCLY, and NanoCLY+PTX groups (n = 3). The quantified data from different experiments were presented as the mean ± SD. The P values were calculated by one-way ANOVA.

# **Table S1.** shRNA sequences.

| **Name** | **sequence** |
| --- | --- |
| sh-Control | sense (5'-3') gatctGTTCTCCGAACGTGTCACGTTTCAAGAGAACGTGACACGTTCGGAGAATTTTTTc |
|  | anti-sense (5'-3') aattgAAAAAATTCTCCGAACGTGTCACGTTCTCTTGAAACGTGACACGTTCGGAGAACa |
| sh-CTGF | sense (5'-3') GATCCGCCCAGACCCAACTATGATTACTCGAGTAATCATAGTTGGGTCTGGGCTTTTTT |
|  | anti-sense (5'-3') AATTAAAAAAGCCCAGACCCAACTATGATTACTCGAGTAATCATAGTTGGGTCTGGGCG |

# **Table S2.** siRNA sequences.

| **Name** | **sequence** |
| --- | --- |
| siNC | sense (5'-3') UUCUUCGAAGGUGUCACGUTT |
|  | anti-sense (5'-3') ACGUGACACGUUCGGAGAATT |
| siCTGF | sense (5'-3') GCUAAAUUCUGUGGAGUAUTT |
|  | anti-sense (5'-3') AUACUCCACAGAAUUUAGCTT |
| siIGF2R-1 | sense (5'-3') GGAGGUGCCAUGCUAUGUGtt |
|  | anti-sense (5'-3') CACAUAGCAUGGCACCUCCtt |
| siIGF2R-2 | sense (5'-3') GGGUUUUCUUUUGACUUAUtt |
|  | anti-sense (5'-3') AUAAGUCAAAAGAAAACCCtg |

# **Table S3.** qRT-PCR primer sequences.

| Gene | Species | Sequence |
| --- | --- | --- |
| GAPDH | Human | Forward 5’-CATGAGAAGTATGACAACAGCCT-3’ |
|  |  | Reverse 5’-AGTCCTTCCACGATACCAAAGT-3’ |
| CTGF | Human | Forward 5’-CTGTGGAGTATGTACCGACGGCC-3’ |
|  |  | Reverse 5’-ATGGCAGGCACAGGTCTTGATGAAC-3’ |
| IL-6 | Human | Forward 5’-ACTCACCTCTTCAGAACGAATTG-3’ |
|  |  | Reverse 5’-CCATCTTTGGAAGGTTCAGGTTG-3’ |
| CXCL8 | Human | Forward 5’-ATACTCCAAACCTTTCCACCC-3’ |
|  |  | Reverse 5’-TCTGCACCCAGTTTTCCTTG-3’ |

# **Table S4.** The sequence, molecular formula, molecular weight, and observed MS of peptides.

| **Peptide** | **Sequence** | **Molecular formula** | **Molecular Weight (g/mol)** | **Observed MS (m/q)** |
| --- | --- | --- | --- | --- |
| Biotin-M6P_1_ | Biotin-PEG_3_-[Pra(M6P)βAla]_1_-NH_2_ | C_35_H_60_N_9_O_17_PS | 941.94 | [M+1H]^1+^=942.40;  [M+2H]^2+^=471.80; |
| Biotin-M6P_2_ | Biotin-PEG_3_-[Pra(M6P)βAla]_2_-NH_2_ | C_51_H_86_N_14_O_28_P_2_S | 1437.33 | [M+1H]^1+^=1438.20;  [M+2H]^2+^=719.20; |
| Biotin-M6P_3_ | Biotin-PEG_3_-[Pra(M6P)βAla]_3_-NH_2_ | C_67_H_112_N_19_O_39_P_3_S | 1932.71 | [M+2H]^2+^=966.90; |
| CL8-M6P_3_ | H-CPSAAHYL-PEG_3_-[Pra(M6P)βAla]_3_-NH_2_ | C_95_H_152_N_27_O_47_P_3_S | 2549.38 | [M+2H]^2+^=1275.70;  [M+3H]^3+^=850.80; |
| NanoCLY | C_12_-CPSAAHYL-PEG_3_-[Pra(M6P)βAla]_3_-NH_2_ | C_107_H_176_N_27_O_47_P_3_S_2_ | 2749.77 | [M+2H]^2+^=1375.50; |
| FITC-CL8-M6P_3_ | FITC-CPSAAHYL-PEG_3_-[Pra(M6P)βAla]_3_-NH_2_ | C_122_H_174_N_29_O_53_P_3_S_2_ | 3051.93 | [M+2H]^2+^=1018.10;  [M+3H]^3+^=763.40; |

Note: H and NH_2_ in the peptide sequence represent the N-terminal amino group and C-terminal primary amide, respectively.

# **References**

1. H. Kawaki, S. Kubota, E. Aoyama, N. Fujita, H. Hanagata, A. Miyauchi, K. Nakai, M. Takigawa, Design and utility of CCN2 anchor peptide aptamers, *Biochimie* **2010**, *92*, 1010.
2. H. Xiao, M. Wang, X. Fan, W. Xu, R. Zhang, G. Wu, A novel peptide binding to the C-terminal domain of connective tissue growth factor for the treatment of bleomycin-induced pulmonary fibrosis, *Int J Biol Macromol* **2020**, *156*, 1464.
3. R. Bruce (Rosalind Franklin university of medicine and science), 2011/123858 A2, **2011**.
